# Promising bactericidal approach of dihydrazone analogues against bio-film forming Gram-negative bacteria and molecular mechanistic studies†

**DOI:** 10.1039/c7ra13661g

**Published:** 2018-01-31

**Authors:** K. P. Rakesh, H. K. Vivek, H. M. Manukumar, C. S. Shantharam, S. N. A. Bukhari, Hua-Li Qin, M. B. Sridhara

**Affiliations:** Department of Pharmaceutical Engineering, School of Chemistry, Chemical Engineering and Life Science, Wuhan University of Technology 205 Luoshi Road Wuhan 430073 PR China qinhuali@whut.edu.cn +86 27 87749300; Analytical Research and Development, Syngene International Ltd Biocon Park, Bommasandra Industrial Estate Bangaluru-560099 Karnataka India; Department of Studies in Biotechnology, University of Mysore Manasagangotri Mysuru-570006 Karnataka India; Department of Chemistry, Pooja Bhagavath Memorial Mahajana Education Centre Mysuru-570016 Karnataka India; Department of Chemistry, Rani Channamma University Vidyasangama Belagavi-591156 Karnataka India sridhara.mb@gmail.com +91 9663983459

## Abstract

Gram-negative members of the ESCAPE family are more difficult to treat, due to the presence of an additional barrier in the form of a lipopolysaccharide layer and the efficiency of efflux pumps to pump out the drugs from the cytoplasm. The development of alternative therapeutic strategies to tackle ESCAPE Gram-negative members is of extreme necessity to provide a solution to the cause of life-threatening infections. The present investigations demonstrated that compounds 17, 20, 25 and 26 possessing the presence of electron donating (OH and OCH_3_) groups on the phenyl rings are highly potent; whereas compounds 9, 10, 15, 16, 18, 33 and 36 showed moderate activity against Gram-negative bacteria. An excellent dose-dependent antibacterial activity was established compared to that of the standard antibiotic ampicillin. Significant anti-biofilm properties were measured quantitatively, showing optical density (O.D) values of 0.51 ± 015, 0.63 ± 0.20, 0.38 ± 0.07 and 0.62 ± 0.11 at 492 nm and the leakage of cellular components by the compounds, such as 17, 20, 25 and 26, increased the O.D. of respective treated samples compared to the control. In addition, the implication of experimental results is discussed in the light of the lack of survivability of planktonic bacteria and biofilm destruction *in vitro*. These results revealed the great significance of the development of a new generation of synthetic materials with greater efficacy in anti-biofilm properties by targeting to lock the bio-film associated protein Bap in Gram-negative bacteria.

## Abstract 2

Gram-negative members of the ESCAPE family are more difficult to treat, due to the presence of an additional barrier in the form of a lipopolysaccharide layer and the efficiency of efflux pumps to pump out the drugs from the cytoplasm.

## Graphical abstract

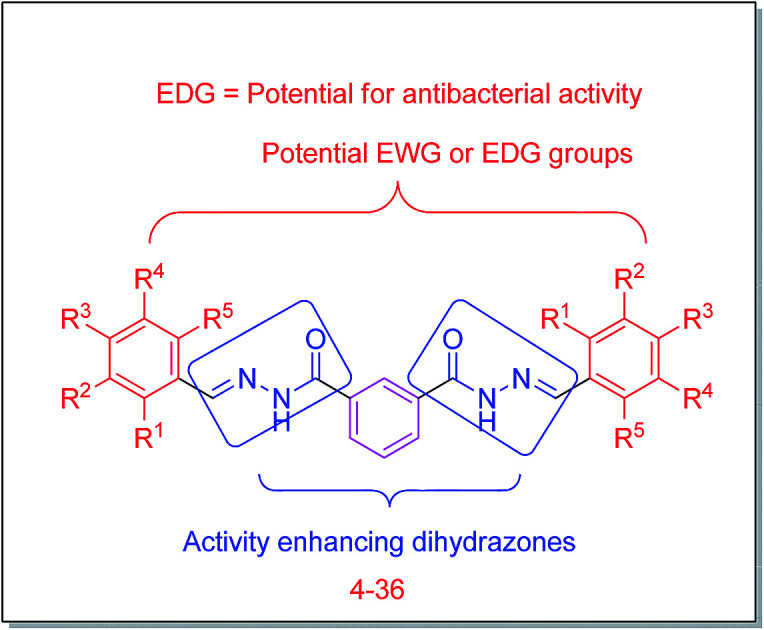

## Introduction

Traditional methods employed to discover new antibiotics are time-consuming and financially-taxing. This has led researchers to mine existing libraries of clinical molecules in order to repurpose old drugs for new applications (as antimicrobials). Bacterial resistance to antibiotics is a significant public health challenge, as infections caused by antibiotic-resistant bacteria claim the lives of nearly 23 000 people each year in the United States alone.^1^ An important quality for an antimicrobial drug is selective toxicity, meaning that it selectively kills or inhibits the growth of microbial targets while causing minimal or no harm to the host. Most antimicrobial drugs currently in clinical use because the prokaryotic cell provides a greater variety of unique targets for selective toxicity, in comparison to fungi, parasites, and viruses. Each class of antibacterial drugs has a unique mode of action (the way in which a drug affects microbes at the cellular level). Based on these facts and the high degree of resistance of Gram-negative bacteria with the development of multidrug-resistant (MDR-resistant to three or more classes of antimicrobials) and extensively drug-resistant (XDR-resistant to one or two classes of antimicrobials) strains, as well as the threatening risk of the development of pan-drug resistant (PDR-resistant to all classes of antimicrobials) strains, researchers have paid particular attention to the infection rate caused by this group.^2,3^ Usually, Gram-negative bacteria are commonly associated with intra-abdominal infections (IAIs), bacteremia, ventilator-associated pneumonia (VAP) and urinary tract infections (UTIs). The main pathogens involved in these infections are *Escherichia coli*, *Klebsiella pneumoniae* and *Pseudomonas aeruginosa*. Together, these species account for 70% of all Gram-negative bacteria causing health care associated infections in the US.^4^

The nosocomial pathogens includes *Acinetobacter baumannii*, *Enterococcus faecium*, *Enterobacter species*, *Klebsiella pneumoniae*, *Pseudomonas aeruginosa*, and *Staphylococcus aureus* are grouped into ESCAPE pathogens due to having overcoming ability to current antimicrobial agents.^5,6^ This shows the behaviour of these pathogens due to occurrence of antimicrobial resistance genes in their genome carried on its chromosome, plasmid or transposons.^7^ These drug resistant determinants fall into broad categories by altering the cell permeability of the drug lead to decrease in the intracellular accumulation, inactivating/altering the drug, altering the target binding site, and forming drug resisting protection layer called biofilm.^8,9^

The prevalence of biofilm formation in infections is estimated to be around 65%, with varying degrees of influence on the course of the disease. Eliminating biofilm-associated bacteria, persisting on catheters, a cardiovascular and orthopedic implant presents a major problem for clinicians. Biofilm formation, which can also be considered as a passive resistance mechanism, is characterized by modified physicochemical microenvironment through the formation of a polysaccharide matrix around bacteria, to inhibit the diffusion of antibiotics (making bacteria incomparably more resistant to them) leading to stationary growth and dormancy (attributed to lower oxygen and nutrient levels), and to make the bacteriostatic agents quasi ineffective.^10–13^

The motivated by special features of dihydrazones analogs and our ongoing research program.^14–20^ With the above background, we aimed towards the development of new therapeutic bacteriostatic agents. Herein the synthesis of dihydrazones and the studies of their biocidal activities were reported and molecular docking study was also conducted for the understanding of the mechanism of the synthetic compounds to their biocidal activities.

## Results and discussion

### Chemistry

**Scheme 1:**
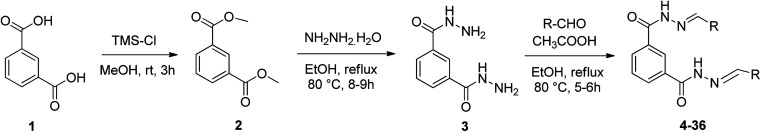
Synthesis of target compounds 4–36.

Syntheses of the dihydrazones were achieved according to the procedures illustrated in Scheme 1. Isophthalic acid (1) was methylated using trimethylsilylchloride (TMS-Cl) and methanol at room temperature to obtain dimethyl isophthalic (2) which upon reaction with an excess of hydrazine hydrate afforded the corresponding isophthalic hydrazide 3.^21^ The obtained hydrazones (4–36) were reacting 3 with different aromatic or aliphatic aldehydes in the presence of catalytic amount of glacial acetic acid. All the derivatives were obtained in excellent yields. The structures of newly synthesized compounds including intermediates were confirmed by ^1^HNMR, ^13^CNMR and mass spectral analysis (ESI†). The formation of methyl esters (2) was confirmed by the appearance of a singlet at 3.83*δ* for –OCH_3_ and absence of COOH proton peak at 12.10*δ* in the ^1^HNMR spectrum. In the ^1^H NMR spectrum of 3, two singlet signals displayed at 9.79 ppm and 4.50 ppm corresponding to the NH and NH_2_ protons, respectively. Moreover, the absence of one signal at 52.3 ppm of –OCH_3_ in ^13^C NMR spectrum confirms the formation of isophthalic hydrazide 3 from methyl ester 2. The final compounds 4–36 were confirmed by their ^1^H NMR, ^13^C NMR, and mass spectrum analysis. ^1^H NMR spectrum of 4 showed a singlet for –NH proton at 12.03 ppm and disappearance of the peak for NH_2_ proton confirms the formation of hydrazones. Further, it showed a singlet at 8.48 ppm for the newly formed azomethine proton (–N=CH–) confirms the formation of the hydrazones. All final compounds 4–36 showed carbonyl (C=O) signal in the region of 162–164 ppm and azomethine carbon (–N=CH–) signal in the region of 146–148 ppm in their ^13^C NMR spectra clearly confirms their formation hydrazones. Further, compound 4 showed a M + 1 peak at 371.2414 in their HRMS confirms the formation of final products.

### Biology

#### Biocidal activity

The antibacterial activities of synthetic compounds were evaluated by disc diffusion method^22,23^ and minimum inhibitory concentration (MIC) was identified by studying different concentrations (1–50 μg mL^−1^) against both Gram-positive (*S. aureus* and *Bacillus cereus*) and Gram-negative (*Escherichia coli*, *Enterobacter aerogenes*, *Salmonella typhimurium* and *Shigella flexneri*) bacteria in the present study (Tables 1–3). The compounds 17, 18, 20, 25 and 26 displayed potent activity against both Gram-positive and Gram-negative bacteria. Among them compound 25 and 26 are highly potent and exhibited strong MIC in μg mL^−1^ against *S. aureus* (22 ± 0.41 and 20 ± 0.62), *B. cereus* (24 ± 0.52 and 22 ± 0.42), *E. coli* (15 ± 0.71 and 16 ± 0.61), *E. aerogenes* (16 ± 0.42 and 17 ± 0.15), *S. typhimurium* (18 ± 0.66 and 16 ± 0.16) and *S. flexneri* (16 ± 0.82 and 17 ± 0.18) compared to standard antibiotic ampicillin (19 ± 0.24, 18 ± 0.72, 16 ± 0.14, 16 ± 0.31, 16 ± 0.42 and 19 ± 0.81 respectively) (Table 4).

**Table 1:** Antibacterial activity of synthesized compounds (1–36) against Gram positive bacteria

Sl. no.	Gram positive (ZoI in mm)											
	*S. aureus*						*B. cereus*					
	1 μg mL^−1^	5 μg mL^−1^	10 μg mL^−1^	15 μg mL^−1^	25 μg mL^−1^	50 μg mL^−1^	1 μg mL^−1^	5 μg mL^−1^	10 μg mL^−1^	15 μg mL^−1^	25 μg mL^−1^	50 μg mL^−1^
1	—	—	—	—	—	—	—	—	—	—	—	—
2	—	—	—	—	—	—	—	—	—	—	—	—
3	—	—	—	—	2	4	—	—	—	—	—	3
4	—	—	2	4	7	9	—	2	3	5	7	9
5	—	2	3	6	8	11	—	—	—	—	2	4
6	—	4	7	10	13	17	—	2	5	7	9	12
7	—	—	3	5	7	10	—	—	—	—	3	7
8	—	—	—	2	5	9	—	—	—	—	—	3
9	2	4	7	12	16	19	—	2	4	7	13	17
10	—	2	6	11	14	17	—	2	7	9	12	15
11	—	—	2	5	6	9	—	—	—	3	5	7
12	—	—	—	2	4	6	—	—	—	—	—	—
13	—	—	—	—	2	5	—	—	2	3	5	8
14	—	—	—	2	4	5	—	—	—	3	5	7
15	3	5	10	13	16	21	—	2	5	8	13	18
16	—	3	8	13	16	19	—	3	7	10	14	17
17	—	7	10	14	18	28	2	5	9	13	17	29
18	—	2	5	8	12	15	—	—	4	7	11	14
19	—	—	3	5	8	11	—	2	5	7	10	13
20	2	6	12	19	24	30	—	—	—	3	5	8
21	—	—	2	3	5	7	—	—	1	3	7	9
22	—	—	—	3	6	9	—	—	—	2	4	7
23	—	—	—	2	4	6	—	—	—	—	3	5
24	—	—	—	2	5	8	—	—	3	4	6	8
25	3	6	10	18	26	31	3	8	15	21	25	30
26	—	2	8	12	17	28	—	4	8	13	16	25
27	—	—	—	—	—	—	—	—	—	—	—	—
28	—	—	—	—	—	—	—	—	—	—	—	—
29	—	—	—	—	—	—	—	—	—	—	—	—
30	—	2	5	8	10	13	—	—	2	3	5	7
31	—	—	—	3	5	8	—	—	2	6	8	10
32	—	4	6	9	11	13	—	—	5	8	10	12
33	—	—	2	8	17	19	—	2	5	7	17	24
34	—	—	2	6	7	9	—	—	—	3	5	8
35	—	—	3	5	8	10	—	—	—	—	3	6
36	—	—	5	8	16	22	—	—	4	8	16	23
A*	4	7	10	18	22	28	4	8	12	17	24	29

**Table 2:** Antibacterial activity of synthesized compounds (1–36) against Gram negative bacteria

Sl. no.	Gram negative											
	*E. coli*						*E. aerogenes*					
	1 μg mL^−1^	5 μg mL^−1^	10 μg mL^−1^	15 μg mL^−1^	25 μg mL^−1^	50 μg mL^−1^	1 μg mL^−1^	5 μg mL^−1^	10 μg mL^−1^	15 μg mL^−1^	25 μg mL^−1^	50 μg mL^−1^
1	—	—	—	—	—	—	—	—	—	—	—	—
2	—	—	—	—	—	—	—	—	—	—	—	—
3	—	—	—	2	3	5	—	—	—	—	1	4
4	—	3	7	10	13	15	—	6	9	12	15	17
5	—	—	2	5	10	14	2	8	12	16	19	22
6	—	3	7	14	18	25	2	6	13	16	20	25
7	—	—	—	5	14	18	—	2	7	14	18	21
8	—	—	—	3	6	13	—	2	6	11	13	18
9	2	5	8	13	19	23	2	8	15	19	22	25
10	—	3	7	10	18	21	—	4	9	13	18	23
11	—	—	4	9	13	17	—	3	5	9	12	15
12	—	—	—	5	9	15	—	3	5	10	13	17
13	—	—	—	2	8	13	—	2	6	11	14	16
14	—	—	3	5	12	17	—	2	5	13	15	18
15	2	5	7	12	17	20	—	6	12	17	19	21
16	0	3	7	13	17	20	2	5	9	15	17	19
17	3	6	10	15	21	25	—	5	7	14	19	24
18	2	5	9	13	19	23	—	4	8	13	16	22
19	—	—	—	5	9	12	—	2	7	12	13	16
20	2	6	12	19	24	29	2	5	13	18	22	26
21	—	—	5	7	11	15	—	2	5	8	12	14
22	—	—	3	5	8	12	—	—	2	9	13	15
23	—	—	—	2	10	13	—	—	3	6	10	12
24	—	—	2	6	9	12	—	—	2	6	12	14
25	2	5	9	13	17	26	2	4	9	18	21	25
26	2	5	7	14	19	25	2	5	7	12	16	24
27	—	—	—	2	5	7	—	—	—	2	5	7
28	—	—	2	5	7	8	—	—	—	3	4	6
29	—	—	4	8	9	10	—	—	—	—	—	3
30	—	—	2	4	8	13	—	5	7	11	14	18
31	—	3	5	7	11	16	—	2	5	7	11	15
32	—	—	4	8	12	17	—	3	5	8	13	18
33	—	2	5	9	17	21	—	—	3	7	18	24
34	—	2	4	6	10	13	—	—	3	6	8	12
35	—	—	4	7	11	14	—	3	8	10	12	15
36	—	3	6	9	19	23	—	—	5	12	21	25
A*	7	10	14	16	20	27	8	10	14	15	19	26

**Table 3:** Antibacterial activity of synthesized compounds (1–36) against Gram negative bacteria

Sl. no.	Gram negative											
	*S. typhimurium*						*S. flexneri*					
	1 μg mL^−1^	5 μg mL^−1^	10 μg mL^−1^	15 μg mL^−1^	25 μg mL^−1^	50 μg mL^−1^	1 μg mL^−1^	5 μg mL^−1^	10 μg mL^−1^	15 μg mL^−1^	25 μg mL^−1^	50 μg mL^−1^
1	—	—	—	—	—	—	—	—	—	—	—	—
2	—	—	—	—	—	—	—	—	—	—	—	—
3	—	—	—	—	—	—	—	—	—	—	—	—
4	2	4	10	14	18	20	—	3	9	13	19	21
5	—	—	4	12	16	23	—	—	5	12	15	23
6	2	3	6	10	12	15	—	3	7	14	19	24
7	—	—	5	8	13	19	—	2	6	10	13	18
8	—	2	5	8	14	20	—	3	6	11	14	21
9	2	4	9	13	19	25	—	4	7	12	18	24
10	—	5	9	14	19	25	2	5	11	16	20	23
11	—	—	—	5	11	18	—	2	4	7	11	16
12	—	3	5	9	12	18	—	—	3	6	12	17
13	—	—	2	6	13	19	—	4	7	11	15	19
14	—	—	3	5	8	12	—	—	2	6	9	14
15	2	4	9	15	19	23	—	4	9	14	19	22
16	—	3	7	12	19	24	—	4	8	12	19	22
17	2	6	12	16	21	27	—	5	11	16	20	25
18	—	5	9	16	20	26	—	2	5	13	19	24
19	—	0	4	9	13	18	—	3	5	7	12	17
20	—	3	8	13	16	22	—	4	7	11	19	26
21	—	2	4	6	11	16	—	3	5	9	13	18
22	—	0	3	7	10	16	—	4	7	12	15	20
23	—	2	4	8	12	18	—	2	5	7	12	17
24	—	2	5	8	13	16	—	3	7	10	13	19
25	3	7	12	16	21	25	3	7	10	15	22	29
26	—	2	7	13	18	21	2	5	11	14	20	28
27	—	—	3	5	9	10	—	2	4	6	9	12
28	—	—	2	7	10	12	—	—	2	7	11	12
29	—	—	3	6	9	11	—	—	3	5	8	10
30	—	—	4	7	11	19	—	4	6	10	14	19
31	—	2	4	7	13	20	—	3	7	10	13	20
32	—	—	3	7	10	15	—	2	5	9	13	18
33	—	—	5	8	16	21	—	2	3	9	17	22
34	—	—	5	8	12	13	—	—	4	8	12	17
35	—	—	—	5	12	19	—	—	5	8	11	19
36	—	—	5	8	17	22	—	3	6	10	19	24
A*	8	10	13	17	20	28	5	9	14	16	20	27

**Table 4:** Minimum inhibitory concentration (MIC) of the synthesized compounds against Gram positive and Gram negative bacteria

Compounds	Gram positive^a^		Gram negative^a^			
	*S. aureus*	*B. cereus*	*E. coli*	*E. aerogenes*	*S. typhimurium*	*S. flexneri*
4	33 ± 0.02	31 ± 0.25	28 ± 0.53	28 ± 0.29	32 ± 0.24	31 ± 0.27
5	42 ± 0.25	39 ± 0.21	26 ± 0.52	25 ± 0.51	26 ± 0.31	28 ± 0.85
6	32 ± 0.21	36 ± 0.37	24 ± 0.52	26 ± 0.44	25 ± 0.32	21 ± 0.54
7	34 ± 0.04	37 ± 0.12	23 ± 0.18	25 ± 0.56	23 ± 0.51	24 ± 0.15
8	31 ± 0.30	30 ± 0.56	25 ± 0.19	27 ± 0.65	25 ± 0.51	26 ± 0.61
9	30 ± 0.12	34 ± 0.18	18 ± 0.16	16 ± 0.54	18 ± 0.52	19 ± 0.32
10	32 ± 0.31	32 ± 0.49	17 ± 0.55	19 ± 0.65	19 ± 0.33	20 ± 0.52
11	32 ± 0.12	33 ± 0.14	23 ± 0.24	22 ± 0.13	22 ± 0.14	23 ± 0.24
12	40 ± 0.33	34 ± 0.85	**19** ± **0.68**	**18** ± **0.61**	**20** ± **0.63**	**21** ± **0.46**
13	35 ± 0.14	31 ± 0.56	26 ± 0.22	27 ± 0.24	26 ± 0.42	24 ± 0.64
14	30 ± 0.22	29 ± 0.43	24 ± 0.23	25 ± 0.32	27 ± 0.85	26 ± 0.73
15	31 ± 0.21	30 ± 0.72	**16** ± **0.65**	**18** ± **0.43**	**18** ± **0.96**	**19** ± **0.35**
16	36 ± 0.41	41 ± 0.11	**17** ± **0.47**	**19** ± **0.52**	**17** ± **0.14**	**18** ± **0.82**
17	**22** ± **0.12**	**20** ± **0.45**	**16** ± **0.51**	**18** ± **0.42**	**19** ± **0.71**	**18** ± **0.18**
18	**21** ± **0.65**	**24** ± **0.31**	**18** ± **0.24**	**17** ± **0.66**	**19** ± **0.27**	**17** ± **0.52**
19	37 ± 0.31	32 ± 0.51	25 ± 0.64	27 ± 0.44	28 ± 0.82	26 ± 0.61
20	**24** ± **0.61**	**26** ± **0.52**	**18** ± **0.71**	**19** ± **0.52**	**16** ± **0.82**	**17** ± **0.42**
21	28 ± 0.35	27 ± 0.63	25 ± 0.61	23 ± 0.51	27 ± 0.34	25 ± 0.34
22	34 ± 0.24	31 ± 0.11	20 ± 0.82	23 ± 0.15	20 ± 0.82	21 ± 0.58
23	33 ± 0.52	33 ± 0.41	21 ± 0.24	22 ± 0.61	23 ± 0.18	22 ± 0.31
24	35 ± 0.52	36 ± 0.16	29 ± 0.42	28 ± 0.52	27 ± 0.61	29 ± 0.51
25	**22** ± **0.41**	**24** ± **0.52**	**15** ± **0.71**	**16** ± **0.42**	**18** ± **0.66**	**16** ± **0.82**
26	**20** ± **0.62**	**22** ± **0.42**	**16** ± **0.61**	**17** ± **0.15**	**16** ± **0.16**	**17** ± **0.18**
27	—	—	—	—	—	—
28	—	—	—	—	—	—
29	—	—	—	—	—	—
30	35 ± 0.62	34 ± 0.65	27 ± 0.61	25 ± 0.81	27 ± 0.17	28 ± 0.61
31	36 ± 0.64	38 ± 0.62	26 ± 0.44	24 ± 0.19	24 ± 0.34	28 ± 0.11
32	30 ± 0.61	31 ± 0.32	25 ± 0.61	26 ± 0.61	29 ± 0.17	30 ± 0.15
33	**28** ± **0.71**	**26** ± **0.55**	**19** ± **0.81**	**18** ± **0.34**	**19** ± **0.04**	**22** ± **0.07**
34	38 ± 0.71	37 ± 0.53	30 ± 0.42	32 ± 0.13	28 ± 0.30	32 ± 0.17
35	30 ± 0.52	31 ± 0.35	28 ± 0.52	26 ± 0.77	29 ± 0.52	30 ± 0.10
36	**26** ± **0.75**	**24** ± **0.43**	**18** ± **0.72**	**18** ± **0.55**	**17** ± **0.13**	**21** ± **0.53**
A*	19 ± 0.24	18 ± 0.72	16 ± 0.14	16 ± 0.31	16 ± 0.42	19 ± 0.81

a Values are mean of three determinations, the ranges of which are <5% of the mean in all cases. A*-standard ampicillin (−): not tested, (±) standard deviation.

#### Anti-biofilm activity

**Fig. 1:**
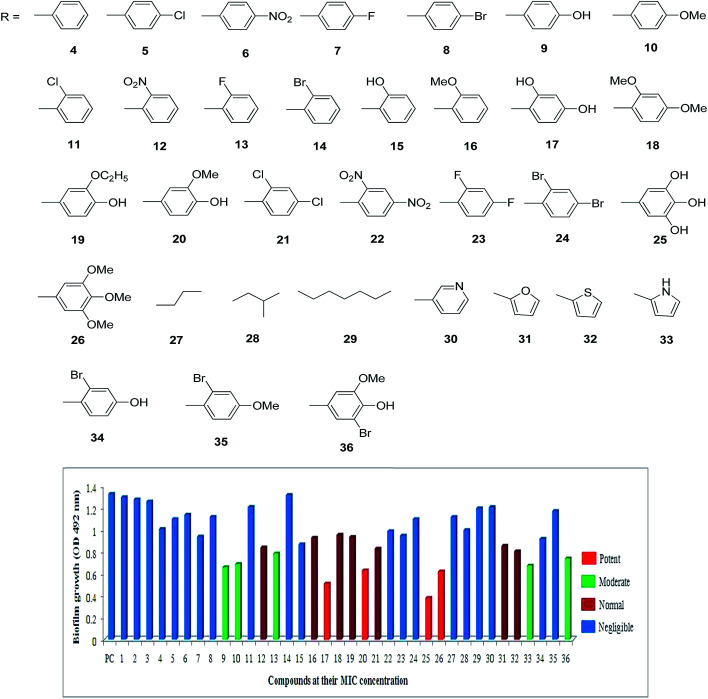
Quantitative determination of anti-biofilm action of synthetic compounds. The inhibitions of biofilm formation by 36 compounds were screened and showed compounds 17, 20, 25, and 26 as potent anti-biofilm activity against *S. flexneri* after 24 h of incubation.

**Fig. 2:**
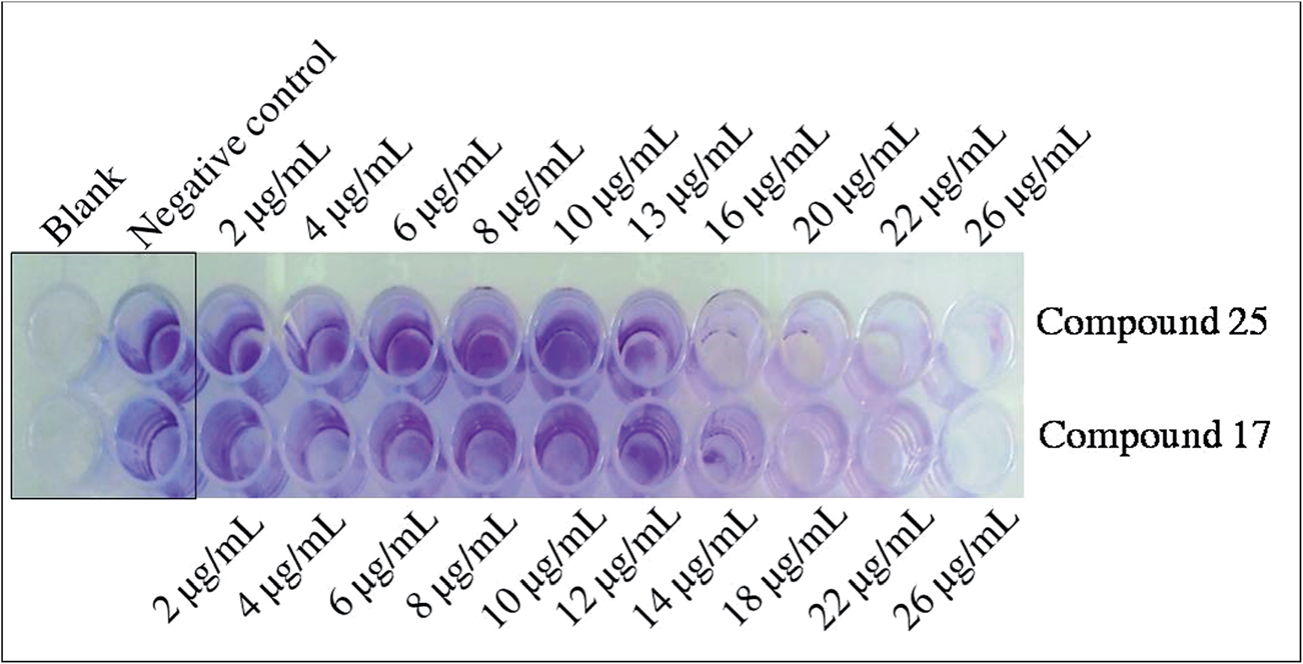
Representation of qualitative determination of anti-biofilm activity. The compound 17 and 25 showed dose dependent inhibition of biofilm formation in 24 h of incubation by *S. flexneri*. The antimicrobial MIC concentration showed clear indication for anti-biofilm formation activity.

In the present study, *S. flexneri* was selected as a model organism to study the anti-biofilm and cellular content release experiments for synthesized compounds. All the 36 compounds were screened for the inhibition of biofilm formation by *S. flexneri* using 96 well plate methods.^24^ Among them, 17, 20, 25 and 26 displayed potent activity, while 9, 10, 13, 33 and 36 showed moderate activity whereas 12, 16, 18, 19, 21, 31 and 32 are less significant activity against biofilm formation compared to negative control (Fig. 1 and 2). The compounds 17 and 25 exhibited highly potent anti-biofilm nature in different concentrations when assessed by crystal violet method of both qualitative and quantitative methods. It is clearly deduced that the MIC action of compounds 17 and 25 played an important role in the active destabilization of membrane destruction of the pathogen which leads to failure of attachment.

#### Cellular content release (CCR)

**Fig. 3:**
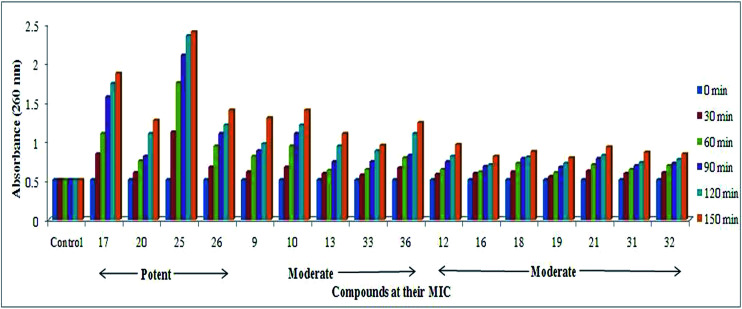
Effect of MIC of compounds on cellular content release from *S. flexneri*. The MIC of compounds treated to bacteria and results obtained in time dependent manner exhibiting release of cell contents when cell membrane was ruptured compared to control.

As shown in Fig. 3, the both Gram-positive and Gram-negative bacteria subjected to evaluate the cell damage caused by compounds in a time-dependent manner at MIC of compounds and demonstrated the significant activity with respect to control. Among all the compounds screened for CCR, compounds 17, 20, 25 and 26 had higher cellular damage compared to others as represented in Fig. 3.^22,23^ Thus, active molecules having potential interaction with the cytoplasmic membrane leads to damage of membrane anatomical structure leads to release of potassium ions, DNA, and other cellular materials were correlated to the highly potent nature of compounds 17, 20, 25 and 26 in time of course was investigated.^23^

#### Scanning electron microscopy (SEM)

**Fig. 4:**
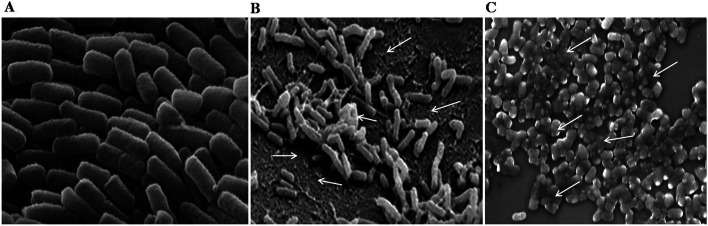
The SEM images of anti-biofilm and cell membrane damage effect of compound 25. (A) The *S. flexneri* control. (B) The anti-biofilm action of compound 25 after 12 h of treatment and arrow indicates the loss of bacterial growth after treatment of compound 25, and (C) indicates the cell membrane damage of bacteria after 24 h of incubation and arrow indicates the cell membrane integrity alterations and anti-bacterial effect of compound 25 compared to control (A).

The potent compound 25 was used to study the action on the cell membrane and biofilm formation of *S. flexneri*. The Fig. 4c showed the MIC concentration of compound 25 involved in cell membrane damage exerted by the compound on cell and decrease in the biofilm formation of *S. flexneri* (Fig. 4b) after the treatment in time of course compared to control.^22^ This evidence clearly indicates that the compounds 25 interacted with the cell membrane and destabilize the membrane integrity to stop the growth of the bacteria.

#### Discussion

Recently there is an emergence of multi-drug resistant pathogenic bacterial strains and most of the available antibiotics are not active against these pathogens.^25,26^ These drug-resistant pathogens are more pathogenic with high mortality rate than that of wild strain. The scientific community is continuously searching for the new classes of disinfection systems that could act efficiently against these pathogens. This study revealed that compounds with electron donating (OH and OCH_3_) groups on the aromatic rings displayed well to excellent antibacterial properties against Gram-positive and Gram-negative bacteria. The four compounds 17, 20, 25 and 26 are leading compounds against Gram-negative bacteria compared to Gram-positive bacteria regardless of other compounds. Clinically, most antibacterials are described as potentially being used as both bactericidal and bacteriostatic. This lead was also observed in the present study by evaluating antibacterial and anti-biofilm properties of tested compounds. Even if a bactericidal action is preferred in the context of treatment, achieving a bacteriostatic effect may advantageously inhibit the pathogen infection.^27^ The MIC of compound 25 treated to *S. flxneri* and observed for cell membrane integrity, which was altered to the damage of cell membrane in the Fig. 4a. This factor leads to great loss of cell architectural integrity and leakage of critical metabolic molecules, vital ions which regulate the cell division rates ultimately leading to bacterial cell death.^28,29^ The microorganisms growing on inert or living surfaces usually form microbial biofilms, which were formed by dense communities of microbial cells surrounded with the self-secreted matrix. Biofilm development is one of the bacterial defense strategies to survive at different conditions. The present study revealed that the MIC of 17, 25 and 26 exhibited anti-biofilm property against *S. fixneri* as a model organism. The failure in the prevention and eradication of microbial biofilms might create a number of serious problems such as bio-deterioration, food contamination and infectious diseases such as endocarditis, periodontitis and chronic lung infections in cystic fibrosis patients being the prominent ailments.^30^ The significant results of potent compounds in the present study can be observed through the cell membrane damage, integrity and cell architectural variation in Fig. 4. The inhibition of bacteria and biofilm formation is an interesting way to prevent the formation of well-organized attached bacterial biofilms and pathogenesis.^27^ Considering the potential clinical application of our study, additional experiments could be conducted on the combination of natural antibacterial agents and currently used antibiotics to enhance the present management practices against Gram-negative bacteria.

To study the structure activity relationship, the substituents on the phenyl rings play a major role in the antibacterial activity. Compounds 17, 18, 20, 25 and 26 were found to have excellent antibacterial activity. The presence of electron donating (OH and OCH_3_) groups present on the phenyl ring, increases the antibacterial activity. Compounds 5–8, 11–14 and 21–24 were found to possess moderate antibacterial activity against the all tested Gram positive and Gram negative bacterial strains. The presences of electron withdrawing (Cl, NO_2_, Br, and F) groups on the phenyl rings reduce the antibacterial activity. Whereas, compounds containing aliphatic (27–29) analogs were displayed nil activities. It is interesting to find that the more electron donating groups present on the phenyl ring the better antibacterial activity was observed.

### Molecular docking

**Fig. 5:**
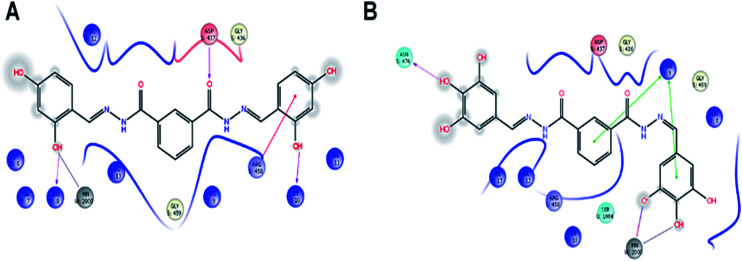
Molecular docking interactive map of compound 17 (A) and 25 (B) into the DNA Gyrase, binding deep inside the active site, depicting the best docking pose.

**Fig. 6:**
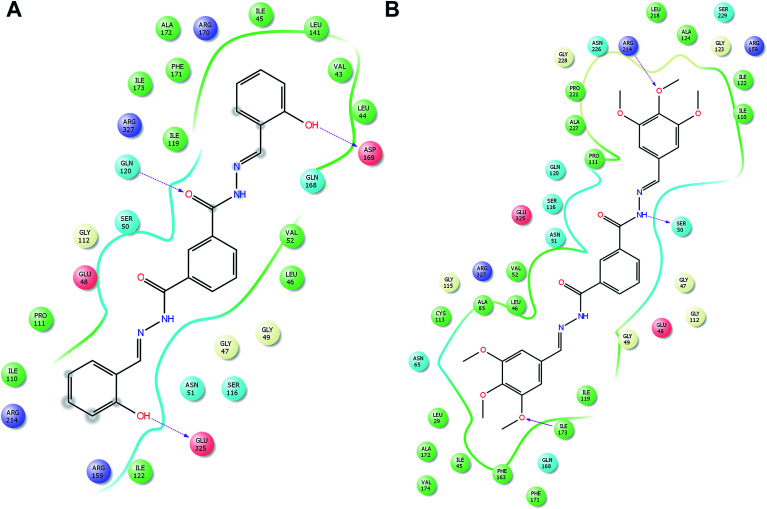
Molecular docking interactive map of compound 15 (A), and 26 (B) into the MurB, binding deep inside the active site, depicting the best docking pose.

In order to understand possible mechanisms by which the synthesized compounds exerted their antibacterial activity, the molecular docking study was conducted.^31^ The docking studies demonstrated an interacting map of DNA Gyrase from *Staphylococcus aureus* complex, then compound 17 interacting with Asp437 *via* a hydrogen bond, metal coordinate with manganese and with Arg458 it forms a salt bridge, these interactions are tightly bound to DNA (Fig. 5A). Whereas the compound 25, also forms a tight interaction with Asn476 *via* a hydrogen bond and metal coordinate with manganese (Fig. 5B) these interactions suggest that the cofactor Manganese, the is chelated, which inhibit the accessibility for DNA polymerase, thereby inhibiting the further growth of *Staphylococcus aureus*. The peptidoglycan biosynthesis begins with the action of two enzymes *viz.*, MurA and MurB, with MurB catalyzing the second step in the formation of muramyl sugar. Hence inactivation of MurB causes inhibition of bacterial cell wall synthesis. In addition to the interaction of compound 15 Glu120, Asp169, and Glu325 *via* hydrogen bond (Fig. 6A) whereas compound 26 forms a hydrogen bond with Arg 214, Ile173 and Ser50 (Fig. 6B). These amino acids are crucial and reside at the active site of the enzyme, which help in biosynthesis of peptidoglycan, any perturbation in these amino acids could down-regulate the enzyme activity. Overall docking result suggest that hydroxyl group present on *ortho* (15), *ortho*–*para* (17) and *ortho*–*para*–*meta* (25) position is more favorable. Whereas with methoxy group, present at *ortho*–*para*–*meta* fit into active site of enzyme. Based on XP glide score, compounds 15, 17, 25 and 26 displayed promising G-scoring functions, when compared to other structurally related compounds as tabulated in Table 5. Noteworthy, these data validate that compounds 15, 17, 25 and 26 is comparatively potent against standard antibiotic.

**Table 5:** Molecular docking scores of all the synthesized compounds against DNA Gyrase and MurB as obtained through Glide docking

Entry	2XCT					1MBT				
	RMSD-OPLS-2005	Docking score	Glide G-score	Glide E-model	Glide energy	RMSD-OPLS-2005	Docking score	Glide G-score	Glide E-model	Glide energy
R1	0.006	−7.451	−7.452	−49.96	−20.959	0.006	−7.105	−7.106	−48.427	−27.663
R2	0.001	−5.97	−5.97	−53.784	−37.654	0.001	−5.356	−5.356	−43.412	−33.114
R3	0.03	−5.972	−5.972	−59.317	−41.435	0.03	−4.994	−4.994	−44.553	−33.901
R4	—	—	—	—	—	0.006	−5.12	−5.121	−30.307	−33.587
R5	0.043	−4.434	−4.435	−51.844	−45.592	0.014	−1.496	−1.498	−7.811	−13.048
R6	0.014	−6.332	−6.333	−34.817	−41.121	0.011	−1.093	−1.094	6.57	−6.37
R7	0.011	−4.674	−4.675	−51.65	−44.773	0.034	−3.179	−3.191	−48.633	−40.844
R8	0.011	−4.232	−4.233	−51.818	−46.244	—	—	—	—	—
R9	0.034	−4.414	−4.426	−44.637	−40.984	0.026	−4.885	−4.886	−50.456	−46.619
R10	—	—	—	—	—	0.014	−6.03	−6.031	−27.401	−36.417
R11	0.026	−4.69	−4.691	−56.647	−48.568	0.005	−3.525	−3.526	−36.542	−39.4
R14	0.005	−4.467	−4.468	−55.309	−50.12	0.031	−7.313	−7.372	−46.61	−59.776
R15	0.031	−7.551	−7.611	−59.018	−41.914	0.044	−3.207	−3.208	−29.191	−39.168
R16	0.044	−4.396	−4.397	−34.725	−34.372	0.004	−4.753	−4.824	−65.845	−43.599
R17	0.004	−6.85	−6.92	−62.054	−51.797	0.01	−7.91	−7.911	−44.692	−44.213
R18	—	—	—	—	—	0.021	−3.636	−3.636	−58.385	−48.255
R19	0.021	−5.035	−5.035	−66.84	−56.656	0.042	−3.228	−3.24	−52.546	−44.204
R20	0.042	−6.979	−6.991	−60.042	−52.091	0.005	−7.396	−7.391	−48.171	−50.495
R21	0.040	−5.950	−5.920	−58.022	−58.291	0.002	−5.398	−5.641	−47.151	−52.475
R22	0.01	−4.955	−4.956	−77.14	−60.494	0.01	−4.244	−4.246	−35.217	−44.547
R23	0.021	−4.044	−4.045	−56.613	−49.522	0.021	−5.06	−5.061	−48.827	−44.46
R24	0.005	−5.352	−5.354	−38.782	−42.542	0.005	−6.593	−6.594	−84.32	−63.562
R25	0.018	−6.697	−6.751	−73.632	−42.803	0.013	−7.237	−7.293	−53.454	−44.765
R26	0.020	−6.690	−6.697	−45.705	−45.302	0.028	−7.511	−7.512	−50.895	−63.549
R27	0.006	−3.434	−3.488	−62.445	−51.999	0.006	−3.929	−3.984	−55.161	−45.311
R28	0.01	−4.809	−4.86	−51.594	−41.07	0.043	−4.331	−6.218	−65.353	−48.254
R29	0.05	−5.24	−5.294	−79.967	−62.3	0.05	−5.499	−5.552	−67.599	−53.394
R30	0.007	−5.325	−5.435	−40.033	−35.282	0.007	−5.266	−5.376	−38.255	−36.778
R31	0.004	−5.808	−5.88	−57.928	−49.706	0.004	−4.609	−4.681	−56.333	−49.4
R32	0.02	−5.686	−5.836	−64.46	−52.57	0.02	−4.878	−5.028	−52.878	−43.787
R33	0.002	−4.443	−4.553	−28.984	−31.941	0.002	−5.353	−5.463	−60.691	−50.675
R34	0.013	−3.984	−5.633	−33.271	−34.307	0.026	−4.097	−5.746	−63.372	−51.775
R35	—	—	—	—	—	0.007	−4.23	−4.232	−58.572	−50.928
R36	0.012	−4.134	−5.107	−63.6	−54.392	0.042	−3.114	−3.418	−56.533	−48.925
Ampicillin	0.006	−5.611	−5.668	−61.036	−44.776	0.006	−6.331	−6.388	−53.944	−44.146

## Conclusion

The Infectious Diseases Society of America (IDSA) has identified a few bacterial species as the most threatening pathogens due to the rapid development of antibiotic resistance in those species. The ESCAPE, as the name suggests, this class of pathogens can effectively escape the bactericidal effect of most of the conventional antibiotics especially due to the presence of exclusive permeability barriers and efflux pumps in Gram-negative pathogens. To address this, we designed synthetic analogs to treat Gram-negative bacteria in the present investigation. The analogs 17, 20, 25 and 26 are potent in nature for Gram-negative compared to Gram-positive bacteria. The excellent antibacterial, anti-biofilm and cell membrane damaging property of potent molecules, penetrate in to the microbial surface to kill the life-threatening agents in the study. We reported special highly potent molecules 17, 25 and 26 as broad-spectrum antibiotic agents against resistant ESCAPE pathogens. Further structural modification and alteration increase antibacterial resistance towards the development of a new generation of antibiotics against ESCAPE pathogens was warranted for the future research. Molecular docking studies were performed for all the synthesized compounds among which compounds 15, 17, 25 and 26 showed the highest docking G-scores for antibacterial activity.

## Conflicts of interest

There are no conflicts to declare.

## Supplementary Material

RA-008-C7RA13661G-s001
