# Supplementary material for: Promising bactericidal approach of dihydrazone analogues against bio-film forming Gram-negative bacteria and molecular mechanistic studies
Source: RSC Adv. 2018 Jan 31;8(10):5473–83. doi: 10.1039/c7ra13661g (PMC9078102; doi:10.1039/c7ra13661g)
Supplement: RA-008-C7RA13661G-s001 [file RA-008-C7RA13661G-s001.pdf]

Electronic Supplementary Material (ESI) for RSC Advances.
This journal is © The Royal Society of Chemistry 2018

Supplementary material

Promising bactericidal approach of dihydrazone analogues against bio-film forming Gram-negative bacteria and molecular mechanistic studies

K. P. Rakesh[1*], H. K. Vivek[2], H. M. Manukumar[3], C. S. Shantharam[4], S. N. A. Bukhari[1], Hua-Li Qin[1*] and M. B. Sridhara[5*]

[1]Department of Pharmaceutical Engineering, School of Chemistry, Chemical Engineering and Life Science, Wuhan University of Technology, 205 Luoshi Road, Wuhan, 430073, PR China

[2]Analytical Research and Development, Syngene International Ltd, Biocon Park, Bommasandra Industrial Estate, Bangaluru-560099, Karnataka, India

[3]Department of Studies in Biotechnology, University of Mysore, Manasagangotri, Mysuru-570006, Karnataka, India

[4]Department of Chemistry, Pooja Bhagavath Memorial Mahajana Education Centre, Mysuru-570016, Karnataka, India

[5]Department of Chemistry, Rani Channamma University, Vidyasangama, Belagavi-591156, Karnataka, India

***Corresponding authors**

Email: qinhuali@whut.edu.cn

Fax: +86 27 87749300.

E-mail: sridhara.mb@gmail.com

Tel no: +91 9663983459

Experimental

Chemistry

All other chemicals and reagents obtained from Merck (India) and Avra Synthesis (India) were used without further purification. Melting points were determined on a Superfit melting point apparatus (India) and are uncorrected. ^{1}H NMR (400 MHz) and ^{13}C NMR (100 MHz) spectra were recorded on an Agilent Technologies (USA) using DMSO (d_6) as a solvent. The high-resolution mass spectroscopic analysis was performed on a Bruker MicroTOF QII mass spectrometer in positive mode. The progress of the reaction was

monitored by TLC using silica gel coated on glass plates with the solvent system comprising chloroform/ methanol/acetic acid in the ratio 95:05:03 (R_f^a) and the compounds on the TLC plates were detected by under UV light.

Experimental procedure for the synthesis of 2: To a solution of isophthalic acid (0.06 mol, 10.0 g) dissolved in methanol (100 mL), trimethylsilylchloride (0.06 mol, 7.6 mL) was added slowly. The reaction mixture was stirred for 3 hrs to complete the reaction (monitored by TLC). The solvent was removed under reduced pressure and the resultant precipitate was washed with ice-cold water and filtered to yield the desired product **2.**

Experimental procedure for the synthesis of 3:[1] To a solution of **2** (0.051 mol, 10 g) dissolved in ethanol (100 mL), hydrazine hydrate (0.077 mol, 3.85 g) was added. The reaction mixture was refluxed for 8-10 hrs for completion of the reaction (monitored by TLC). The solvent was removed under reduced pressure and cooled by adding ice cold water. The resulting precipitate was filtered, washed with cold water and recrystallized from ethanol to get the desired compounds **3**.

General procedure for the synthesis of dihydrazones (4-36): Compound **3** (1 mmol) was dissolved in 3-4 mL of ethanol and treated with appropriate aldehydes (2 mmol) in the presence of 5 to 6 drops of glacial acetic acid. The reaction mixtures were refluxed for 7–8 hr and the completion of the reaction was monitored by TLC. After completion of the reaction, the solvent was removed under reduced pressure and cooled by adding ice cold water. The resulting precipitate was filtered, washed with water and recrystallized from ethanol to obtain the desired dihydrazones **(4-36)**.

Characterization:

Dimethyl isophthalate (2)

Yield 94%, $R_f^a = 0.58$, m.p. 68-69 °C, IR KBr (cm^{-1}): 1651, 1760, 3250, 3259, 3322, 3335; ^{1}H NMR (DMSO-d_6) δ H: 3.89 (6H, s, $(OCH_3)_2$), 7.56 (1H, t, J= 8.3 Hz, ArH), 8.24-8.26

(3H, m, J= 18.6 Hz, ArH); ^{13}C NMR (DMSO-d_6) δ ppm: 51.5, 127.6, 129.6, 131.6, 134.2, 164.6; HRMS m/z [M+1]: 195.1887

Isophthalohydrazide (3)

Yield 88%, R_f^a = 0.36, m.p. 232-233 °C, (lit 232-234 °C),[1] IR KBr (cm^{-1}): 1610, 1725; ^{1}H NMR (DMSO-d_6) δ H: 4.50 (4H, s, 2NH_2), 7.49 (1H, t, J = 7.6 Hz, ArH), 7.88 (2H, d, J= 7.6 Hz, ArH), 8.23 (1H, s, ArH), 9.79 (2H, s, 2NH); ^{13}C NMR (DMSO-d_6) δ ppm: 126.4, 128.8, 129.7, 133.9, 165.8; HRMS m/z [M+1]: 195.1387

Dibenzylideneisophthalohydrazide[2] (4)

Yield 92%, R_f = 0.54, m.p. 282-284 °C, (lit >270 °C)[2], IR KBr (cm^{-1}): 1607, 1616, 1642, 1748, 3247, 3269; ^{1}H NMR (DMSO-d_6) δ H: 7.43 (6H, d, J= 6.8 Hz, ArH), 7.65-7.74 (5H, m, ArH), 8.11 (2H, d, J= 7.2 Hz, 2-N=CH), 8.48 (3H, s, ArH), 12.09 (2H, s, 2NH); ^{13}C NMR (DMSO-d_6) δ ppm: 127.4, 127.6, 128.1, 129.3, 130.6, 131.2, 134.1, 134.6, 148.6, 163.0; HRMS m/z [M+1]: 371.2414

Bis(4-chlorobenzylidene)isophthalohydrazide (5)

Yield 91%, R_f = 0.50, m.p. 236-238 °C, IR KBr (cm^{-1}):1610, 1612, 1685, 1741, 3245, 3320; ^{1}H NMR (DMSO-d_6) δ H: 7.50 (4H, d, J = 8.0 Hz, ArH), 7.67 (1H, t, J = 7.6 Hz, ArH), 7.74 (4H, d, J = 8.4 Hz, ArH), 8.10 (2H, s, 2-N=CH), 8.44 (3H, s, ArH), 12.08 (2H, s, 2NH); ^{13}C NMR (DMSO-d_6) δ ppm: 127.4, 129.2, 129.3, 131.3, 133.6, 134.0, 135.0, 147.3, 163.0; HRMS m/z [M+]: 439.1682

Bis(4-nitrobenzylidene)isophthalohydrazide[3] (6)

Yield 91%, R_f = 0.49, m.p. 220-222 °C, IR KBr (cm^{-1}): 1608, 1613, 1721, 1734, 3285, 3326; ^{1}H NMR (DMSO-d_6) δ H: 7.69 (1H, d, J = 8.4 Hz, ArH), 7.99 (4H, d, J = 8.4 Hz, ArH), 8.13 (2H, d, J = 7.6 Hz, ArH), 8.29 (4H, d, J = 8.4 Hz, ArH), 8.48 (1H, d, J = 8.0 Hz, ArH), 8.55 (2H, s, 2-N=CH), 12.03 (2H, s, 2NH); ^{13}C NMR (DMSO-d_6) δ ppm: 124.2, 124.5, 128.5, 131.5, 133.8, 140.9, 146.1, 148.3, 163.2; HRMS m/z [M+1]: 461.2206

Bis(4-fluorobenzylidene)isophthalohydrazide (7)

Yield 89%, R_f = 0.54, m.p. 272-273 °C, IR KBr (cm^{-1}): 1608, 1625, 1752, 1769, 3269, 3314; ^1H NMR (DMSO-d_6) δ H: 7.38 (1H, d, J = 7.2 Hz, ArH), 7.86 (4H, d, J = 7.8 Hz, ArH), 8.20 (2H, d, J = 6.8 Hz, ArH), 8.30 (4H, d, J = 6.8 Hz, ArH), 8.44 (1H, d, J = 8.2 Hz, ArH), 8.54 (2H, s, 2-N=CH), 12.12 (2H, s, 2NH); ^{13}C NMR (DMSO-d_6) δ ppm: 115.3, 125.6, 128.4, 129.3, 130.1, 130.9, 134.6, 148.6, 161.5, 163.5; HRMS m/z [M+1]: 407.3845

Bis(4-bromobenzylidene)isophthalohydrazide (8)

Yield 92%, R_f = 0.56, m.p. 210-214 °C, IR KBr (cm^{-1}): 1610, 1618, 1752, 1769, 3214, 3269; ^1H NMR (DMSO-d_6) δ H: 7.63-7.69 (8H, m, ArH), 8.09 (2H, d, J = 7.2 Hz, ArH), 8.13 (2H, d, J = 7.6 Hz, ArH), 8.43 (2H, s, 2-N=CH), 12.07 (2H, s, 2NH); ^{13}C NMR (DMSO-d_6) δ ppm: 123.8, 127.4, 129.2, 129.4, 131.3, 132.3, 133.9, 134.0, 147.4, 163.0; HRMS m/z [M+1]: 529.0669

Bis(4-hydroxybenzylidene)isophthalohydrazide (9)

Yield 88%, R_f = 0.40, m.p. 246-248 °C, IR KBr (cm^{-1}): 1612, 1620, 1752, 1788, 3210, 3256, 3510, 3547; ^1H NMR (DMSO-d_6) δ H: 6.83 (4H, d, J = 8.4 Hz, ArH), 7.56 (4H, d, J = 8.4 Hz, ArH), 7.63 (1H, t, J = 7.2 Hz, ArH), 8.07 (2H, d, J = 8.0Hz, ArH), 8.36 (2H, s, 2-N=CH), 8.43 (1H, d, J = 8.0 Hz, ArH), 9.94 (2H, s, 2OH), 11.82 (2H, s, 2NH); ^{13}C NMR (DMSO-d_6) δ ppm: 116.1, 125.6, 127.2, 129.1, 131.0, 134.3, 148.9, 159.9, 162.8; HRMS m/z [M+1]: 403.2336

Bis(4-methoxybenzylidene)isophthalohydrazide (10)

Yield 90%, R_f^a = 0.51, m.p. 216-218 °C, IR KBr (cm^{-1}): 1608, 1616, 1742, 1755, 3214, 3269; ^1H NMR (DMSO-d_6) δ H: 3.78 (6H, s, $2OCH_3$), 7.00 (4H, d, J = 8.4 Hz, ArH), 7.66 (5H, t, J = 8.4 Hz, ArH), 8.08 (2H, d, J = 7.6 Hz, ArH), 8.42 (3H, d, J = 11.6 Hz, 1ArH and 2-N=CH), 11.89 (2H, s, 2NH); ^{13}C NMR (DMSO-d_6) δ ppm: 55.7, 114.8, 127.2, 127.2, 128.1, 129.2, 131.0, 134.3, 148.5, 161.3, 162.9; HRMS m/z [M+1]: 431.2674

Bis(2-chlorobenzylidene)isophthalohydrazide (11)

Yield 89%, $R_f = 0.52$, m.p. 270-272 °C, IR KBr (cm^{-1}): 1608, 1628, 1742, 1759, 3248, 3269; ^1H NMR (DMSO-d_6) δ H: 7.40-7.50 (6H, m, ArH), 7.69 (1H, t, J = 7.2 Hz, ArH), 8.20 (2H, s, ArH), 8.14 (2H, d, J = 7.2 Hz, ArH), 8.15 (1H, s, ArH), 8.88 (2H, s, 2-N=CH), 12.22 (2H, s, 2NH); ^{13}C NMR (DMSO-d_6) δ ppm: 125.3, 127.3, 128.0, 129.3, 130.3, 131.4, 131.9, 132.0, 133.7, 133.9, 144.5, 163.0; HRMS m/z [M+]: 439.1774

Bis(2-nitrobenzylidene)isophthalohydrazide (12)

Yield 87%, $R_f = 0.48$, m.p. 288-290 °C, IR KBr (cm^{-1}): 1614, 1622, 1725, 1762, 3256, 3285; ^1H NMR (DMSO-d_6) δ H: 7.65-7.70 (3H, m, ArH), 7.81 (2H, t, J = 7.6 Hz, ArH), 8.07 (2H, d, J = 8.4 Hz, ArH), 8.13 (4H, t, J = 6.4 Hz, ArH), 8.50 (1H, s, ArH), 8.89 (2H, s, 2-N=CH), 12.37 (2H, s, 2NH); ^{13}C NMR (DMSO-d_6) δ ppm: 125.1, 127.5, 128.4, 129.1, 129.3, 131.2, 131.6, 133.7, 134.2, 143.9, 148.7, 163.1; HRMS m/z [M+1]: 461.2300

Bis(2-fluorobenzylidene)isophthalohydrazide (13)

Yield 86%, $R_f = 0.52$, m.p. 256-258 °C, IR KBr (cm^{-1}): 1612, 1619, 1741, 1756, 3210, 3258; ^1H NMR (DMSO-d_6) δ H: 7.26 (2H, m, ArH), 7.31 (1H, m, ArH), 7.80 (2H, t, J = 7.8 Hz, ArH), 8.10 (2H, d, J = 7.8 Hz, ArH), 8.17 (4H, m, ArH), 8.34 (1H, s, ArH), 8.43 (2H, s, 2-N=CH), 12.12 (2H, s, 2NH); ^{13}C NMR (DMSO-d_6) δ ppm: 115.118.6, 123.6, 125.6, 128.9, 129.5, 130.2, 131.5, 132.6, 134.6, 159.3, 163.8; HRMS m/z [M+1]: 407.1203

Bis(2-bromobenzylidene)isophthalohydrazide (14)

Yield 92%, $R_f = 0.56$, m.p. 240-242 °C, IR KBr (cm^{-1}): 1610, 1627, 1740, 1768, 3244, 3310; ^1H NMR (DMSO-d_6) δ H: 7.45-7.47 (2H, m, ArH), 7.52-7.53 (3H, m, ArH), 7.75 (2H, d, J = 6.8 Hz, ArH), 8.16 (4H, t, J = 7.2 Hz, ArH), 8.30 (1H, s, ArH), 8.44 (2H, s, 2-N=CH), 12.16 (2H, s, 2NH); ^{13}C NMR (DMSO-d_6) δ ppm: 122.6, 124.9, 127.3, 127.9, 128.3, 130.1, 130.9, 132.6, 134.7, 136.1, 143.8, 163.5; HRMS m/z [M+1]: 529.1209

Bis(2-hydroxybenzylidene)isophthalohydrazide[4] (15)

Yield 86%, R_f = 0.38, m.p. 290-291 °C, (lit >270 °C), IR KBr (cm^{-1}): 1607, 1618, 1742, 1755, 3210, 3325, 3562, 3670; ^1H NMR (DMSO-d_6) δ H: 6.88-6.93 (4H, m, ArH), 7.28 (2H, t, J = 6.8 Hz, ArH), 7.55 (2H, d, J = 7.6 Hz, ArH), 7.69 (1H, t, J = 7.6 Hz, ArH), 8.13-8.15 (2H, m, ArH), 8.52 (1H, s, ArH), 8.67 (2H, s, 2-N=CH), 11.24 (2H, s, 2OH), 12.28 (2H, s, 2NH); ^{13}C NMR (DMSO-d_6) δ ppm: 116.8, 119.1, 119.8, 127.4, 129.3, 129.8, 131.4, 131.9, 133.6, 149.0, 157.9, 162.7; HRMS m/z [M+1]: 403.2336

Bis(2-methoxybenzylidene)isophthalohydrazide (16)

Yield 90%, R_f = 0.51, m.p. 216-218 °C, IR KBr (cm^{-1}): 1610, 1618, 1652, 1762, 3210, 3218; ^1H NMR (DMSO-d_6) δ H: 3.78 (6H, s, 2OCH_3), 7.00 (4H, d, J = 8.4 Hz, ArH), 7.66 (5H, t, J = 8.4 Hz, ArH), 8.08 (2H, d, J = 7.6 Hz, ArH), 8.42 (3H, d, J = 11.6 Hz, 1ArH and 2-N=CH), 11.89 (2H, s, 2NH); ^{13}C NMR (DMSO-d_6) δ ppm: 55.7, 111.4, 114.8, 121.9, 127.2, 127.2, 128.1, 129.2, 131.0, 134.3, 148.5, 161.3, 162.9; HRMS m/z [M+1]: 431.2674

Bis(2,4-dihydroxybenzylidene)isophthalohydrazide[5] (17)

Yield 85%, R_f = 0.33, m.p. 298-300 °C, (lit 300-302 °C), IR KBr (cm^{-1}): 1607, 1614, 1710, 1765, 3258, 3310; ^1H NMR (DMSO-d_6) δ H: 6.64 (2H, t, J = 7.2 Hz, ArH), 7.52 (2H, s, ArH), 7.70 (3H, t, J = 6.8 Hz, ArH), 8.20 (2H, d, J = 7.2 Hz, ArH), 8.44 (2H, s, 2-N=CH), 8.47 (1H, s, ArH), 9.90 (4H, s, 4OH), 11.98 (2H, s, 2NH); ^{13}C NMR (DMSO-d_6) δ ppm: 103.6, 108.3, 111.4, 124.7, 128.7, 130.1, 132.6, 134.8, 144.6, 160.2, 162.4, 163.8; HRMS m/z [M+1]: 435.4036

Bis(2,4-dimethoxybenzylidene)isophthalohydrazide (18)

Yield 87%, R_f = 0.51, m.p. 230-232 °C, IR KBr (cm^{-1}): 1611, 1624, 1754, 1766, 3245, 3289; ^1H NMR (DMSO-d_6) δ H: 3.72 (12H, s, 4OCH_3), 7.00 (2H, d, J = 7.6 Hz, ArH), 7.19 (2H, d, J = 8.4 Hz, ArH), 7.33 (2H, s, ArH), 7.64 (1H, d, J = 7.2 Hz, ArH), 8.38 (2H, s, 2-N=CH), 8.34 (2H, s, 2-N=CH), 8.41 (1H, s, ArH), 11.91 (2H, s, 2NH); ^{13}C NMR (DMSO-d_6) δ ppm:

56.9, 108.6, 111.9, 122.4, 127.3, 128.3, 129.1, 131.0, 134.4, 148.8, 149.5, 151.3, 162.9; HRMS m/z [M+1]: 491.2958

Bis(3-ethoxy-4-hydroxybenzylidene)isophthalohydrazide (19)

Yield 85%, R_f = 0.45, m.p. 198-200 °C, IR KBr (cm^{-1}): 1610, 1619, 1740, 1756, 3245, 3269, 3510, 3547; ^1H NMR (DMSO-d_6) δ H: 1.33 (6H, t, J = 7.2 Hz, $2CH_3$), 4.03 (4H, q, J = 7.2 Hz, $2OCH_2$), 6.84 (2H, d, J = 8.4 Hz, ArH), 7.07 (2H, d, J = 8.0 Hz, ArH), 7.28 (2H, s, ArH), 7.63 (1H, t, J = 7.6 Hz, ArH), 8.05 (2H, d, J = 8.0 Hz, ArH), 8.33 (2H, s, 2-N=CH), 8.40 (1H, s, ArH), 9.47 (2H, s, 2OH), 11.83 (2H, s, 2NH); ^{13}C NMR (DMSO-d_6) δ ppm: 15.1, 64.3, 110.7, 115.9, 122.6, 126.0, 127.2, 129.1, 131.0, 134.3, 144.6, 147.6, 149.2, 162.8; HRMS m/z [M+1]: 491.2958

Bis(4-hydroxy-3-methoxybenzylidene)isophthalohydrazide (20)

Yield 86%, R_f = 0.43, m.p. 252-254 °C, IR KBr (cm^{-1}): 1611, 1620, 1726, 1768, 3247, 3342, 3517, 3548; ^1H NMR (DMSO-d_6) δ H: 3.81 (6H, s, $2OCH_3$), 6.83 (2H, d, J = 8.0 Hz, ArH), 7.07 (2H, d, J = 8.0 Hz, ArH), 7.30 (2H, s, ArH), 7.64 (1H, t, J = 7.6 Hz, ArH), 8.05 (2H, d, J = 7.6 Hz, ArH), 8.34 (2H, s, 2-N=CH), 8.40 (1H, s, ArH), 9.55 (2H, s, 2OH), 11.83 (2H, s, 2NH); ^{13}C NMR (DMSO-d_6) δ ppm: 56.03, 109.4, 115.9, 112.7, 126.0, 127.2, 129.1, 131.0, 134.3, 148.5, 149.2, 149.5, 162.8; HRMS m/z [M+1]: 463.2538

Bis(2,4-dichlorobenzylidene)isophthalohydrazide (21)

Yield 89%, R_f = 0.53, m.p. 230-232 °C, IR KBr (cm^{-1}): 1613, 1621, 1741, 1752, 3251, 3340; ^1H NMR (DMSO-d_6) δ H: 7.34 (2H, d, J = 6.8 Hz, ArH), 7.72-7.73 (3H, m, ArH), 8.06 (2H, d, J = 8.8 Hz, ArH), 8.28 (2H, d, J = 7.8 Hz, ArH), 8.38 (1H, s, ArH), 8.44 (2H, s, 2-N=CH), 12.10 (2H, s, 2NH); ^{13}C NMR (DMSO-d_6) δ ppm: 125.6, 127.9, 128.1, 128.4, 129.3, 129.9, 130.4, 131.6, 133.5, 134.6, 144.6, 163.5; HRMS m/z [M+]: 508.1203

Bis(2,4-dinitrobenzylidene)isophthalohydrazide (22)

Yield 91%, $R_f = 0.48$, m.p. 244-246 °C, IR KBr (cm^{-1}): 1604, 1614, 1745, 1759, 3247, 3259; ^1H NMR (DMSO-d_6) δ H: 7.55 (2H, d, $J = 6.2$ Hz, ArH), 8.20 (2H, d, $J = 7.8$ Hz, ArH), 8.36 (2H, d, $J = 7.2$ Hz, ArH), 8.42 (2H, s, 2-N=CH), 8.48 (1H, s, ArH), 8.55 (3H, t, $J = 7.8$ Hz, ArH), 12.19 (2H, s, 2NH); ^{13}C NMR (DMSO-d_6) δ ppm: 120.3, 124.5, 129.3, 130.1, 131.2, 132.6, 134.5, 137.9, 144.6, 149.3, 152.6, 163.8; HRMS m/z [M+1]: 551.3960

Bis(2,4-difluorobenzylidene)isophthalohydrazide (23)

Yield 88%, $R_f = 0.52$, m.p. 278-280 °C, IR KBr (cm^{-1}): 1608, 1624, 1710, 1756, 3265, 3342; ^1H NMR (DMSO-d_6) δ H: 7.38 (2H, t, $J = 8.0$ Hz, ArH), 7.33 (2H, q, $J = 9.2$ Hz, ArH), 7.68 (1H, t, $J = 7.6$ Hz, ArH), 7.99 (2H, q, $J = 8.0$ Hz, ArH), 8.11 (2H, d, $J = 7.2$ Hz, ArH), 8.47 (1H, s, ArH), 8.65 (2H, s, 2-N=CH), 12.13 (2H, s, 2NH); ^{13}C NMR (DMSO-d_6) δ ppm: 104.9, 112.9, 119.0, 127.3, 128.4, 129.3, 131.3, 133.9, 140.5, 160.1, 162.9, 164.9; HRMS m/z [M+1]: 443.3651

Bis(2,4-dibromobenzylidene)isophthalohydrazide (24)

Yield 88%, $R_f = 0.56$, m.p. 220-222 °C, IR KBr (cm^{-1}): 1609, 1615, 1742, 1769, 3257, 3345; ^1H NMR (DMSO-d_6) δ H: 7.53 (2H, d, $J = 8.2$ Hz, ArH), 7.63 (2H, t, $J = 6.2$ Hz, ArH), 7.68 (1H, t, $J = 7.6$ Hz, ArH), 8.03 (2H, s, ArH), 8.25 (2H, d, $J = 6.8$ Hz, ArH), 8.40 (1H, s, ArH), 8.46 (2H, s, 2-N=CH), 12.10 (2H, s, 2NH); ^{13}C NMR (DMSO-d_6) δ ppm: 122.6, 124.9, 125.3, 128.3, 130.1, 131.3, 133.4, 134.6, 135.1, 136.5, 146.3, 163.8; HRMS m/z [M+]: 688.9890

Bis(3,4,5-trihydroxybenzylidene)isophthalohydrazide[6] (25)

Yield 84%, $R_f = 0.30$, m.p. 266-268 °C, IR KBr (cm^{-1}): 1610, 1618, 1742, 1768, 3241, 3278, 3542, 3548, 3589; ^1H NMR (DMSO-d_6) δ H: 6.86 (4H, s, ArH), 7.73 (1H, t, $J = 6.8$ Hz, ArH), 8.23 (2H, d, $J = 8.8$ Hz, ArH), 8.44 (1H, s, ArH), 8.48 (2H, s, 2-N=CH), 9.88 (6H, s, 6OH), 12.13 (2H, s, 2NH); ^{13}C NMR (DMSO-d_6) δ ppm: 109.3, 125.6, 128.3, 129.7, 131.6, 134.9, 139.6, 146.8, 148.7, 163.2; HRMS m/z [M+1]: 467.4102

Bis(3,4,5-trimethoxybenzylidene)isophthalohydrazide (26)

Yield 87%, R_f = 0.49, m.p. 250-252 °C, IR KBr (cm^{-1}): 1608, 1617, 1712, 1759, 3217, 3258; ^{1}H NMR (DMSO-d_6) δ H: 1.37 (6H, s, 2OCH_3), 3.83 (12H, s, 4OCH_3), 7.02 (4H, s, ArH), 7.67 (1H, d, J = 8.0 Hz, ArH), 7.70 (2H, s, ArH), 8.39 (1H, s, ArH), 8.41 (2H, s, 2-N=CH), 11.98 (2H, s, 2NH); ^{13}C NMR (DMSO-d_6) δ ppm: 56.4, 60.5, 104.8, 127.3, 129.2, 130.1, 131.1, 134.2, 139.9, 148.7, 153.6, 163.0; HRMS m/z [M+1]: 551.2360

Dipropylideneisophthalohydrazide (27)

Yield 91%, R_f = 0.54, m.p. 268-270 °C, IR KBr (cm^{-1}): 1612, 1627, 1752, 1760, 3247, 3269; ^{1}H NMR (DMSO-d_6) δ H: 1.10 (6H, t, J = 6.8 Hz, $(CH_3)_2$), 2.24 (4H, m, 2CH_2), 7.51 (1H, t, J = 7.2 Hz, ArH), 7.84 (2H, d, J= 7.8 Hz, ArH), 8.20 (1H, s, ArH), 8.41 (2H, s, 2-N=CH), 12.09 (2H, s, 2NH); ^{13}C NMR (DMSO-d_6) δ ppm:10.5, 25.9, 124.6, 128.4, 131.1, 134.3, 149.6, 163.5; HRMS m/z [M+1]: 275.3153

Bis(3-methylbutylidene)isophthalohydrazide (28)

Yield 90%, R_f = 0.52, m.p. 242-244 °C, IR KBr (cm^{-1}): 1612, 1621, 1740, 1766, 3240, 3259; ^{1}H NMR (DMSO-d_6) δ H: 0.90 (12H, d, J = 7.8 Hz, $(CH_3)_4$), 1.52 (4H, t, J = 8.2 Hz, 2CH_2), 1.80 (2H, m, 2CH), 7.62 (1H, t, J = 7.8 Hz, ArH), 7.88 (2H, d, J= 6.8 Hz, ArH), 8.33 (2H, s, 2-N=CH), 8.43 (1H, s, ArH), 12.12 (2H, s, 2NH); ^{13}C NMR (DMSO-d_6) δ ppm: 23.4, 26.4, 33.7, 125.3, 128.4, 131.4, 134.6, 151.1, 163.4; HRMS m/z [M+1]: 331.4286

Diheptylideneisophthalohydrazide (29)

Yield 89%, R_f = 0.55, m.p. 248-249 °C, IR KBr (cm^{-1}): 1610, 1616, 1742, 1770, 3243, 3256; ^{1}H NMR (DMSO-d_6) δ H: 0.91 (6H, t, J = 6.8 Hz, $(CH_3)_2$), 1.28-1.32 (16H, m, $(CH_2)_8$), 1.55 (4H, m, 2CH_2), 7.66 (1H, t, J = 7.2 Hz, ArH), 7.90 (2H, d, J= 7.8 Hz, ArH), 8.36 (2H, s, 2-N=CH), 8.44 (1H, s, ArH), 12.17 (2H, s, 2NH); ^{13}C NMR (DMSO-d_6) δ ppm: 14.2, 22.6, 26.1, 26.8, 29.5, 31.6, 124.6, 128.4, 131.5, 134.7, 148.6, 163.4; HRMS m/z [M+1]: 387.5362

Bis(pyridine-3-ylmethylene)isophthalohydrazide (30)

Yield 88%, R_f = 0.47, m.p. 210-212 °C, IR KBr (cm^{-1}): 1610, 1627, 1728, 1762, 3247, 3268; ^1H NMR (DMSO-d_6) δ H: 7.47 (2H, d, J = 4.8 Hz, ArH), 7.69 (1H, d, J = 6.8 Hz, ArH), 8.14 (4H, t, J = 8.4 Hz, ArH), 8.47 (3H, s, ArH), 8.60 (2H, s, 2-N=CH), 8.86 (2H, s, ArH), 12.20 (2H, s, 2NH); ^{13}C NMR (DMSO-d_6) δ ppm: 123.1, 124.4, 127.5, 129.3, 130.6, 131.4, 134.0, 145.9, 149.2, 151.2, 163.1; HRMS m/z [M+1]: 373.3856

Bis(furan-2-ylmethylene)isophthalohydrazide (31)

Yield 89%, R_f = 0.51, m.p. 287-289 °C, IR KBr (cm^{-1}): 1604, 1612, 1712, 1762, 3241, 3266; ^1H NMR (DMSO-d_6) δ H: 6.60 (2H, t, J = 7.2 Hz, ArH), 7.94 (2H, d, J = 8.8 Hz, ArH), 7.72-7.73 (2H, m, ArH), 8.30 (2H, d, J = 8.2 Hz, ArH), 8.38 (1H, s, ArH), 5.48 (1H, m, ArH), 8.51 (2H, s, 2-N=CH), 11.87 (2H, s, 2NH); ^{13}C NMR (DMSO-d_6) δ ppm: 112.6, 119.3, 125.7, 128.3, 130.4, 134.6, 146.9, 148.3, 151.3, 163.7; HRMS m/z [M+1]: 351.3415

Bis(thiophen-2-ylmethylene)isophthalohydrazide (32)

Yield 88%, R_f = 0.56, m.p. 232-234 °C, IR KBr (cm^{-1}): 1606, 1612, 1742, 1763, 3247, 3268; ^1H NMR (DMSO-d_6) δ H: 7.13 (2H, s, ArH), 7.47 (2H, s, ArH), 7.67 (3H, s, ArH), 8.08 (2H, d, J = 6.8 Hz, ArH), 8.40 (1H, s, ArH), 8.67 (2H, s, 2-N=CH), 11.99 (2H, s, 2NH); ^{13}C NMR (DMSO-d_6) δ ppm: 127.3, 128.3, 129.2, 129.5, 131.1, 131.6, 134.1, 139.4, 143.7, 162.8; HRMS m/z [M+1]: 383.2364

Bis(1H-pyrrole-2-ylmethylene)isophthalohydrazide (33)

Yield 87%, R_f = 0.50, m.p. 263-265 °C, IR KBr (cm^{-1}): 1612, 1618, 1743, 1768, 3256, 3317; ^1H NMR (DMSO-d_6) δ H: 6.40 (2H, t, J = 7.0 Hz, ArH), 6.47 (2H, d, J = 8.6 Hz, ArH), 7.01 (2H, d, J = 6.8 Hz, ArH), 8.71 (1H, t, J = 8.2Hz, ArH), 8.25 (2H, t, J = 7.8 Hz, ArH), 8.45 (2H, s, 2-N=CH), 8.47 (1H, s, ArH), 10.12 (2H, s, 2NH), 12.19 (2H, s, 2NH); ^{13}C NMR (DMSO-d_6) δ ppm: 110.3, 120.4, 124.3, 125.6, 128.7, 130.4, 133.4, 135.7, 147.3, 163.7; HRMS m/z [M+1]: 349.3562

Bis(2-bromo-4-hydroxybenzylidene)isophthalohydrazide (34)

Yield 84%, R_f = 0.39, m.p. 234-236 °C, IR KBr (cm^{-1}): 1612, 1621, 1742, 1766, 3217, 3269, 3510, 3562; ^1H NMR (DMSO-d_6) δ H: 7.67 (2H, t, J = 8.0 Hz, ArH), 7.91 (4H, s, ArH), 8.08 (3H, t, J = 8.0 Hz, ArH), 8.31 (2H, s, 2-N=CH), 8.41 (1H, s, ArH), 10.47 (2H, s, 2OH), 12.11 (2H, s, 2NH); ^{13}C NMR (DMSO-d_6) δ ppm: 112.6, 115.1, 123.5, 127.4, 129.2, 130.6, 131.1, 131.2, 134.0, 145.6, 152.6, 163.0; HRMS m/z [M+1]: 561.1953

Bis(2-bromo-4-methoxybenzylidene)isophthalohydrazide (35)

Yield 86%, R_f = 0.53, m.p. 226-228 °C, IR KBr (cm^{-1}): 1612, 1619, 1742, 1768, 3214, 3228; ^1H NMR (DMSO-d_6) δ H: 3.82 (6H, s, 2OCH_3), 7.01 (2H, d, J = 8.0 Hz, ArH), 7.67-7.68 (4H, m, ArH), 7.71 (1H, t, J = 6.8 Hz, ArH), 8.21 (2H, t, J = 7.0 Hz, ArH), 8.37 (2H, s, 2-N=CH), 8.43 (1H, s, ArH), 12.19 (2H, s, 2NH); ^{13}C NMR (DMSO-d_6) δ ppm: 55.3, 114.0, 120.4, 122.4, 125.3, 127.3, 128.3, 130.4, 132.6, 134.8, 146.9, 160.7, 163.6; HRMS m/z [M+1]: 589.3245

Bis(5-bromo-4-hydroxy-3-methoxybenzylidene)isophthalohydrazide (36)

Yield 85%, R_f = 0.45, m.p. 256-258 °C, IR KBr (cm^{-1}): 1610, 1620, 1742, 1768, 3219, 3269, 3542, 3562; ^1H NMR (DMSO-d_6) δ H: 3.88 (6H, s, 2OCH_3), 7.32 (2H, s, ArH), 7.39 (2H, s, ArH), 7.66 (1H, m, ArH), 8.07(2H, d, J = 7.2 Hz, ArH), 8.32 (2H, s, 2-N=CH), 8.40 (1H, s, ArH), 9.98 (2H, s, 2OH), 11.95 (2H, s, 2NH); ^{13}C NMR (DMSO-d_6) δ ppm: 56.7, 108.7, 109.8, 124.9, 126.9, 127.3, 129.2, 131.1, 134.2, 146.3, 147.6, 149.1, 162.9; HRMS m/z [M+1]: 621.4521

Biology

Antimicrobial activity

Bacterial strains

All reference bacterial strains were obtained from Microbial Typing Culture Collection (MTCC), Chandigarh. *Salmonella typhimurium* (98), *Escherichia coli* (1610), *Staphylococcus aureus* (96), *Bacillus cereus* (430), *Shigella flexneri* (1457), and

Enterobacter aerogenes (13048) strains were cultured as per the protocol prescribed by MTCC.

Antibiotic agar dilution method

The bacterial strains were subjected to agar dilution method according to the reported method [7] to deduce the compounds susceptibility. Along with the reference bacterial strains received from Microbial Typing Culture Collection (MTCC), Chandigarh, India, as a positive controls. The bacterial suspensions were prepared from the overnight culture and $1x10^6$ CFU/mL cells were inoculated on to Nutrient agar, then plates were bored using cork borer (7 mm) to create wells, to which 10 µL of different serial dilutions of compounds were added. Control was performed without any test sample and incubated at 37 ^oC for 24 h to examine zone of inhibition. Assay performed in triplicates and repeated thrice.

Micro dilution based metods

The assay was performed in 96 well plates containing 100 µL of bacterial broth culture, 10 µL of varying concentrations (10 to 45 µg/mL) of different test compounds in each well according to the reported method [8] and plates were incubated for 6 h at 37 °C. After incubation, 20 µL of 10 mg/mL triphenyl tetrazolium chloride (TTC) in phosphate buffer was added to each well and incubated for 1 h. Formation of blue color is the indication for viable bacteria and visual MIC break-point was determined for respective test compounds. Control culture was carried out without test samples [7] MIC defined as the lowest concentration at which no visible growth was observed as per Clinical and Laboratory Standards Institute (CLSI). Assay performed in triplicates and repeated thrice.

Action of compounds on Shigella flexneri biofilm growth

Inoculum preparation

The *S. flexneri* was grown on nutrient agar at 35 °C for 18-20 h and cells were harvested by centrifuging at 5000 rpm for 8 min at 4 °C. Wash the cells thrice in sterile saline

solution and re-suspend pelleted cells in saline solution. Cell density adjusted to an optical density at 600 nm (OD600) of 0.1 using a UV-visible spectrometer and viable counts of approximately 6 log CFU/mL.

Anti-biofilm activity of compounds

The qualitative and quantitative test was performed for determination of biofilm production using microtiter plate method (MtP). The experiment was set according to the reported method [9] with slight modifications, 20 µl aliquots of cell suspension were inoculated into each one of six well polystyrene microtiter plate containing 180 µl of nutrient agar supplemented with glucose (10 g/100 mL). MtP was covered and incubated at the static condition of 37 °C for 18 h to favors greater adherence *S. flexneri*. After, each well was washed thrice with saline solution; cells were fixed with 150 µl of methanol for 20 min and dry the MtP at room temperature. The cells were stained with crystal violet (0.5%) for 15 min then discard the contents and wash thrice with 200 µl of saline solution. Dry the MtP, using 150 µl of 95% ethanol then dye bound to the cells was eluted for 30 min and the absorbance at 490 nm was determined using microplate spectrophotometer.

Release of cellular material

Effects of compounds were analyzed by measuring cellular material (DNA) from *S. flexneri* as a model organism and followed according to the repotted method [10]. The experiment was carried out by inoculating log phase culture into 0.1% sterile peptone water and without samples as a control. After incubation at 37 ^{o}C, 1 mL of broth was transferred to an Eppendorf tube, centrifuged at 3,500 rpm and the supernatant was measured at 260 nm using a spectrophotometer. Results were expressed in the form of optical density for the sample collected at different time intervals (for 0, 30, 60, 120 and 150 min) incubated samples. Assay performed in triplicates and repeated thrice.

Molecular docking studies

Molecular docking protocol was followed according to the reported method [11]. Briefly, The structural drawing and geometry cleaning of novel potential leads **R (1-36)** was performed in Maestro 9.3 of Schrödinger suite 2012 platform and then subjected to other parameters *viz* energy minimization by using OPLS 2005 force field, addition of hydrogen atoms, neutralization of charged groups, generation of ionization states and set pH 7.5 using Epik. Generation of tautomers and stereoisomers of 32 per ligand and low-energy ring conformations and optimize the geometries followed by generating low energy ring conformation per ligand was computed, optimized by LigPrep and used for molecular docking.

The co-ordinates of DNA Gyrase from *Staphylococcus aureus* complex with Ciprofloxacin and DNA and MurB from *E coli* were obtained from the Brookhaven Protein Data Bank, whose PDB ids are 2XCT [12] and 1MBT [13] respectively. Crystal structure was imported and refined by a multistep process through the protein preparation wizard of Maestro 9.3, which includes energy minimization using OPLS-2005 force field, correct bond orders were assigned, hydrogen atoms were added and the water molecules where removed beyond 5Å from hetero atom, formal charges, amide groups of Asn and Gln were optimized. All amino acid flips were assigned to correct geometry and hydrogen bonds were optimized. Using PROPKA, pH was fixed and optimized to 7.5. Non-hydrogen atoms were minimized by restrained minimization to default RMSD to 0.3Å. Using Extra-precision (XP) docking and scoring each compound were docking into the receptor grid of radii 20Å × 20Å × 20Å and validation of the best docking calculation were judge based on the Glide score as compared with standard antibiotic (Ampicillin).

References:

1. M. Akbar, F. Naser, R. Abdolhossein, *Heterocycl. Commun*., 2013, **19**, 265-269.
2. Y. Zhang, A. Wang, C. Cao, Y. Leng, T. Wei, *Chinese. J. Chem*., 2009, **27**, 1617-1623.
3. H. Weiwei, Y. Xudong, L. Hai, L. Huakuan, *J. Incl. Phenom. Macrocycl. Chem*., 2011, **69**, 69-73.
4. S. Sanyog, S. H. Maninder, W. Amandeep, V. Vanita, H. Geeta, *Org. Biomol. Chem*., 2014, **12**, 4445-4453.
5. Y. Zhang, A. Wang, C. Cao, Y. Leng, T. Wei, *Chinese. J. Chem*., 2009, **27**, 1617-1623.
6. I. Rafiqul, K. Fumiaki, K. Yasuo, I. Kengo, O. Tadashi, M. Mitsuko, *Bioorg. Med. Chem. Lett*., 2014, **24**, 3802-3806.
7. H. M. Manukumar, S. Umesha, *Scientific Reports*. 2017, *7*.
8. J. Qiu, D. Wang, H. Xiang, H. Feng, Y. Jiang, L. Xia et al., *PLoS One*. 2010, **5**, e9736.
9. J. B. Dos Santos Rodrigues, R. J. De Carvalho, N. T. De Souza, K. De Sousa Oliveira, O. L. Franco, D. Schaffner, M. Magnani, *Food Control*. 2017, **73**, 1237-1246.
10. A. K. Chauhan, S. C. Kang, *Reser. Microbiolo*., 2014, **165**, 559-565.
11. H. K. Vivek, J. R. Kumar, B. S. Priya, S. Nanjunda Swamy, *Mol. Cell. Biochem*., 2017, DOI 10.1007/s11010-016-2888-6
12. K. Raghavendra, N. Renuka, H. K. Vivek, S. Bharath, A. Ajaykumar, S. Shashikanth, *Bioorg. Med. Chem. Lett*., 2016, **26**, 3621-3625.
13. M. P. Mehul, J. P. Laxman, *Sci. World. J*., 2014, Article ID 897187, 1-10.

^{1}H, ^{13}C NMR and Mass spectrum of representative compounds

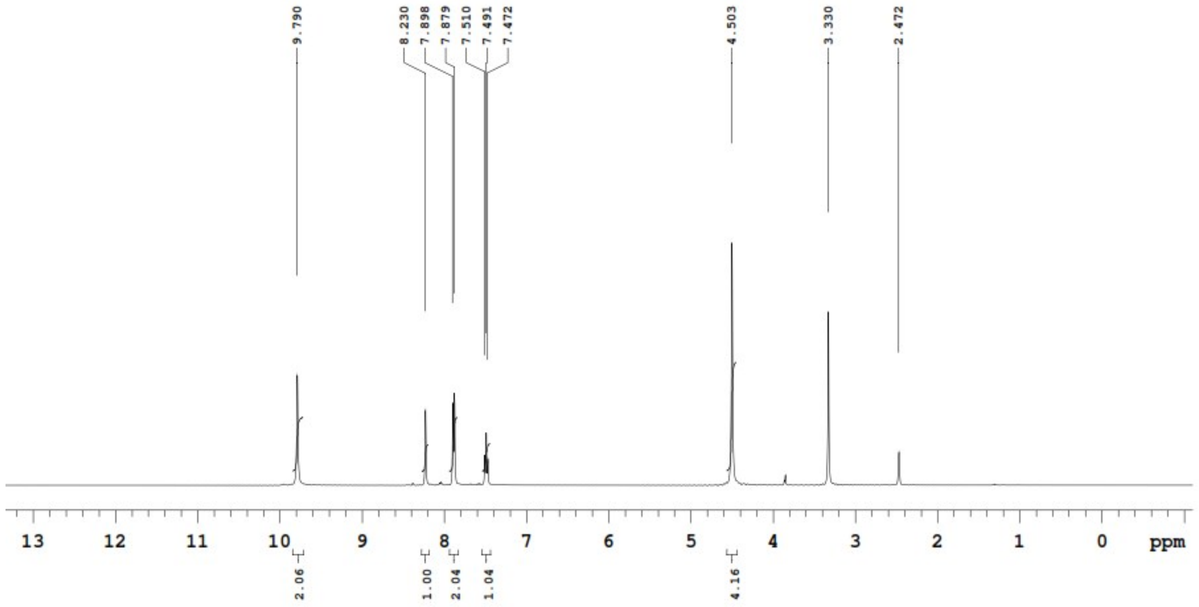

^{1}H NMR spectra of compound no 3

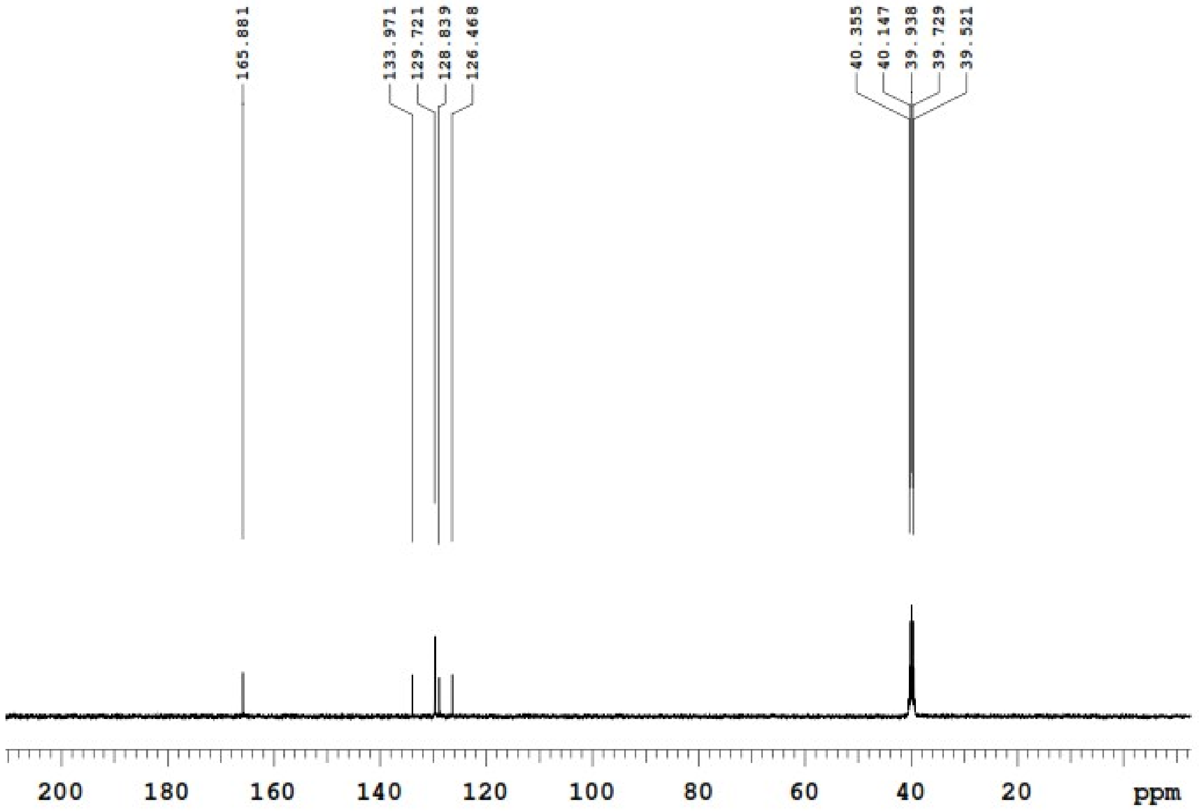

^{13}C NMR spectra of compound no 3

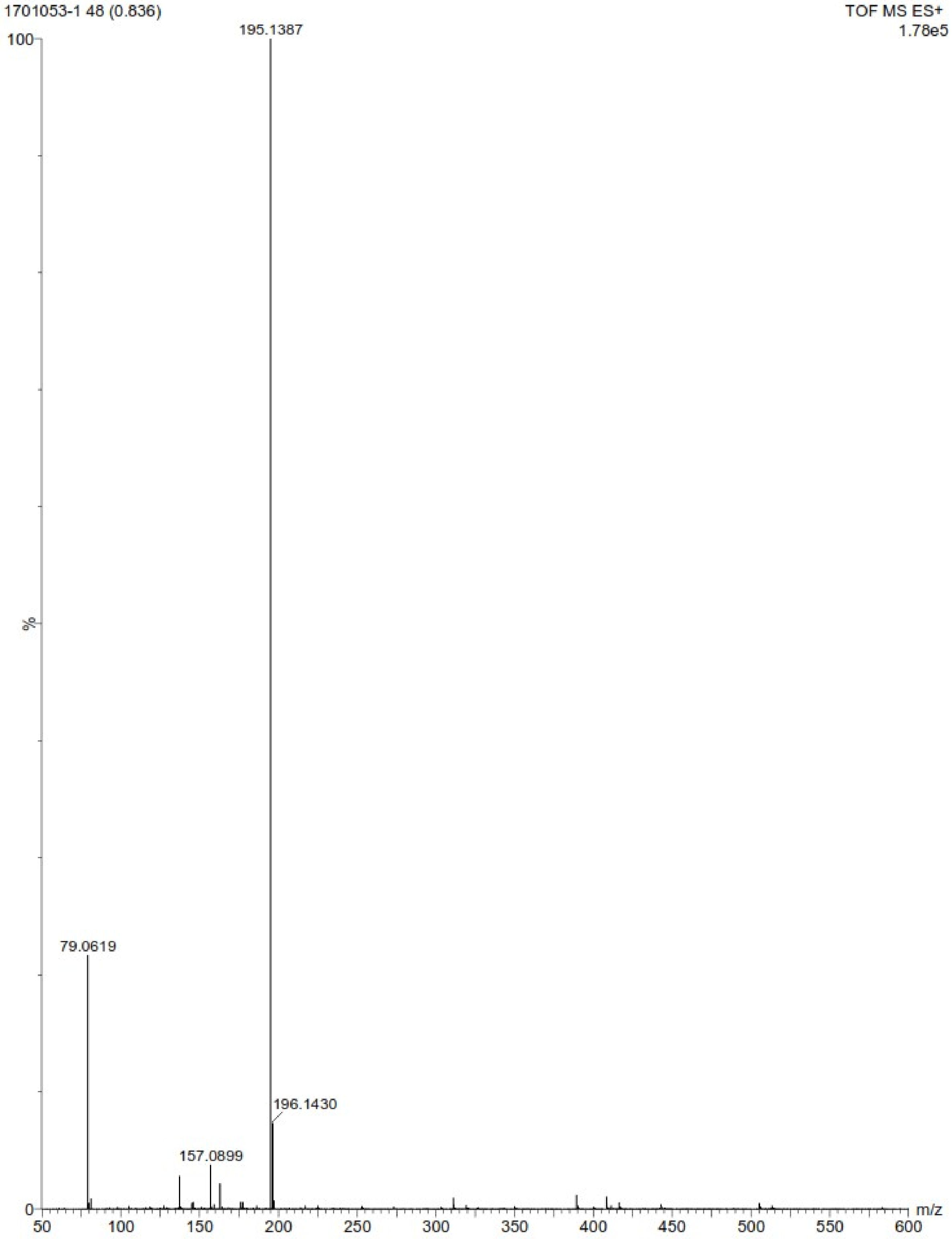

Mass spectra of compound no 3

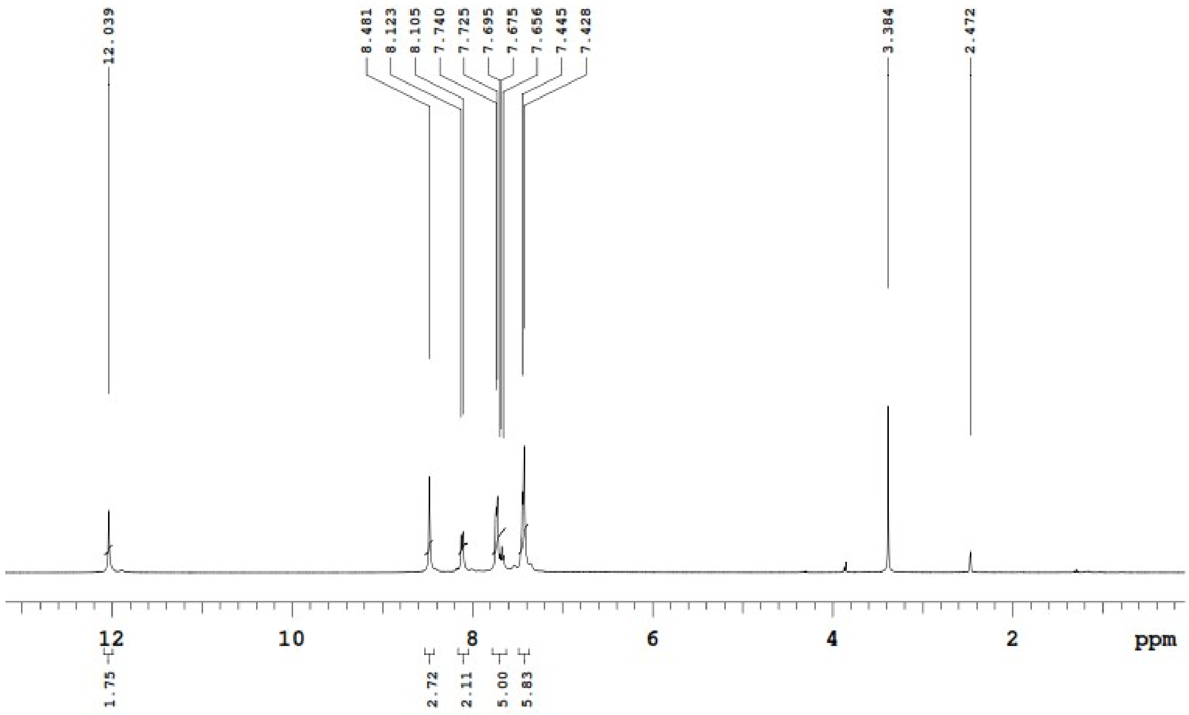

^{1}H NMR spectra of compound no 4

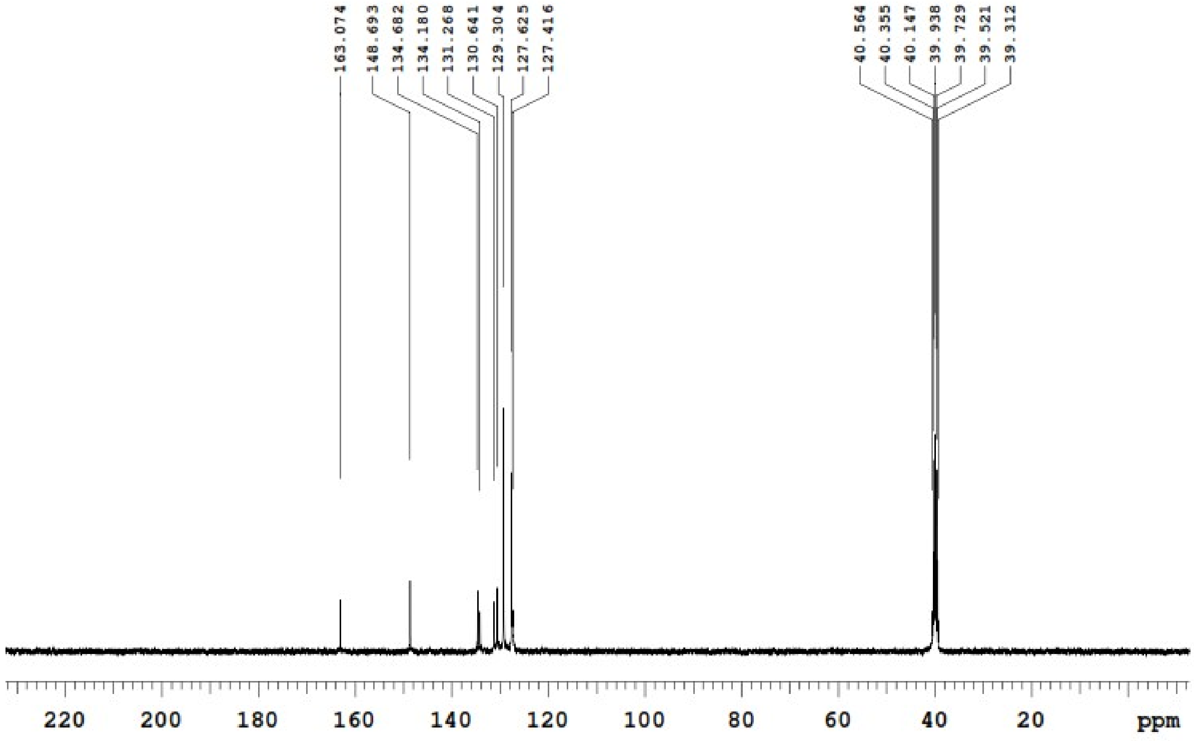

^{13}C NMR spectra of compound no 4

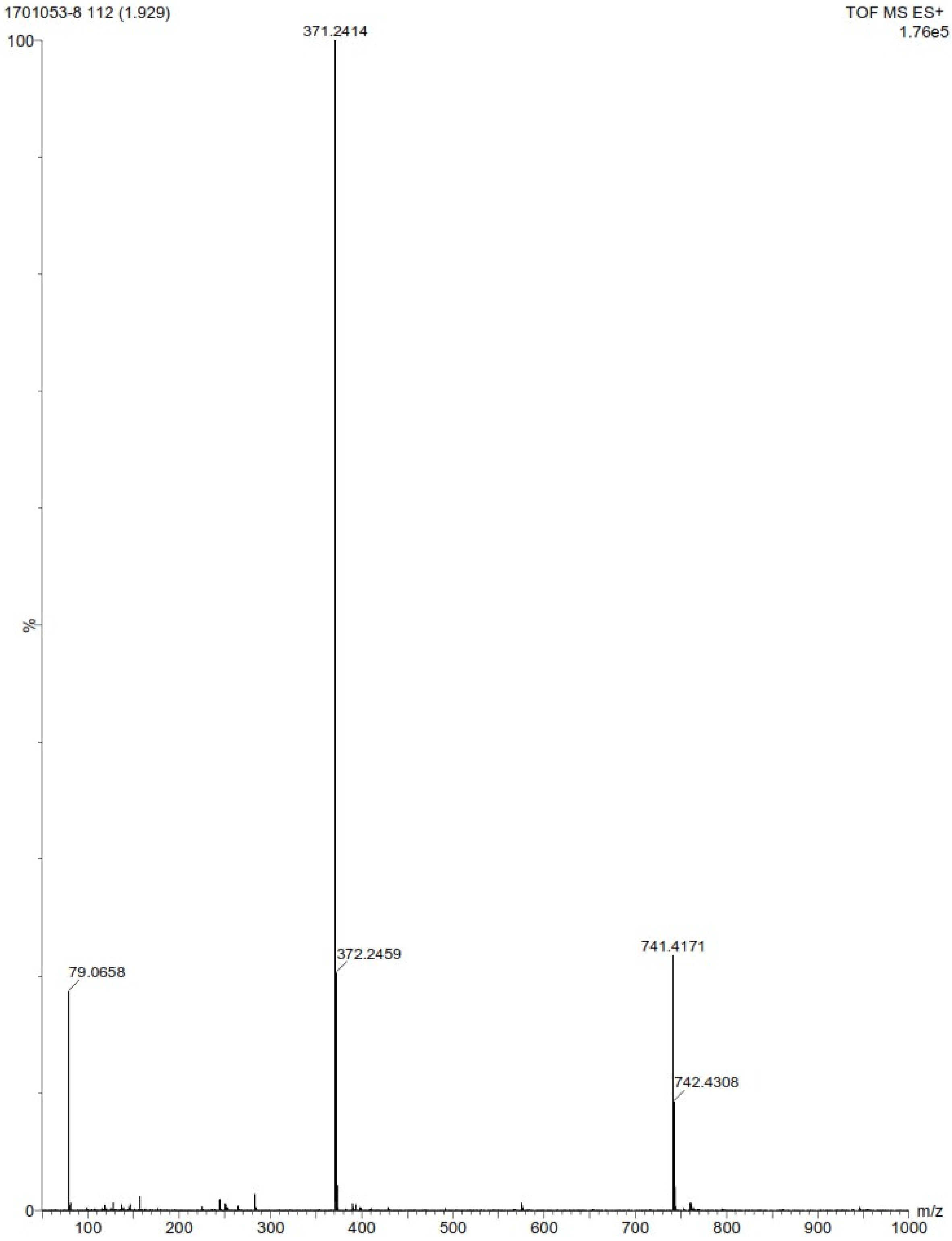

Mass spectra of compound no 4

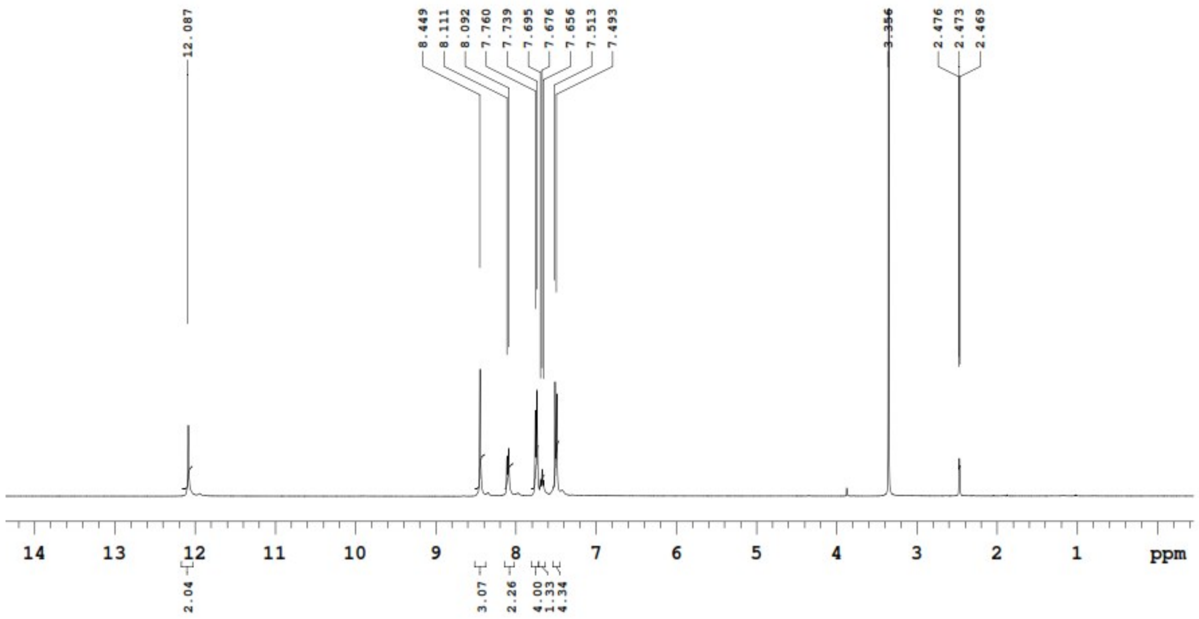

^{1}H NMR spectra of compound no 5

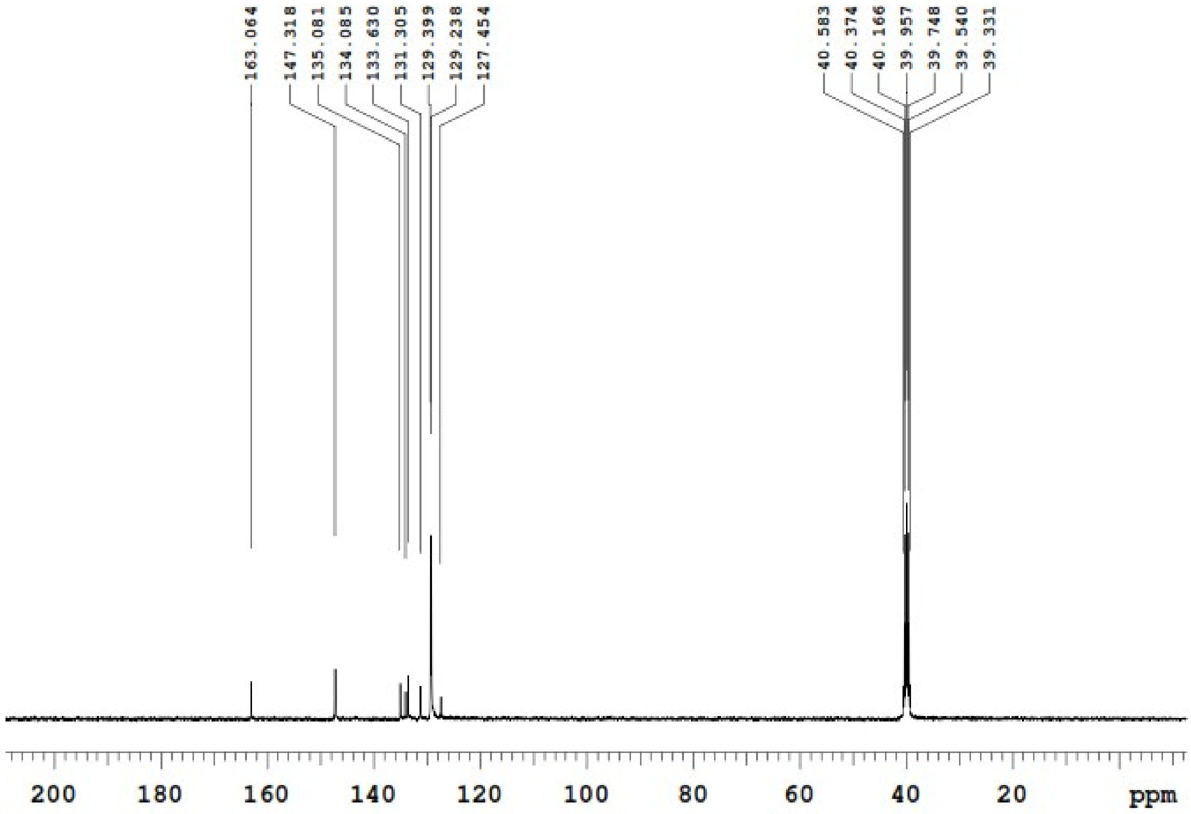

^{13}C NMR spectra of compound no 5

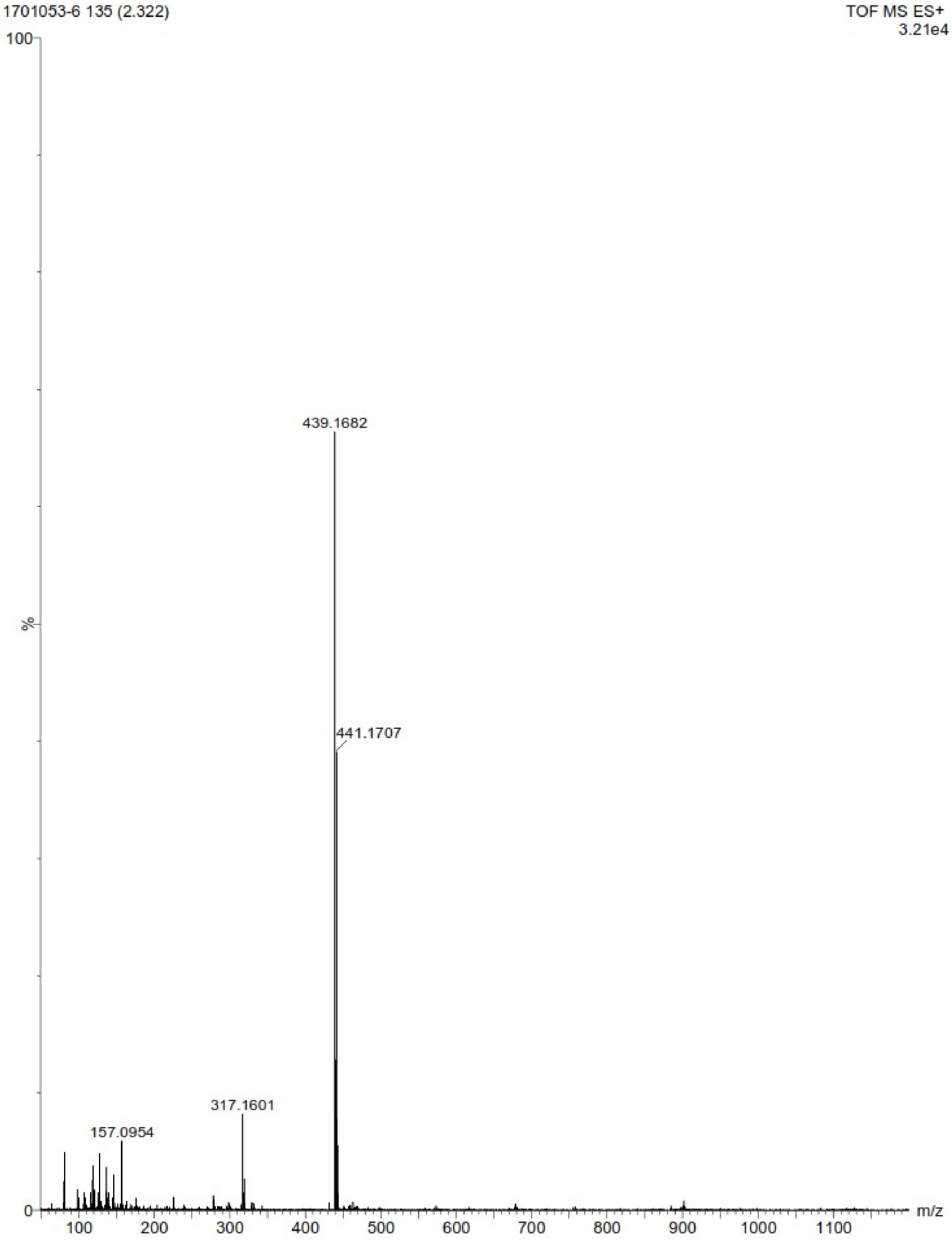

Mass spectra of compound no 5

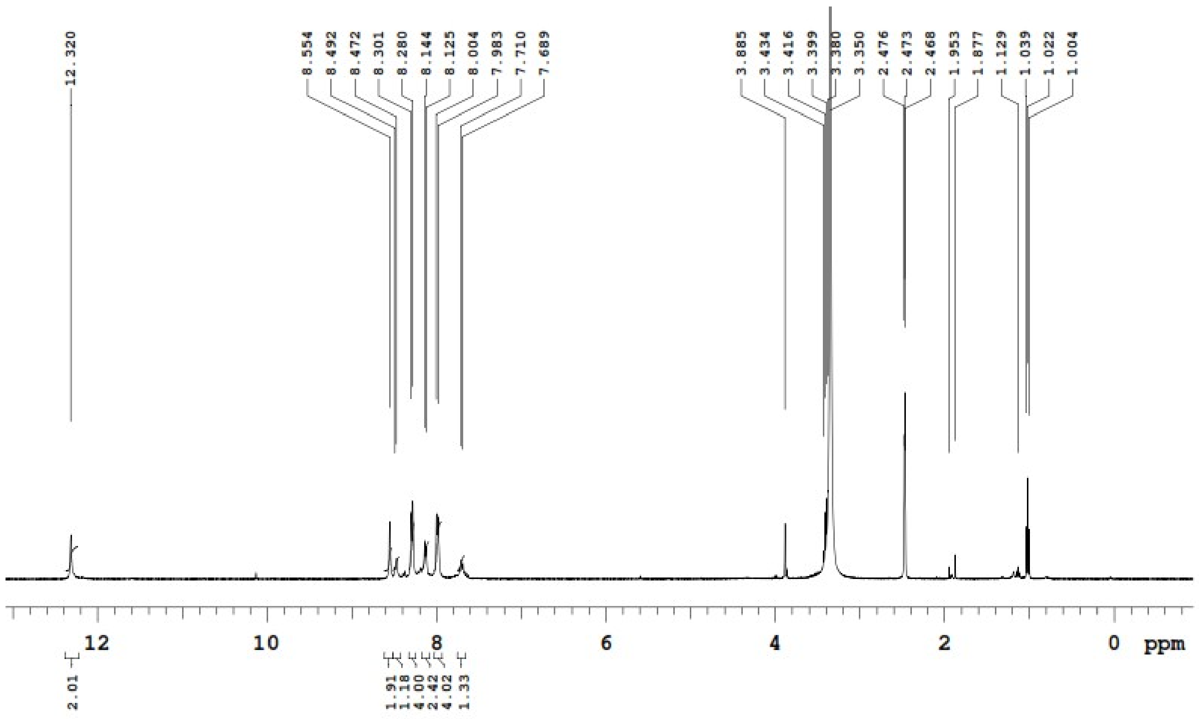

^{1}H **NMR spectra of compound no 6**

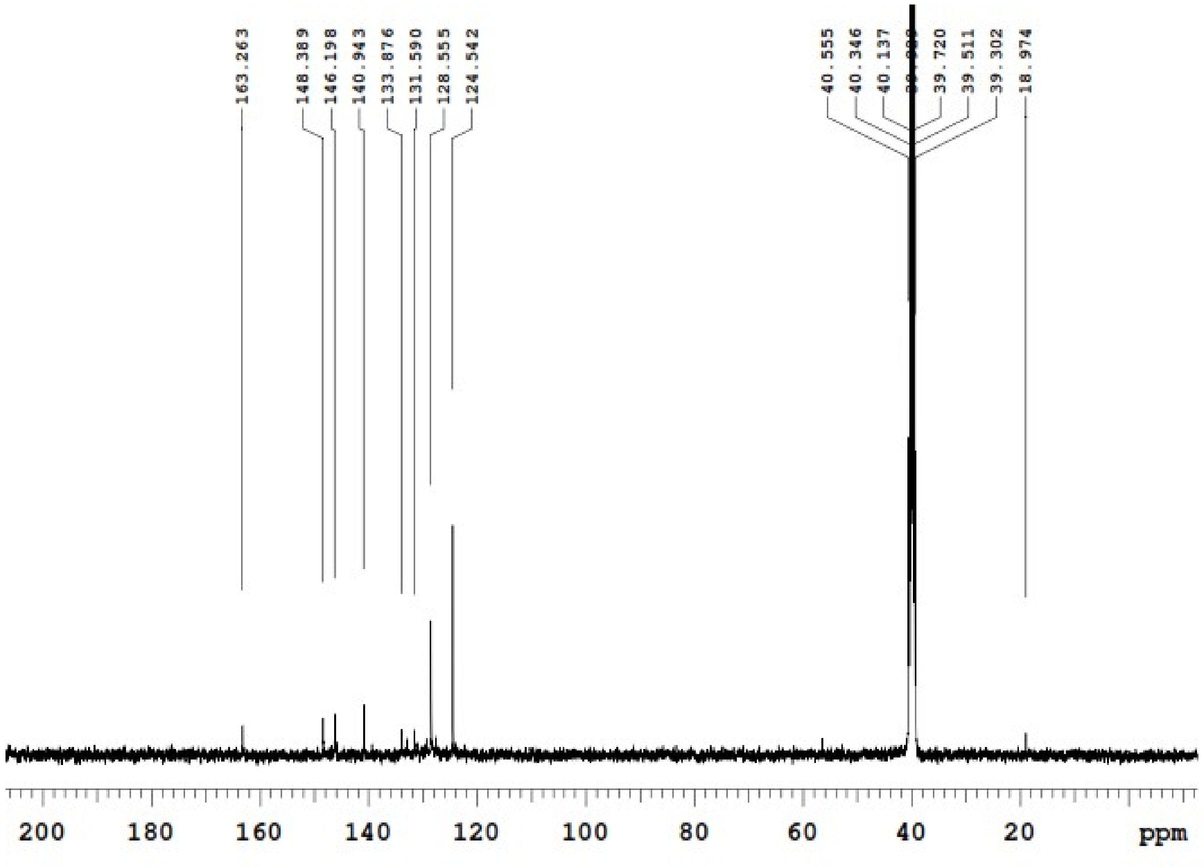

^{13}C **NMR spectra of compound no 6**

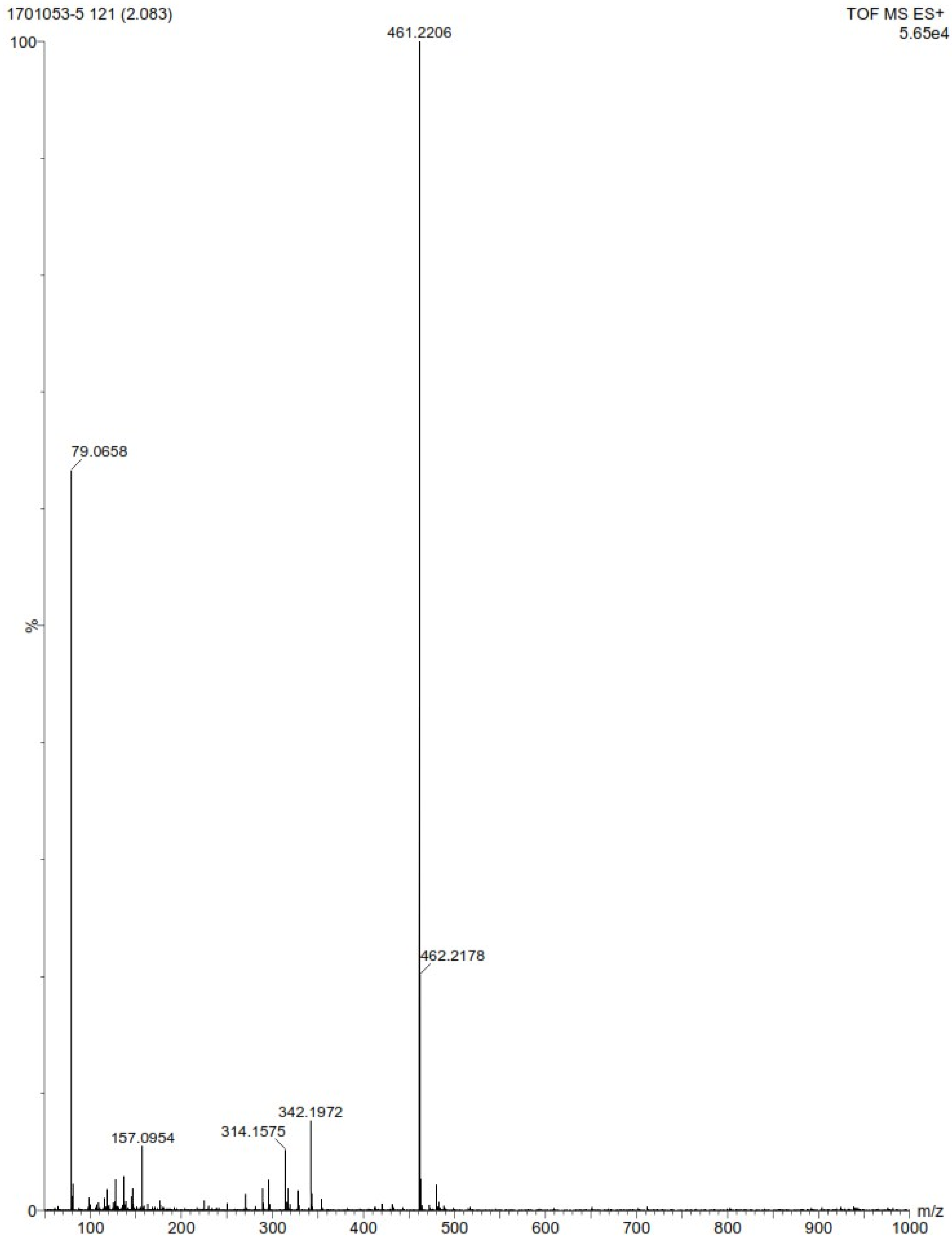

Mass spectra of compound no 6

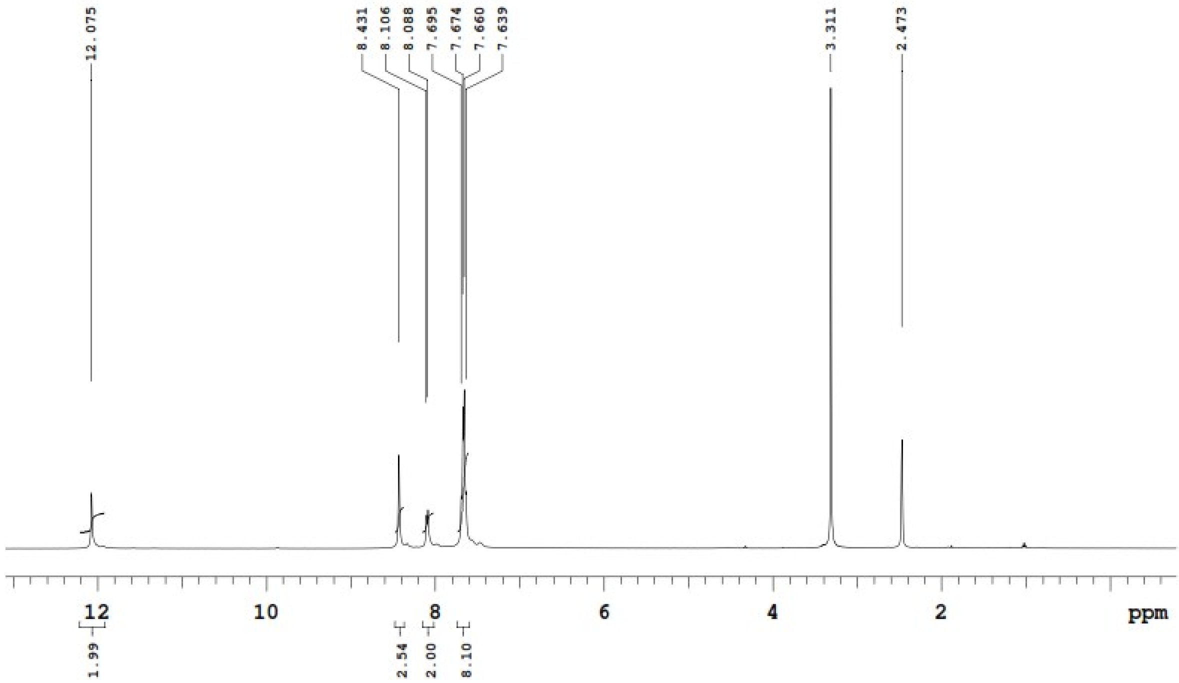

^{1}H NMR spectra of compound no 8

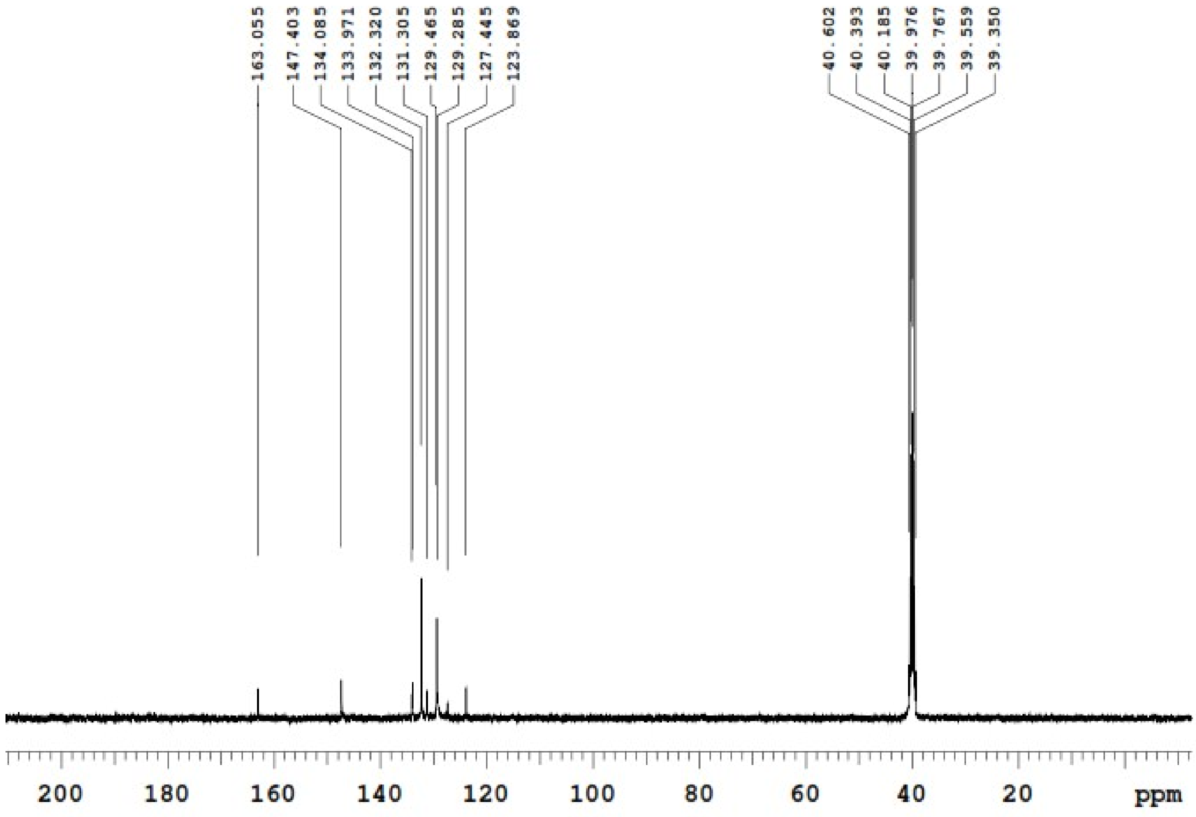

^{13}C NMR spectra of compound no 8

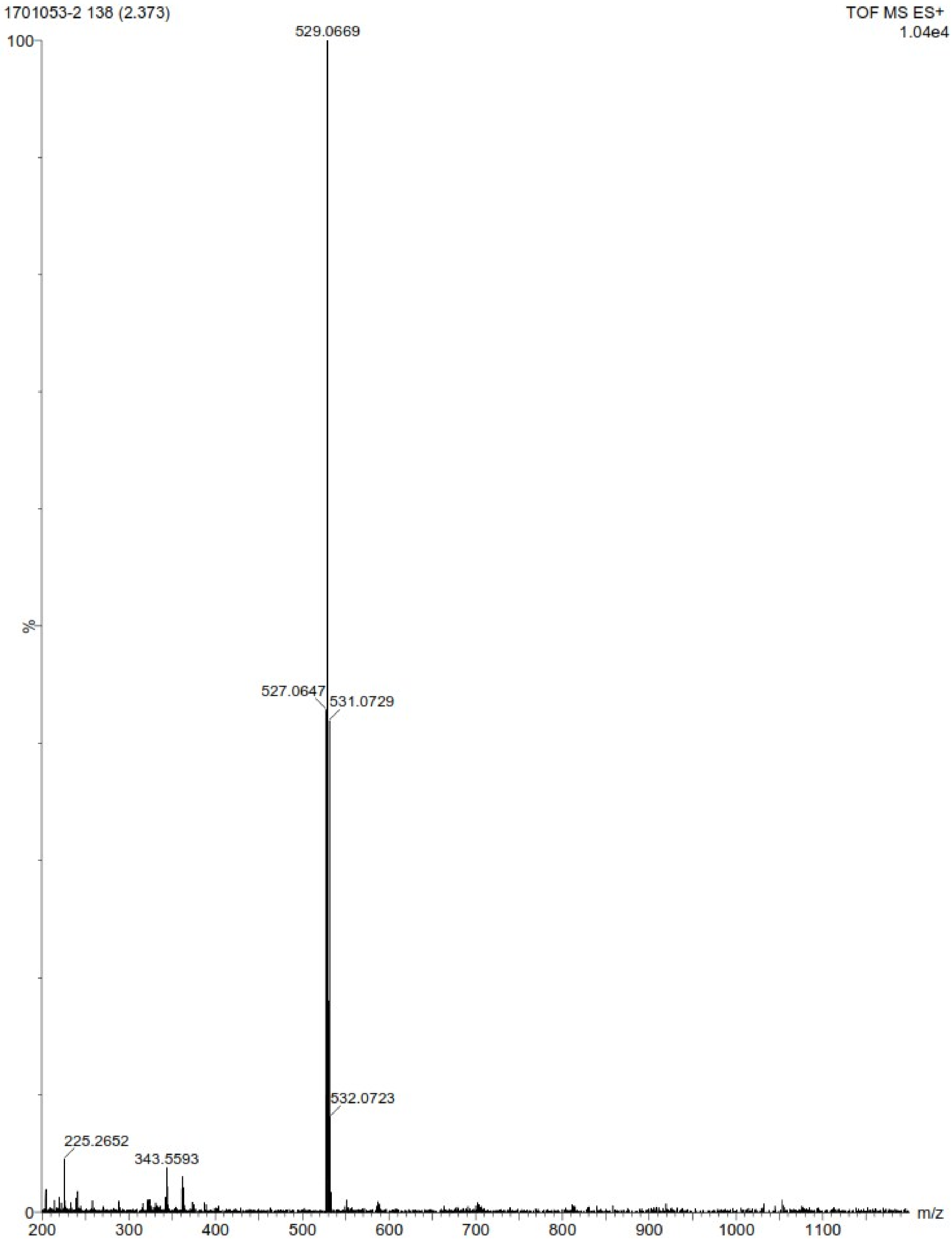

Mass spectra of compound no 8

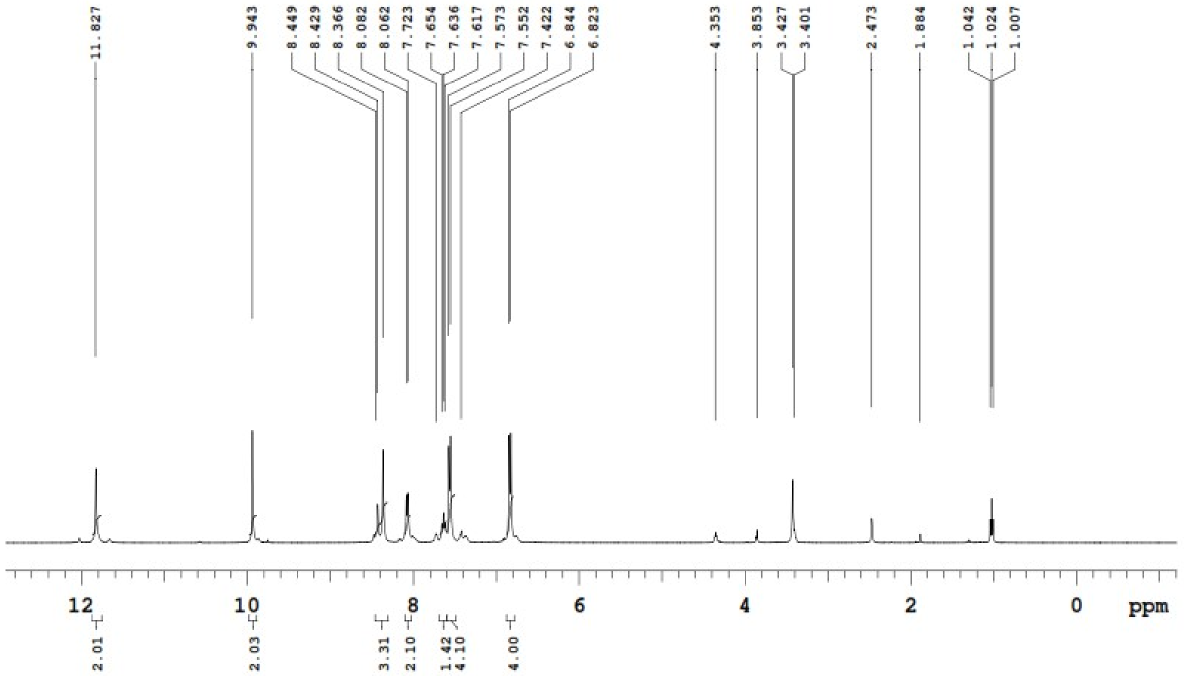

^{1}H NMR spectra of compound no 9

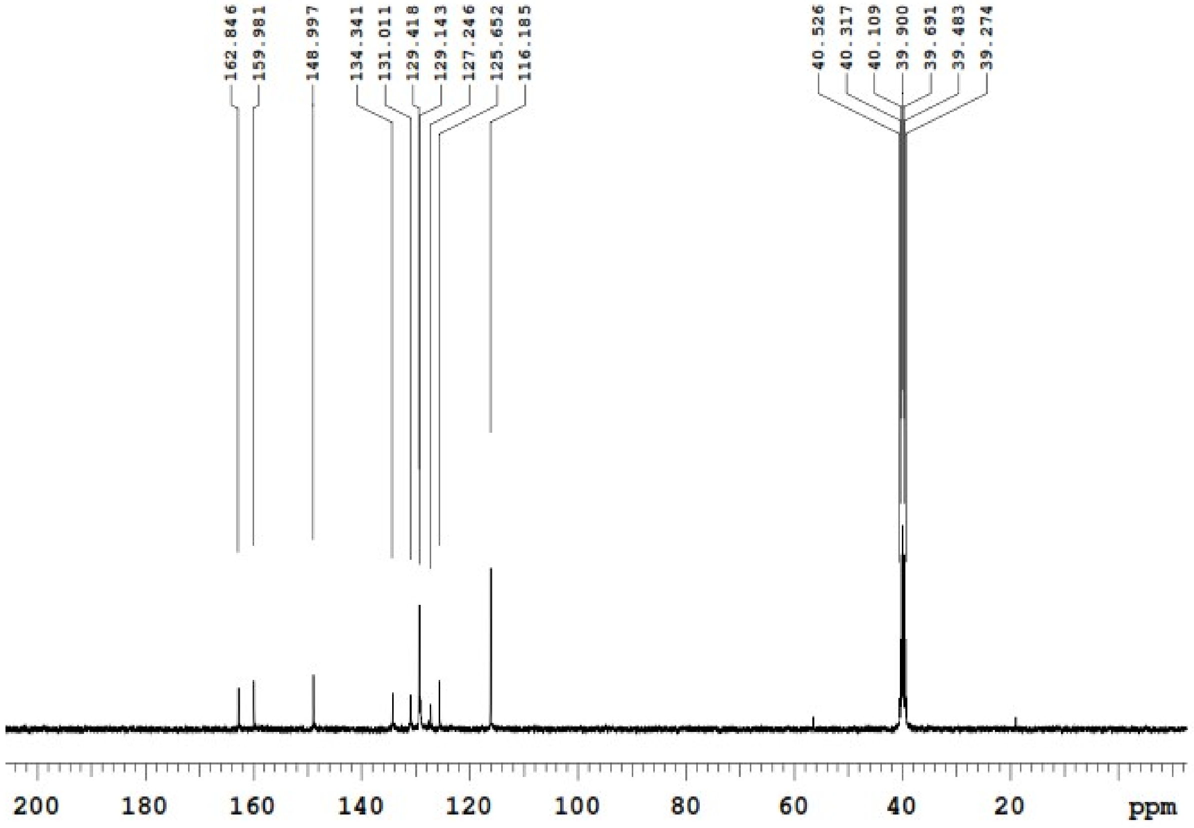

^{13}C NMR spectra of compound no 9

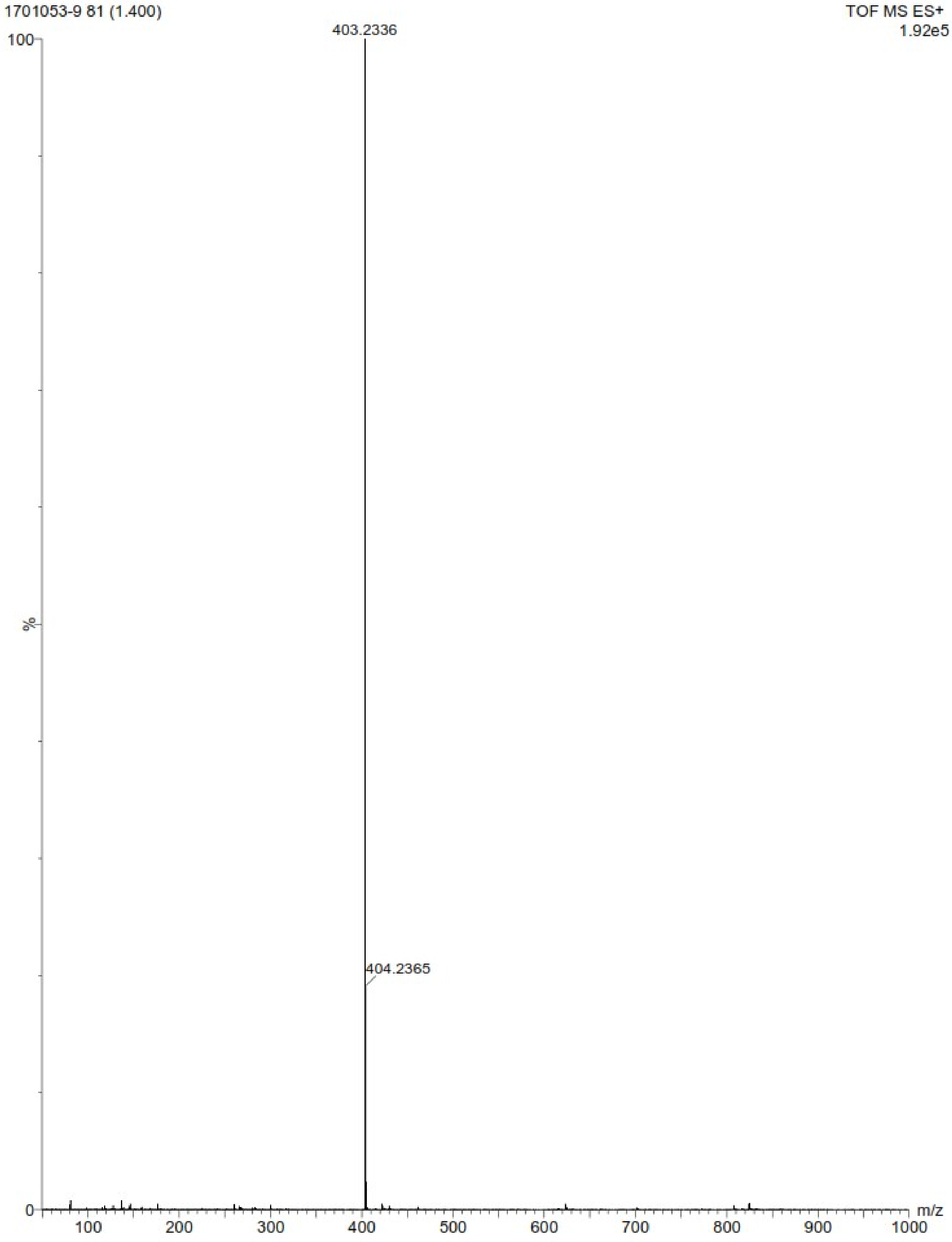

Mass spectra of compound no 9

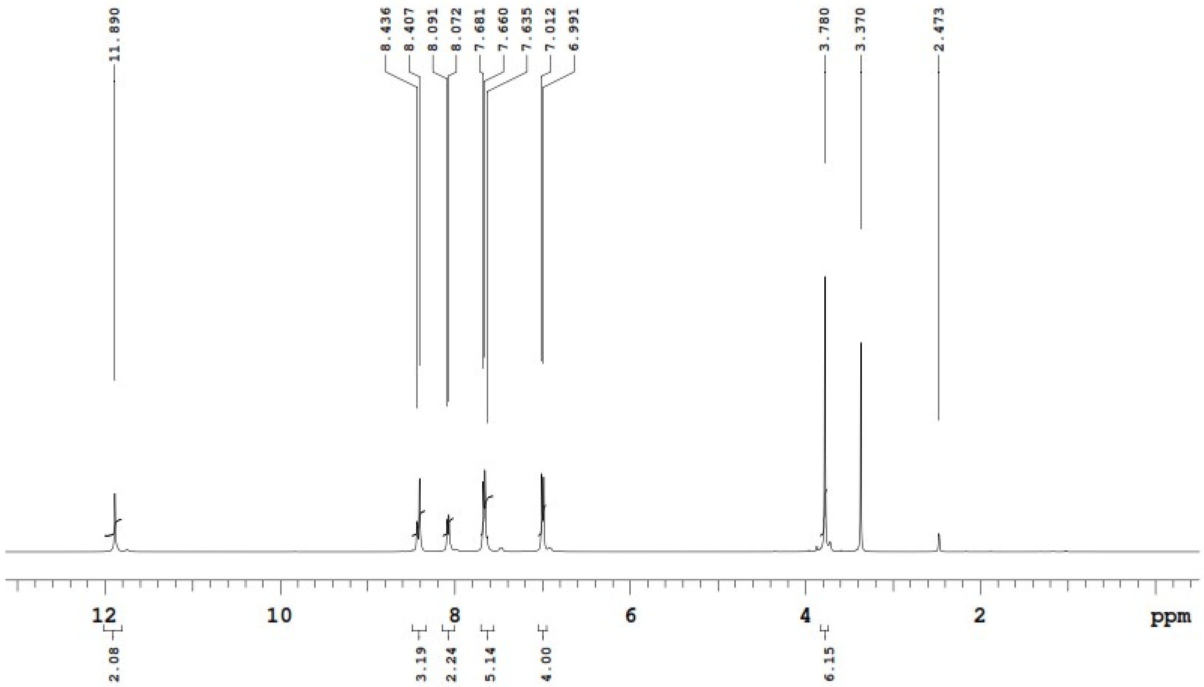

^{1}H NMR spectra of compound no 10

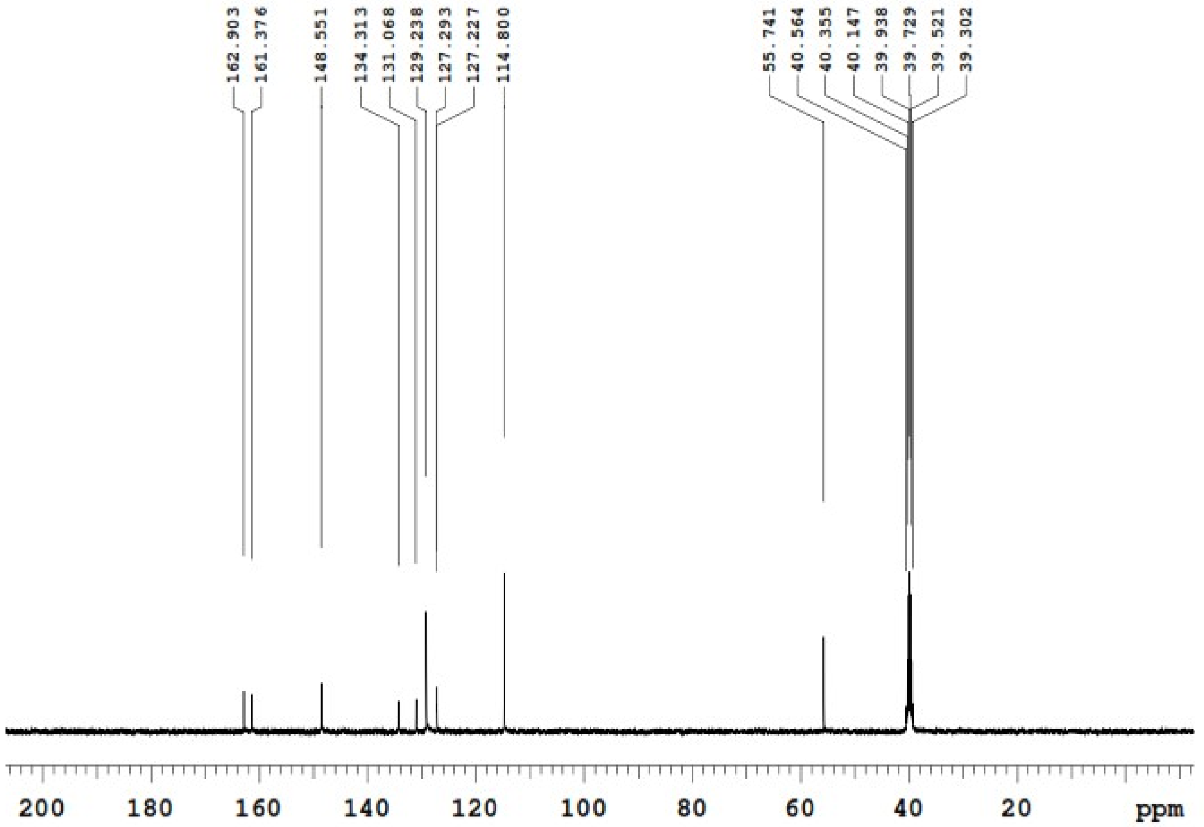

^{13}C NMR spectra of compound no 10

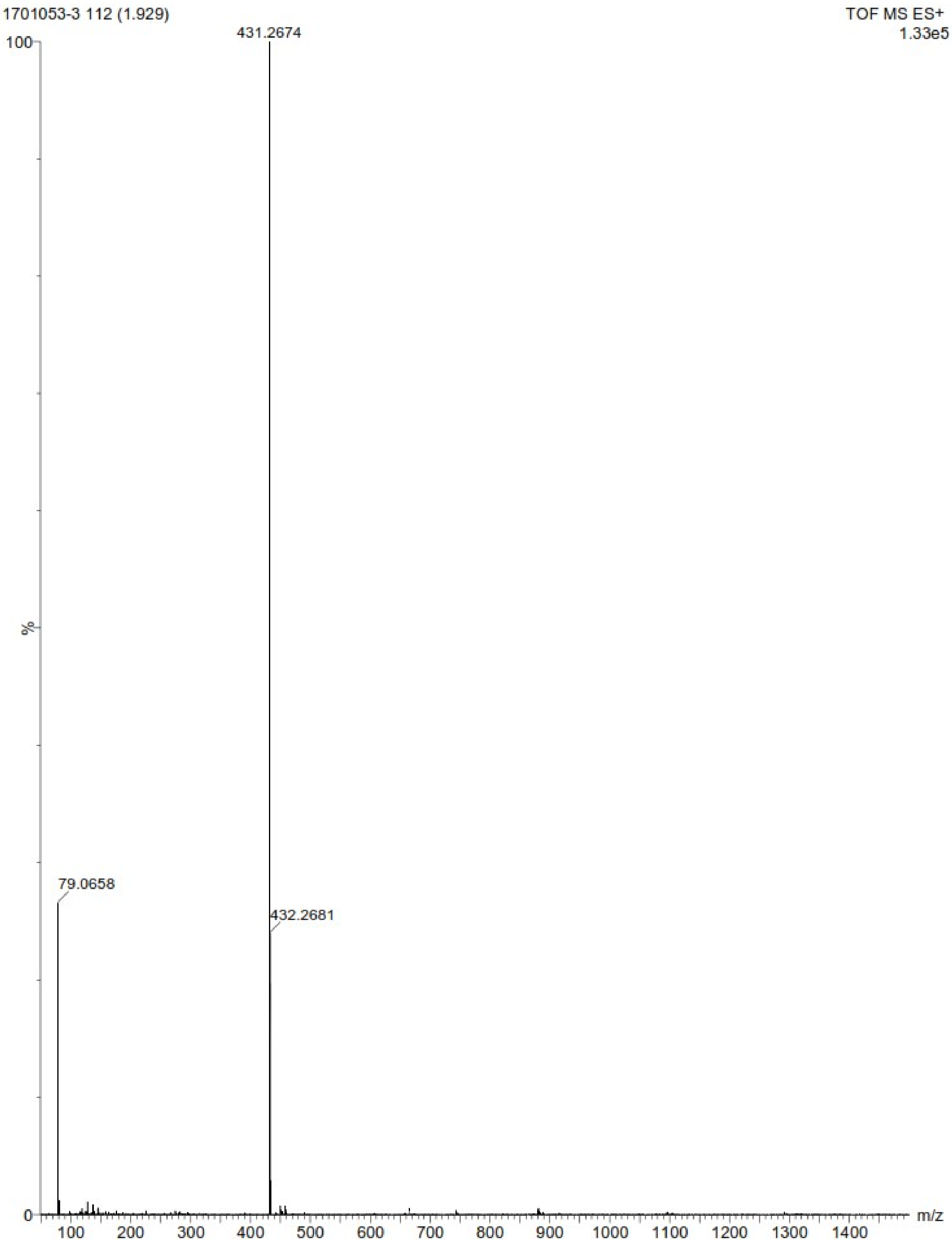

Mass spectra of compound no 10

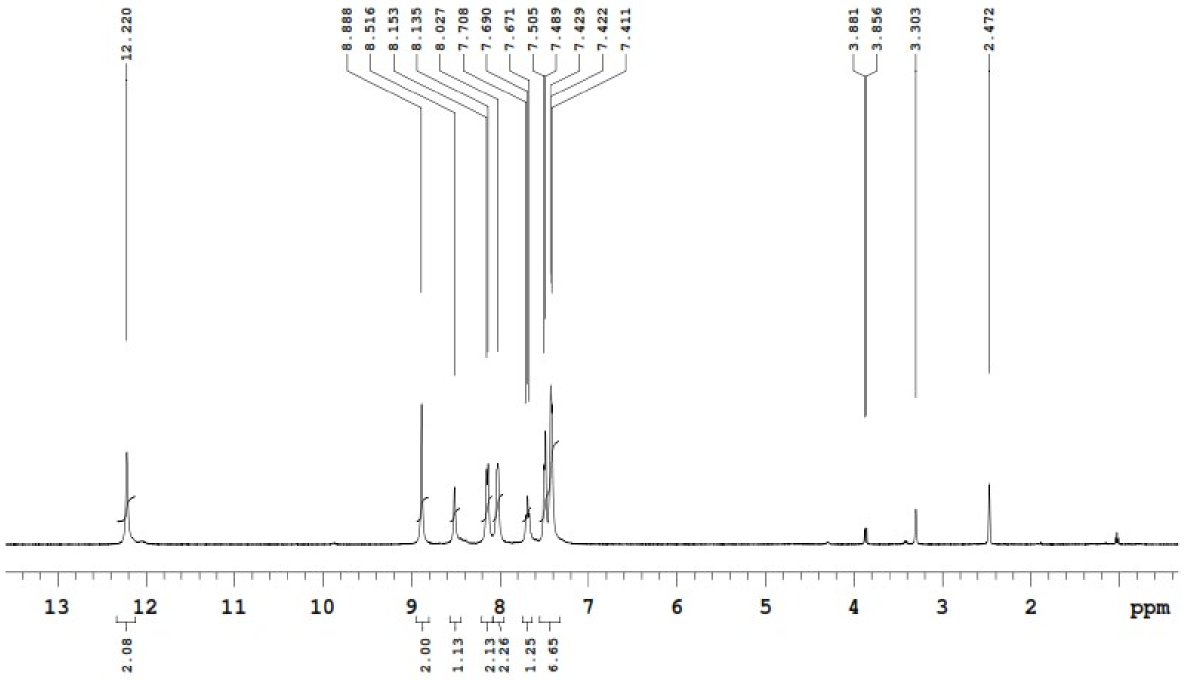

^{1}H NMR spectra of compound no 11

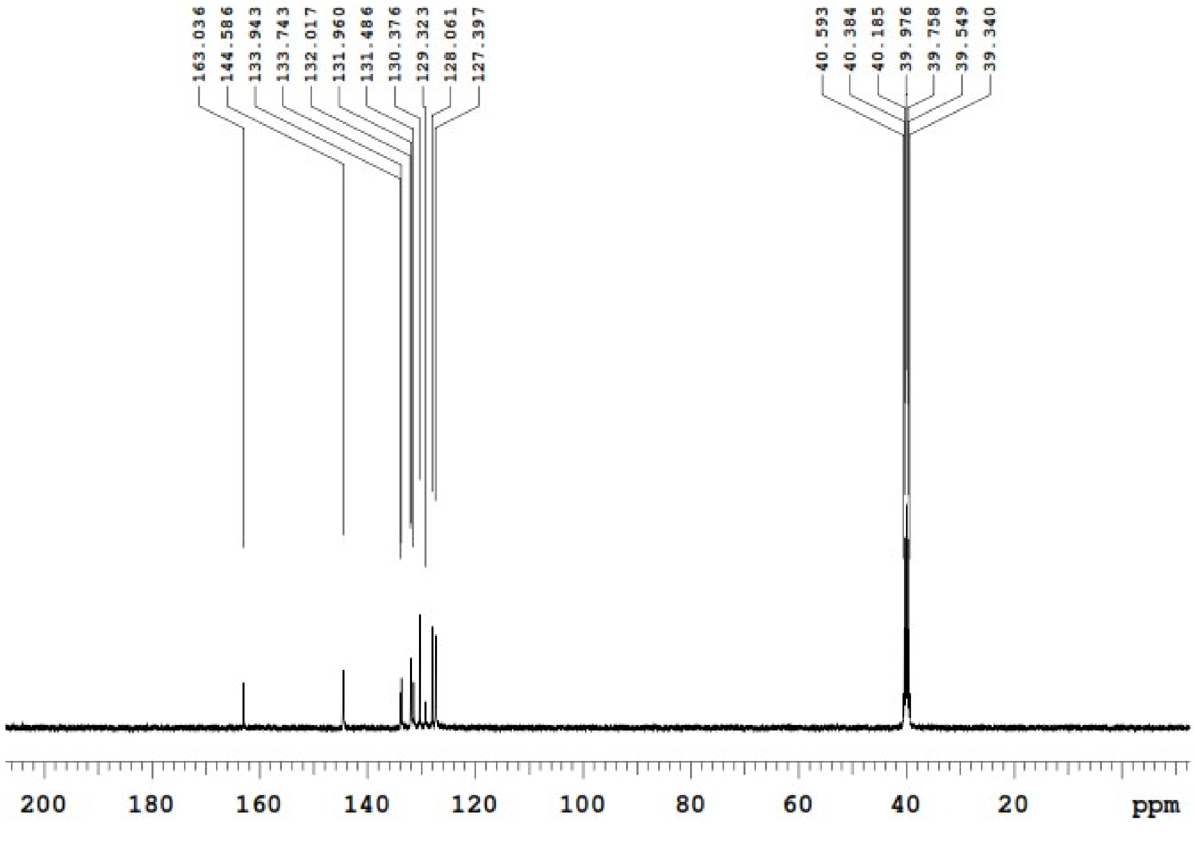

^{13}C NMR spectra of compound no 11

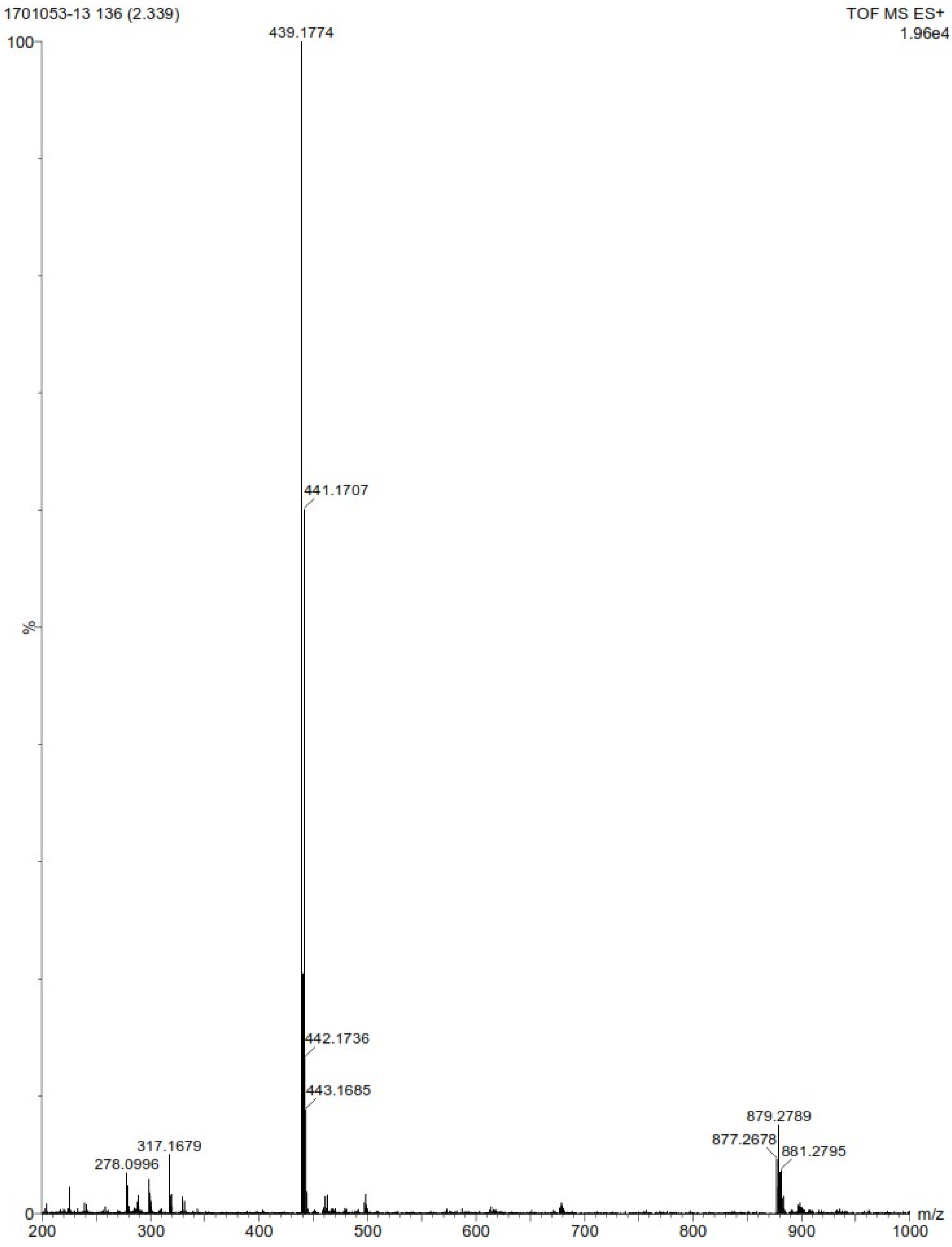

Mass spectra of compound no 11

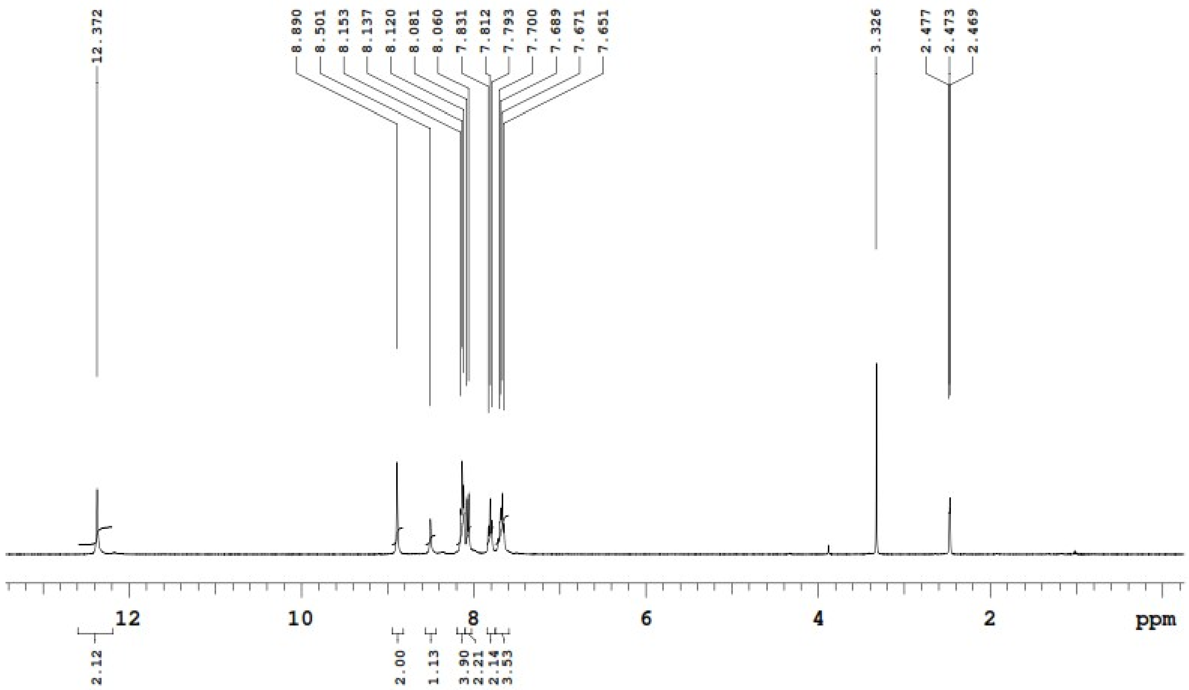

^{1}H NMR spectra of compound no 12

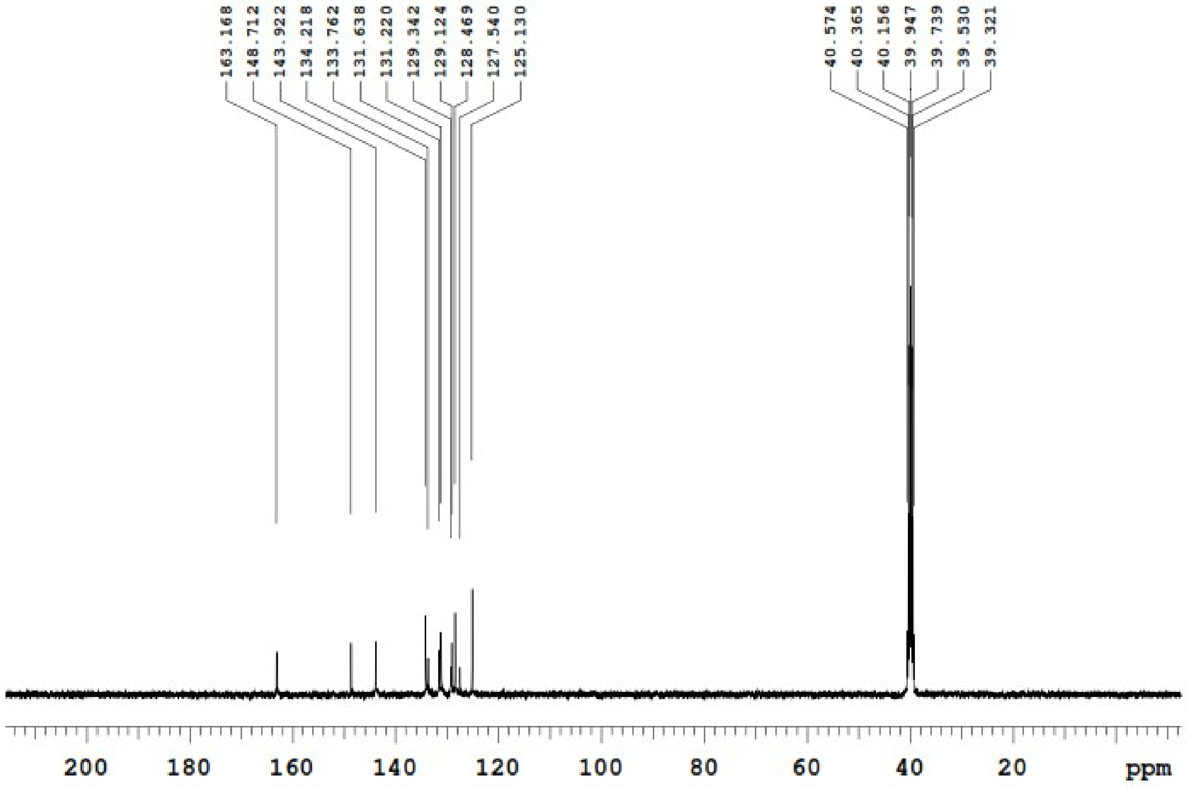

^{13}C NMR spectra of compound no 12

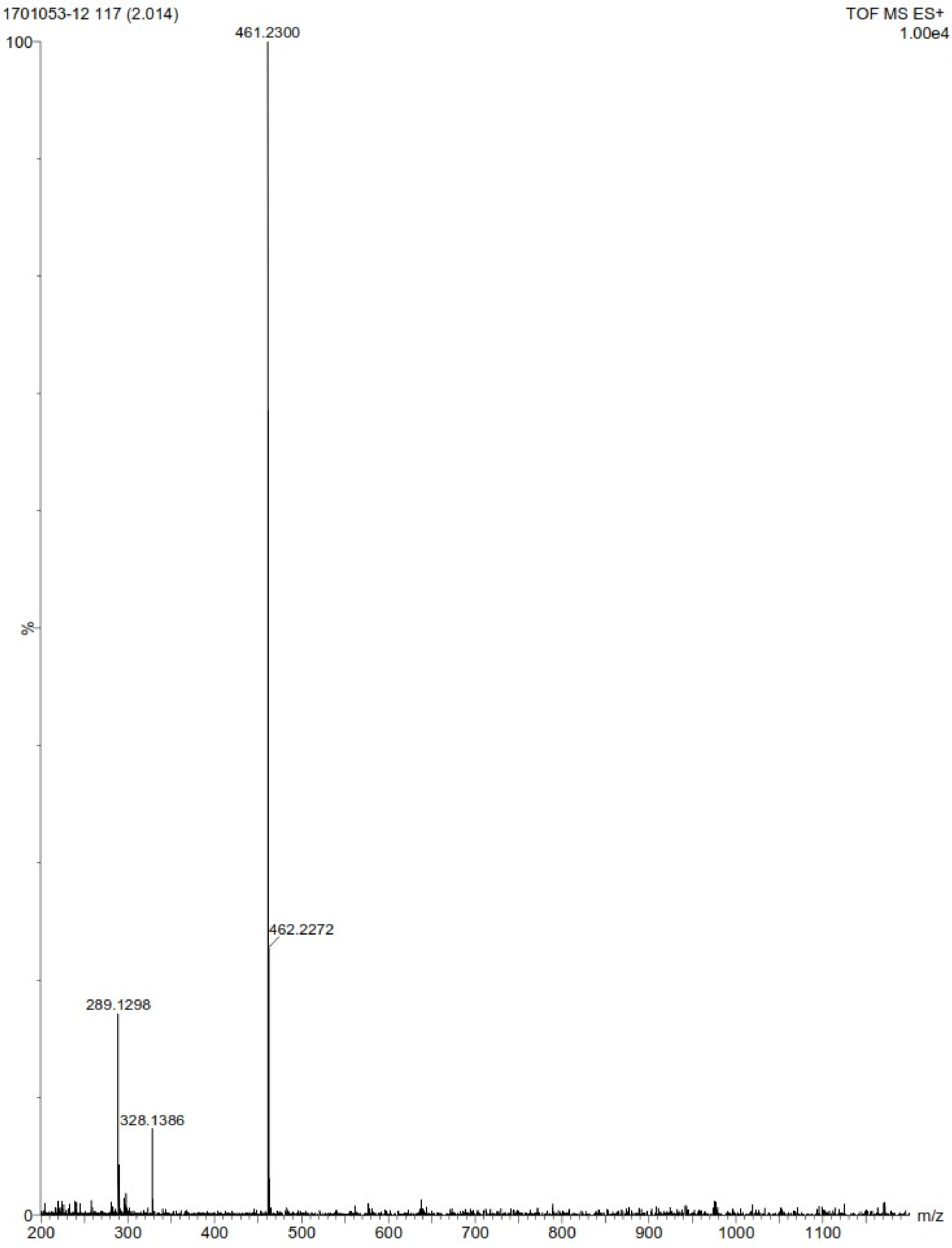

Mass spectra of compound no 12

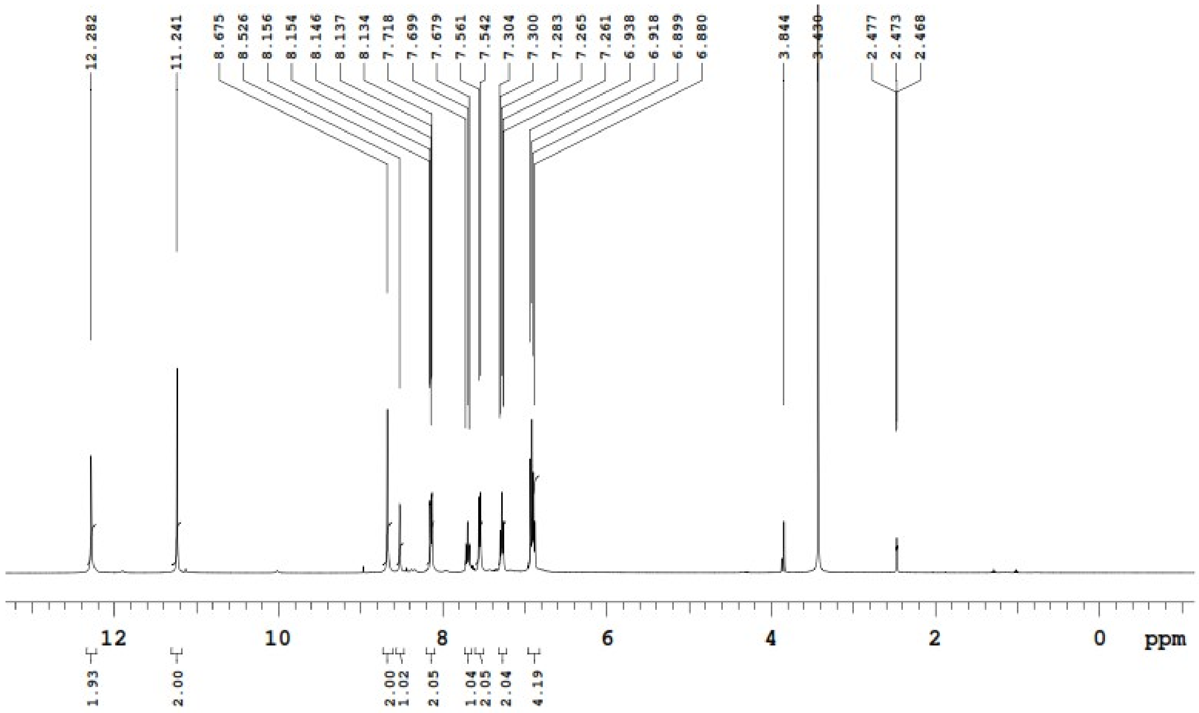

^{1}H NMR spectra of compound no 15

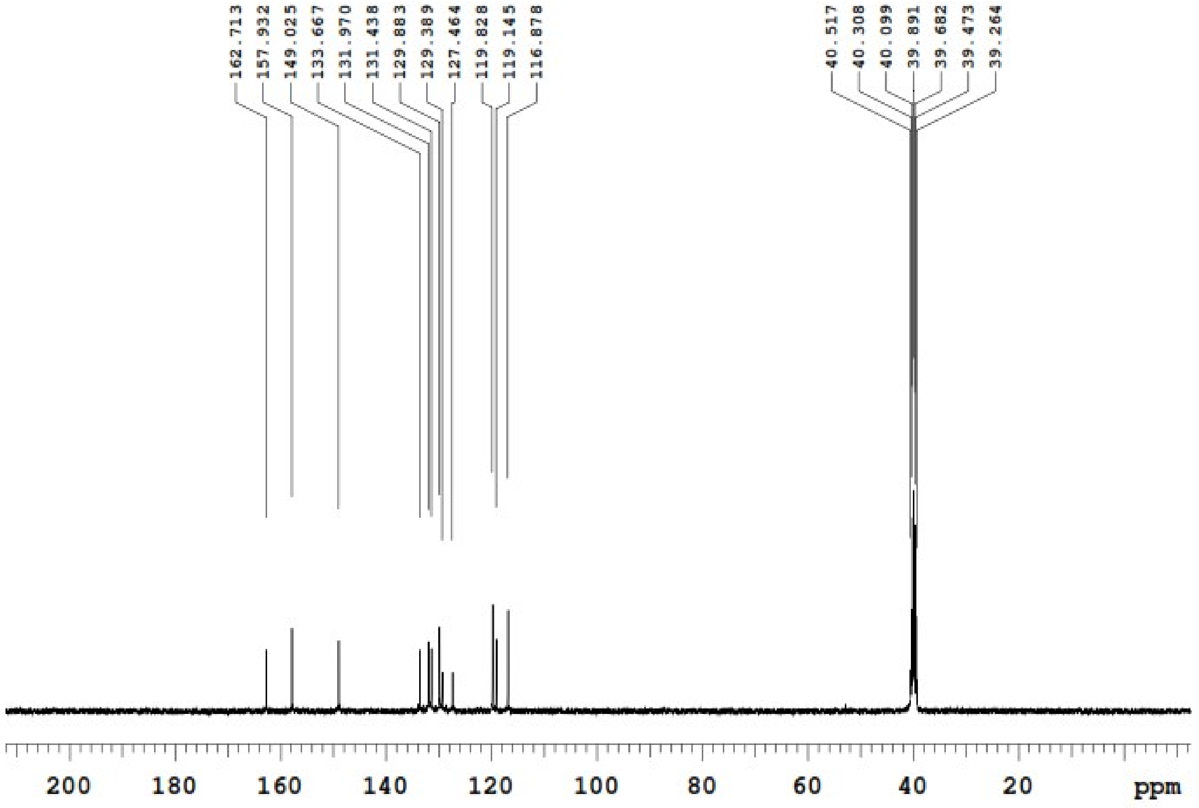

^{13}C NMR spectra of compound no 15

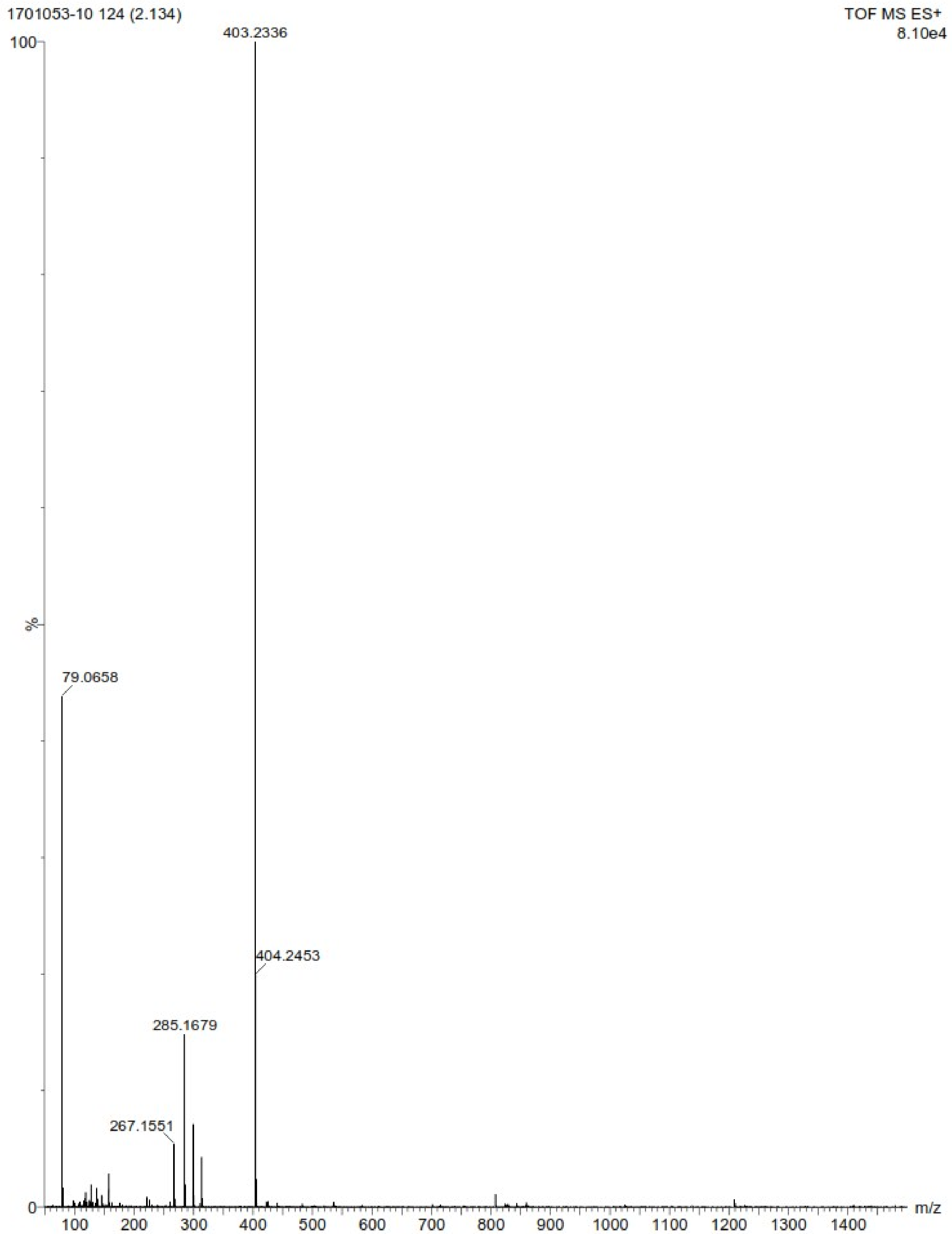

Mass spectra of compound no 15

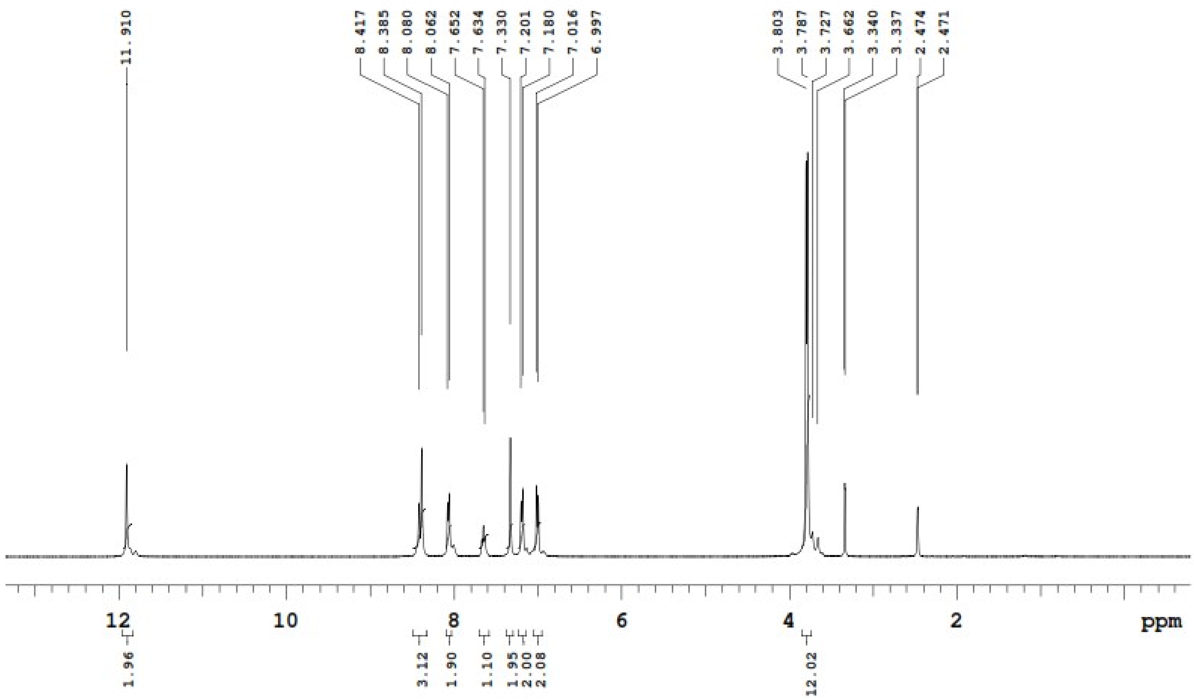

^{1}H NMR spectra of compound no 18

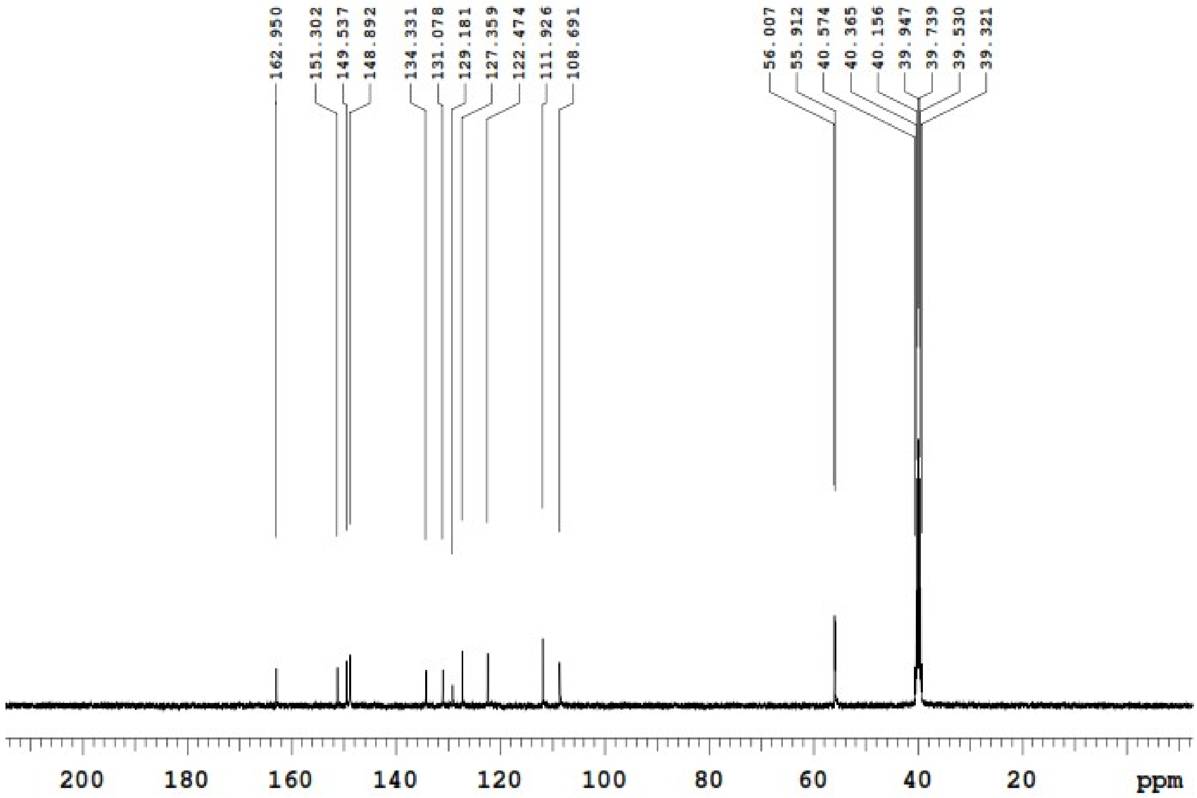

^{13}C NMR spectra of compound no 18

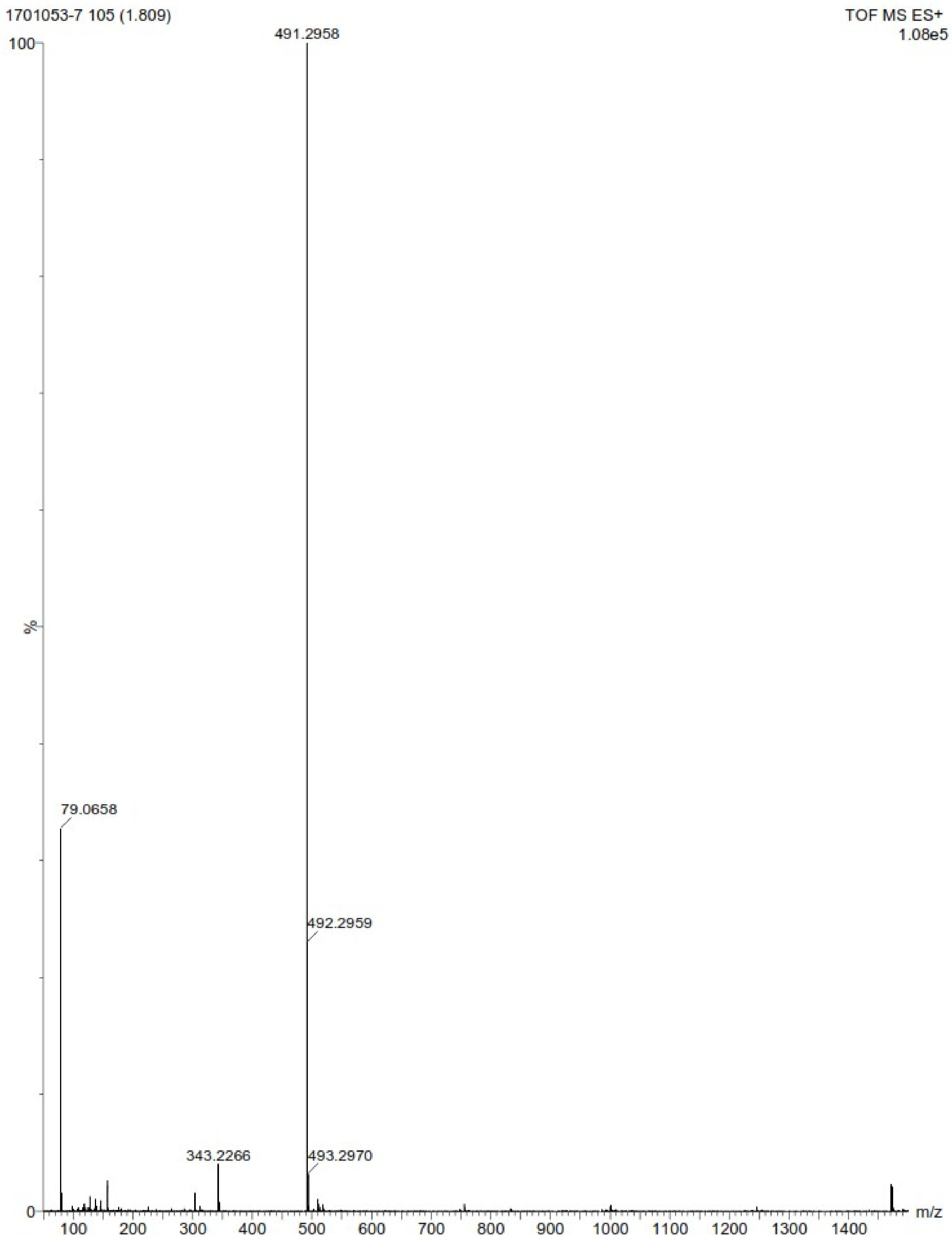

Mass spectra of compound no 18

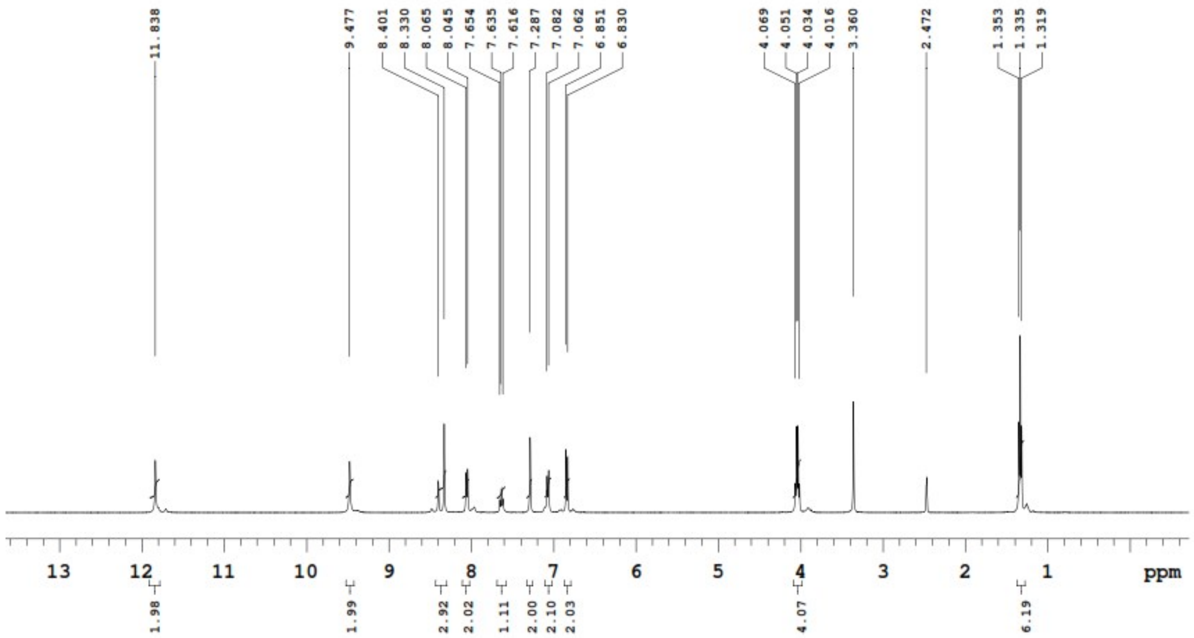

^{1}H NMR spectra of compound no 19

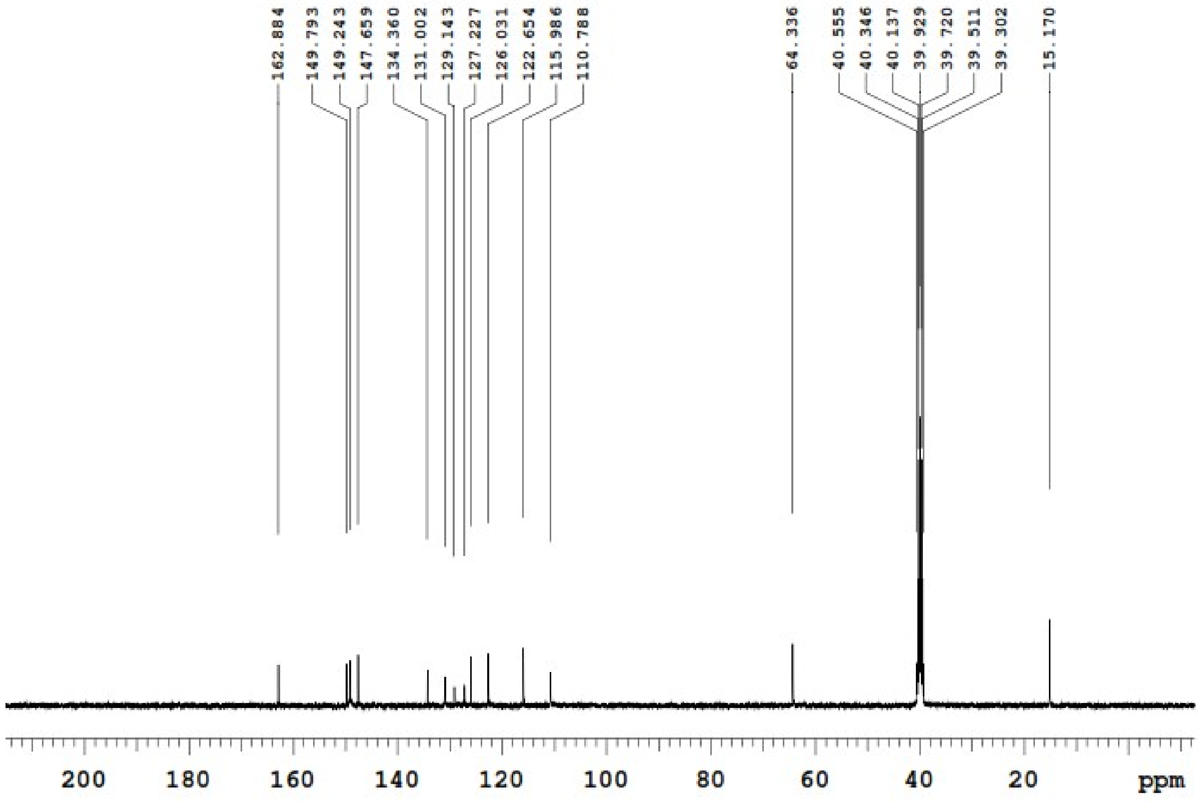

^{13}C NMR spectra of compound no 19

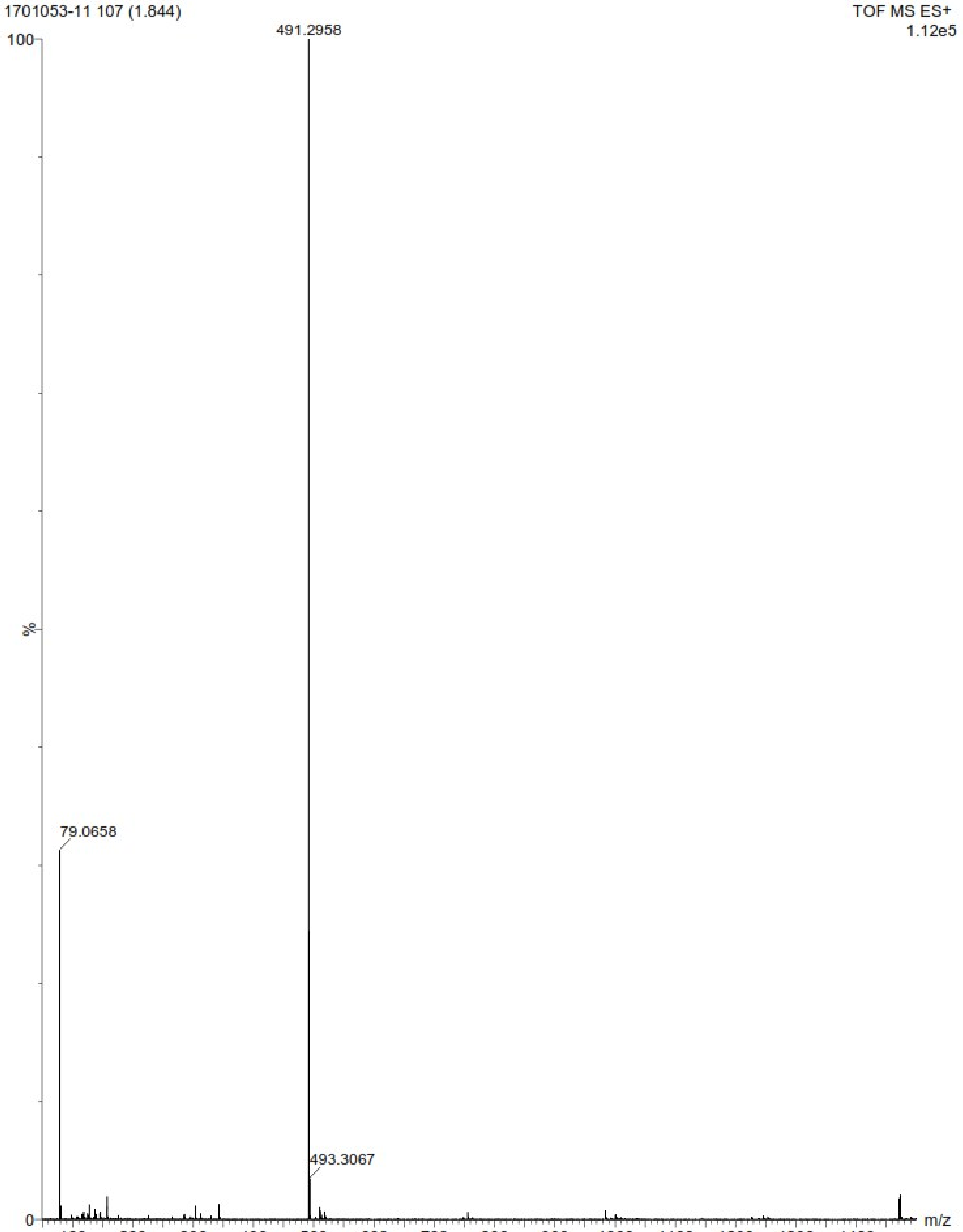

Mass spectra of compound no 19

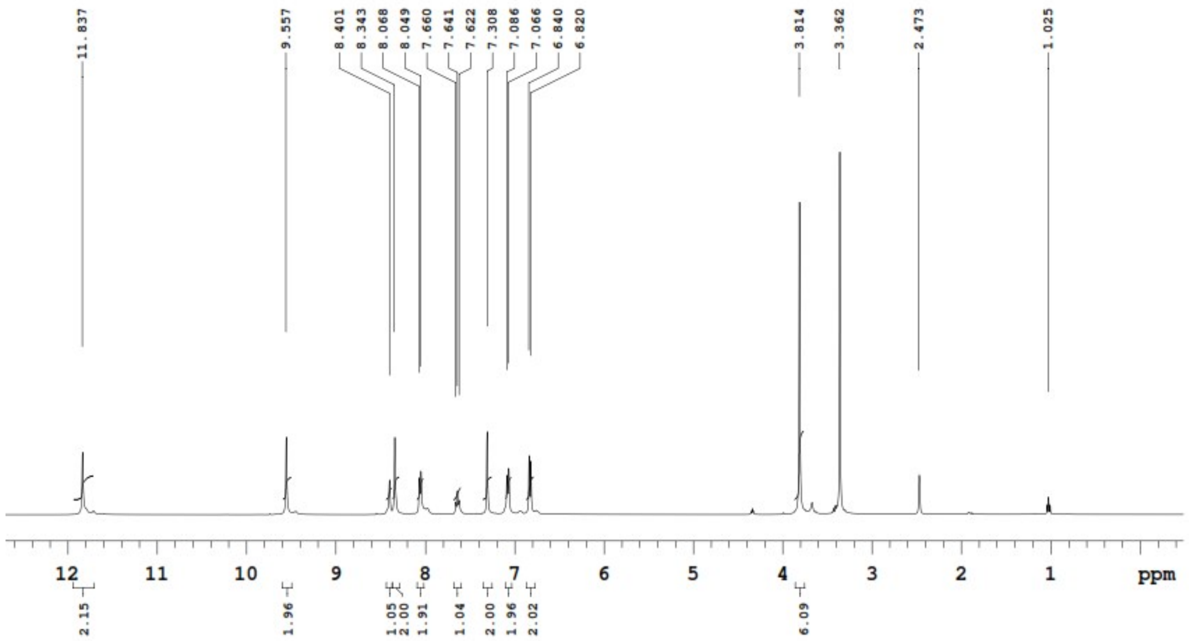

^{1}H NMR spectra of compound no 20

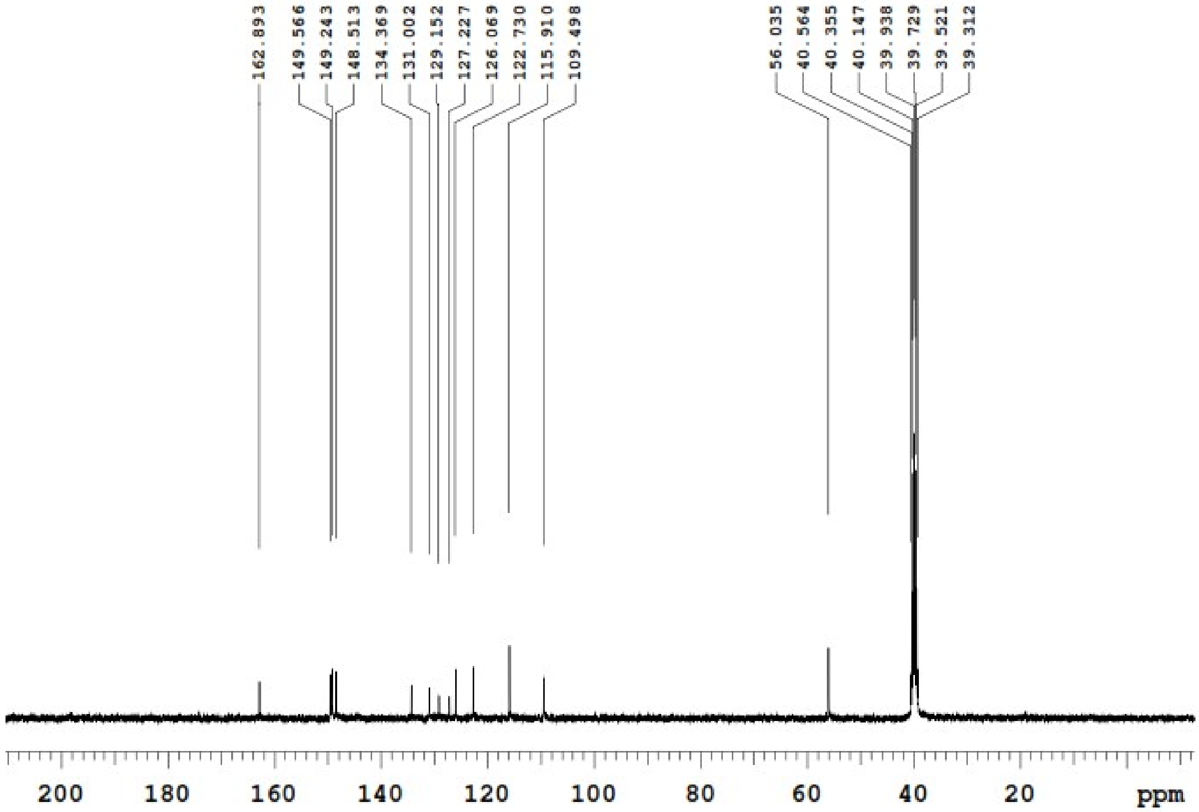

^{13}C NMR spectra of compound no 20

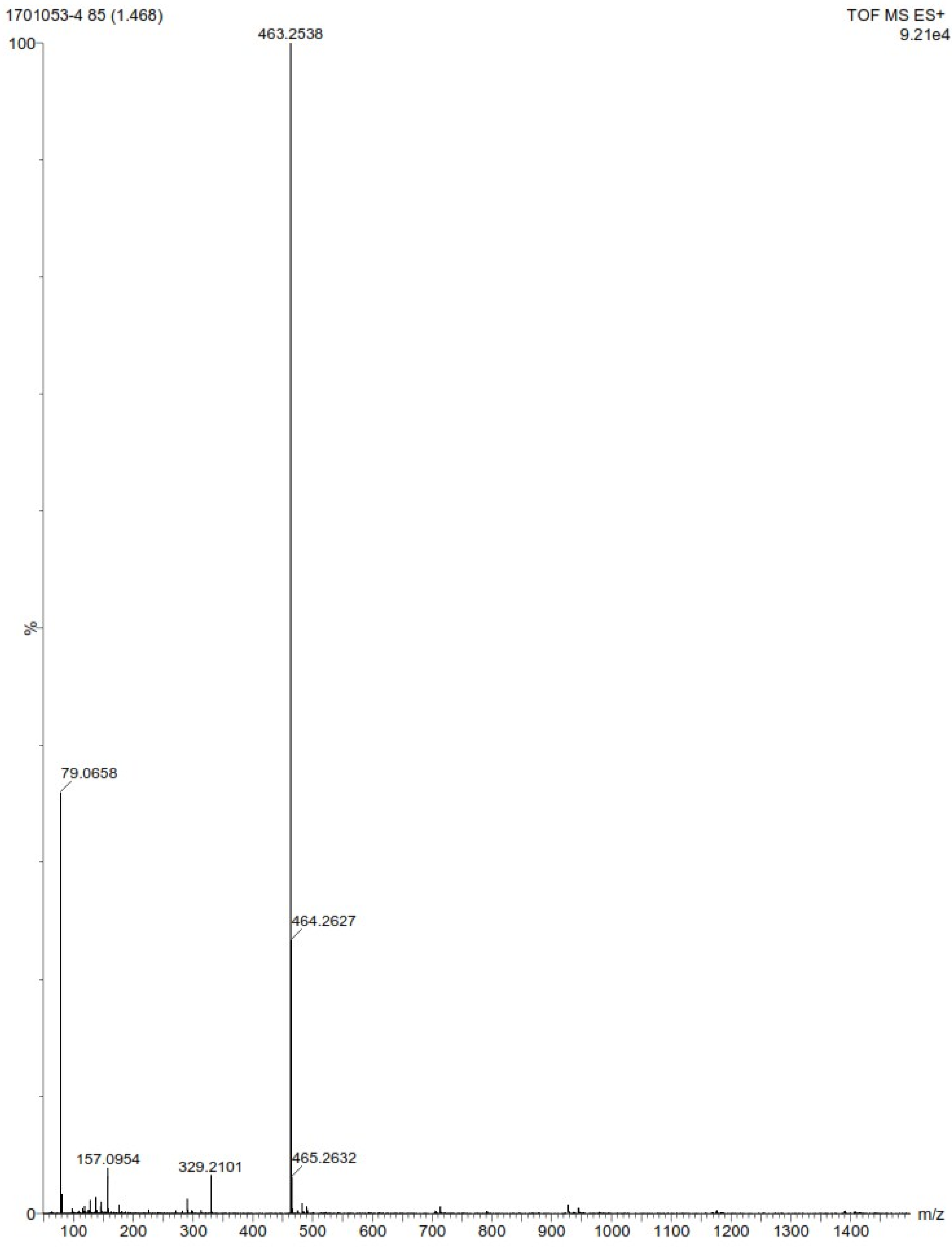

Mass spectra of compound no 20

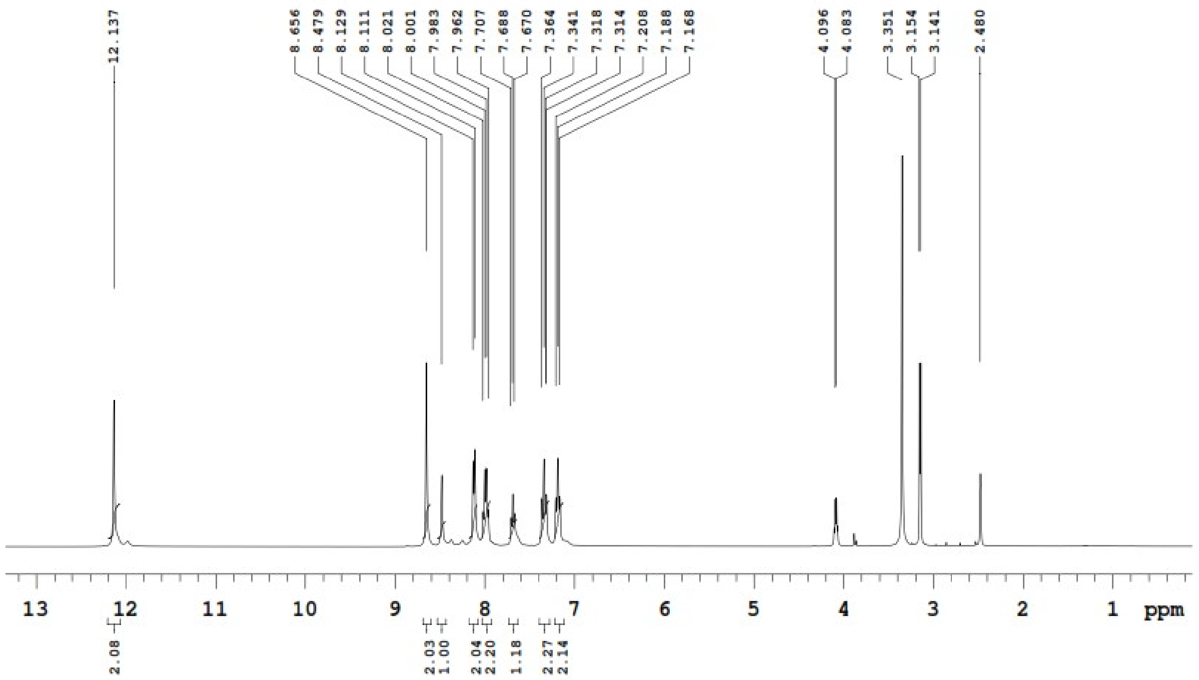

^{1}H NMR spectra of compound no 23

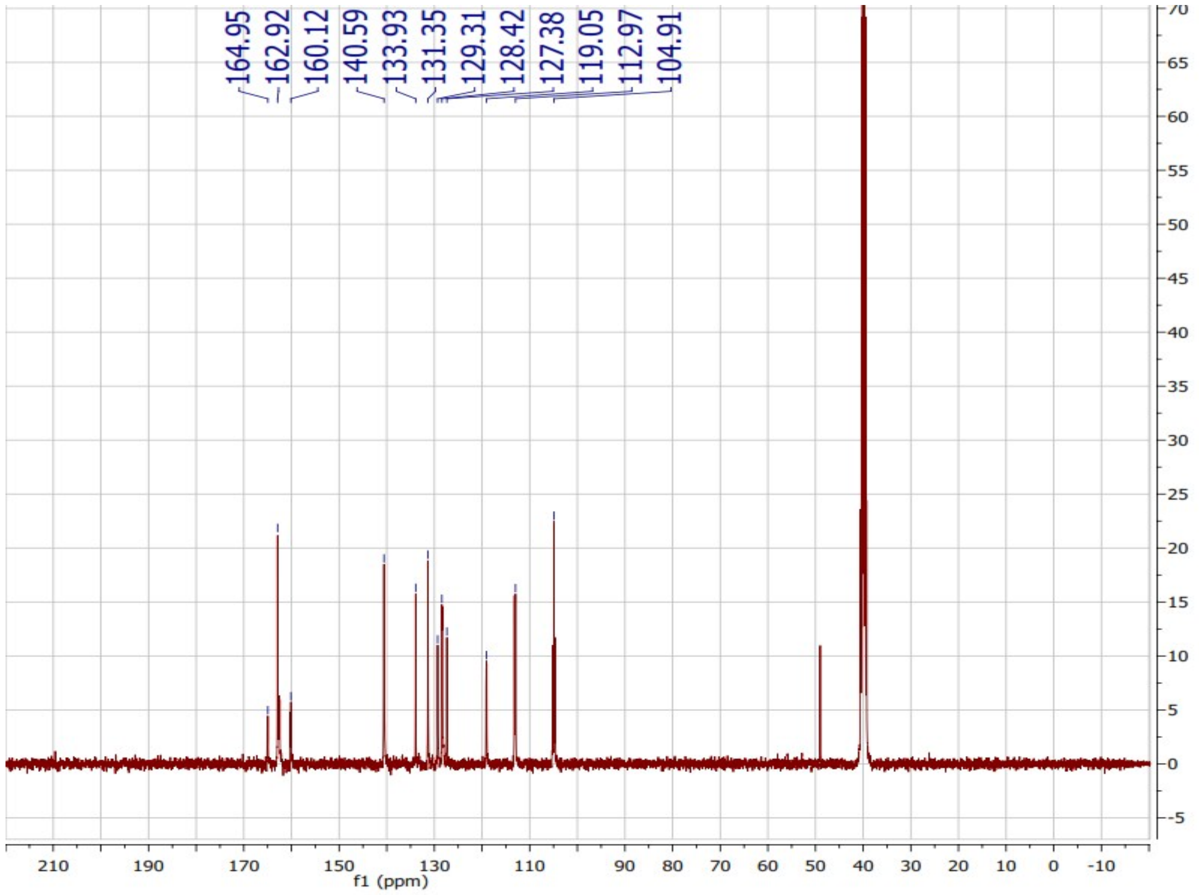

^{13}C NMR spectra of compound no 23

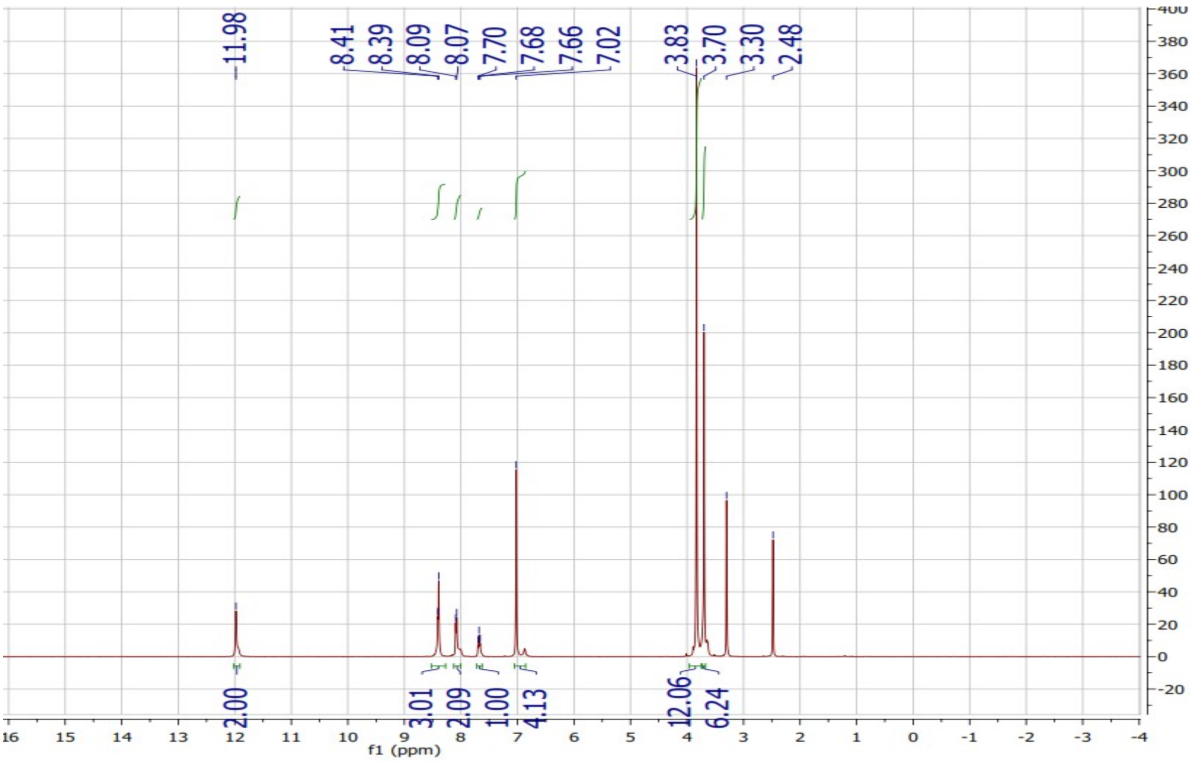

^{1}H NMR spectra of compound no 26

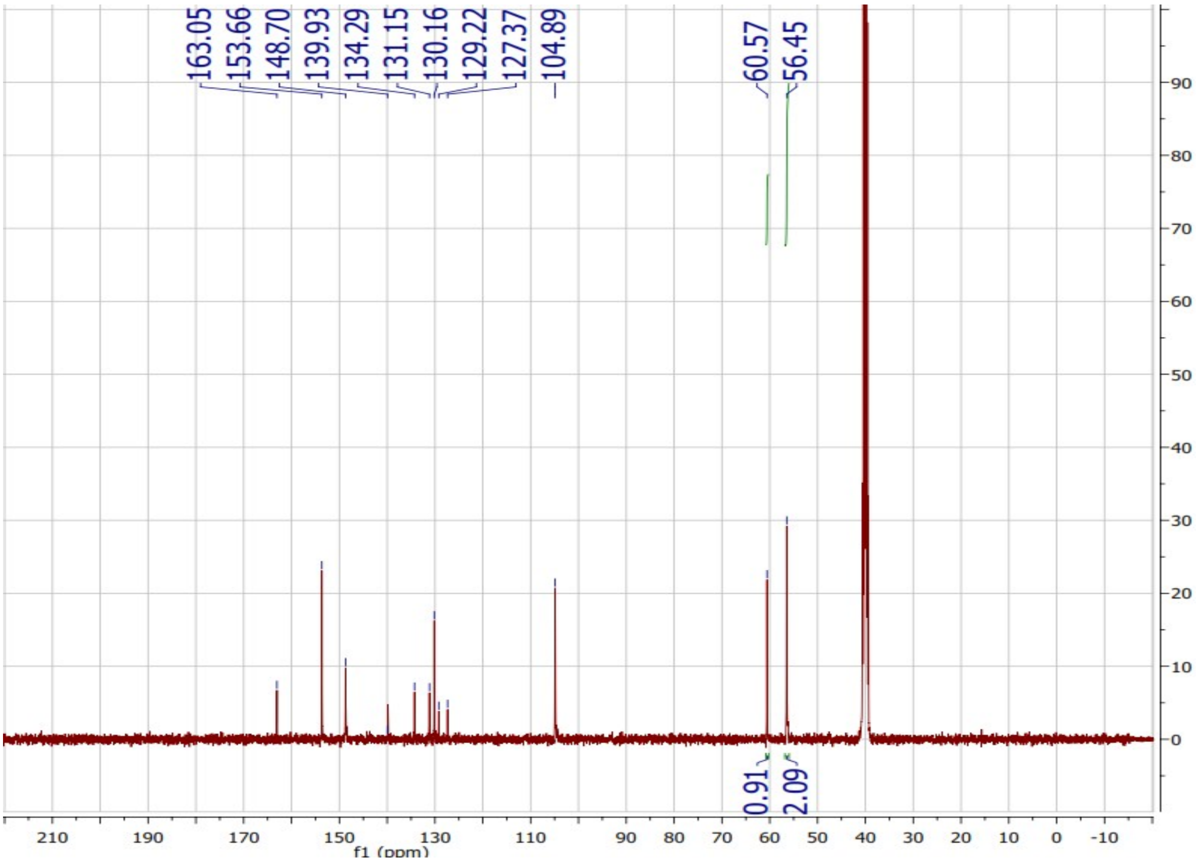

^{13}C NMR spectra of compound no 26

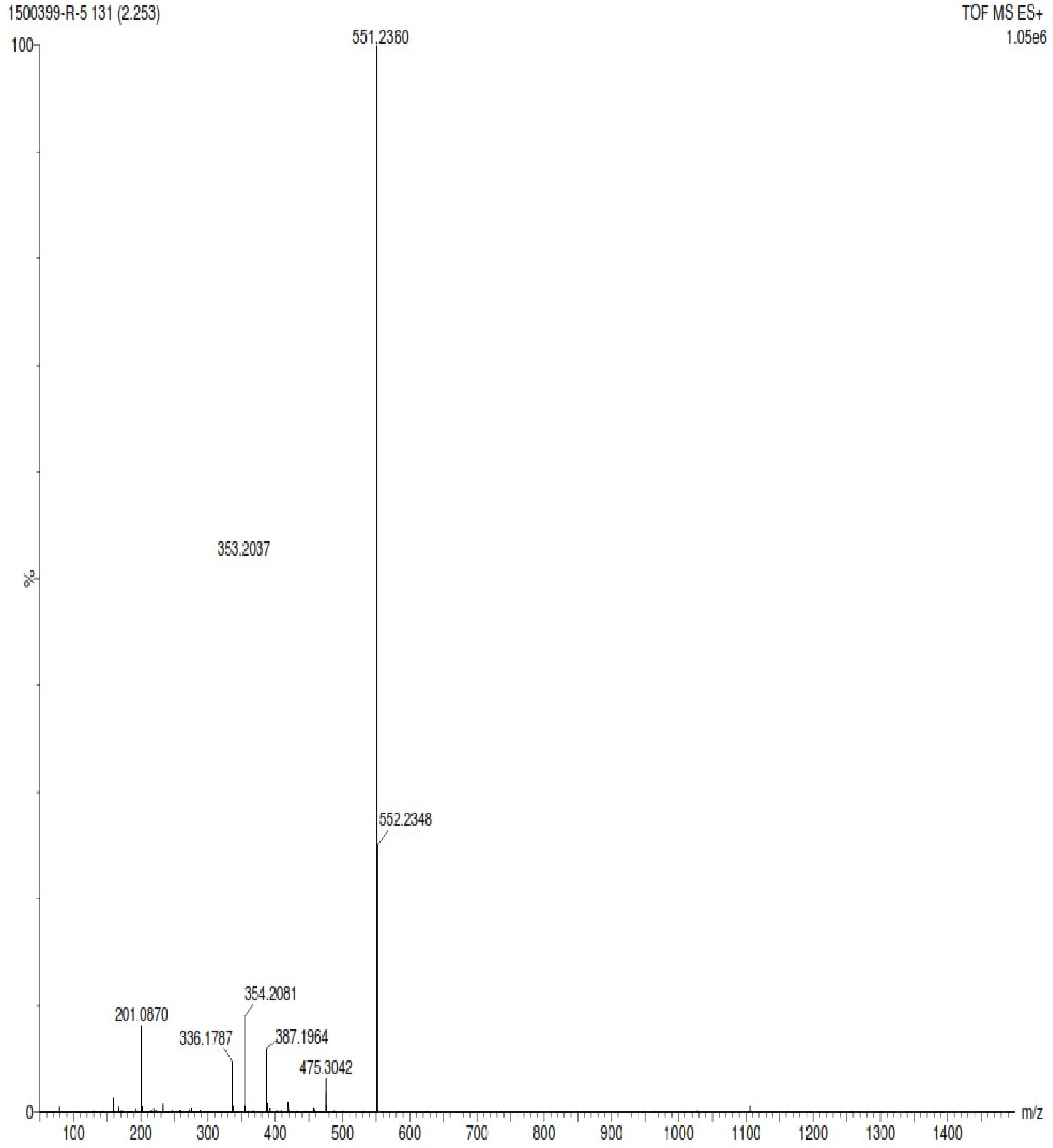

Mass spectra of compound no 26

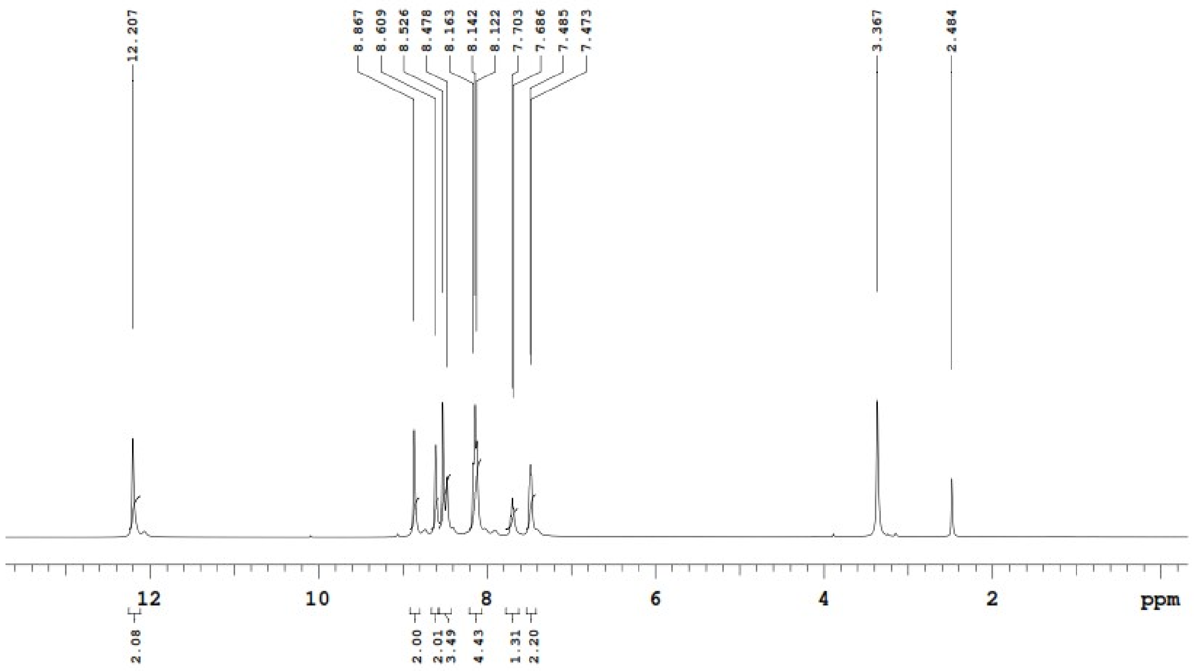

^{1}H NMR spectra of compound no 30

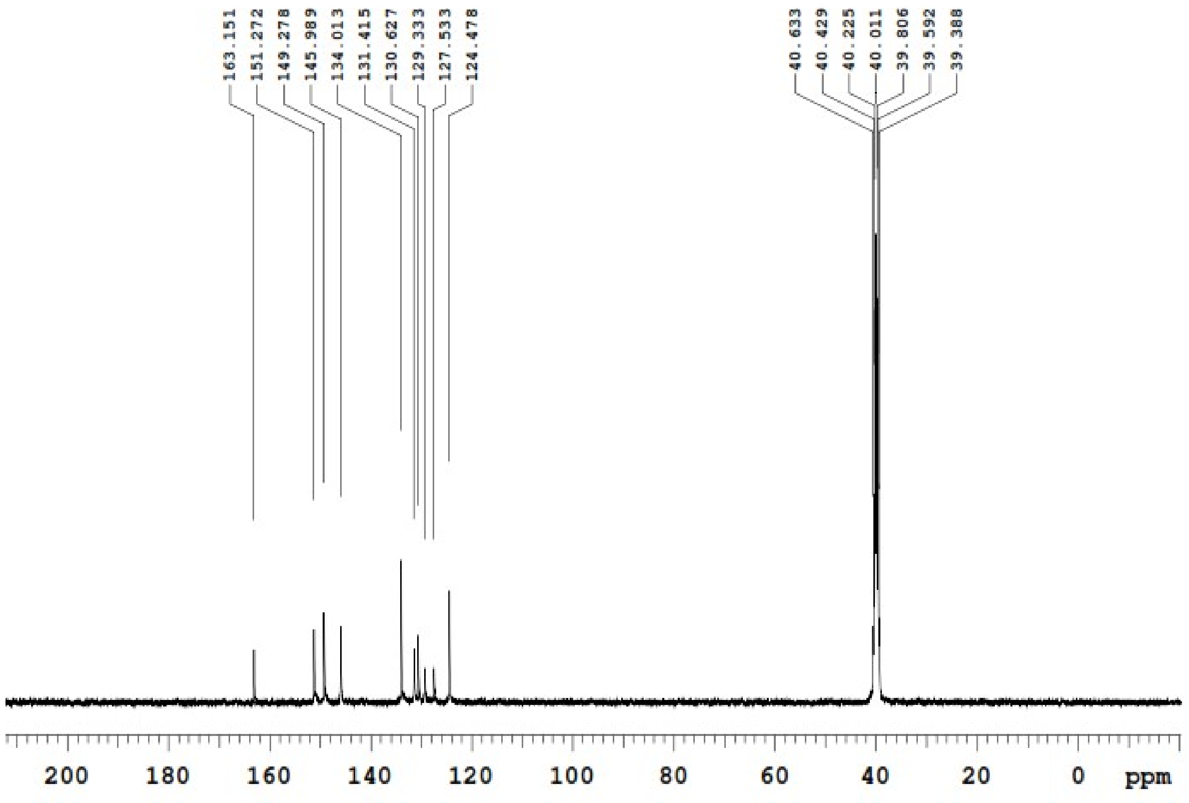

^{13}C NMR spectra of compound no 30

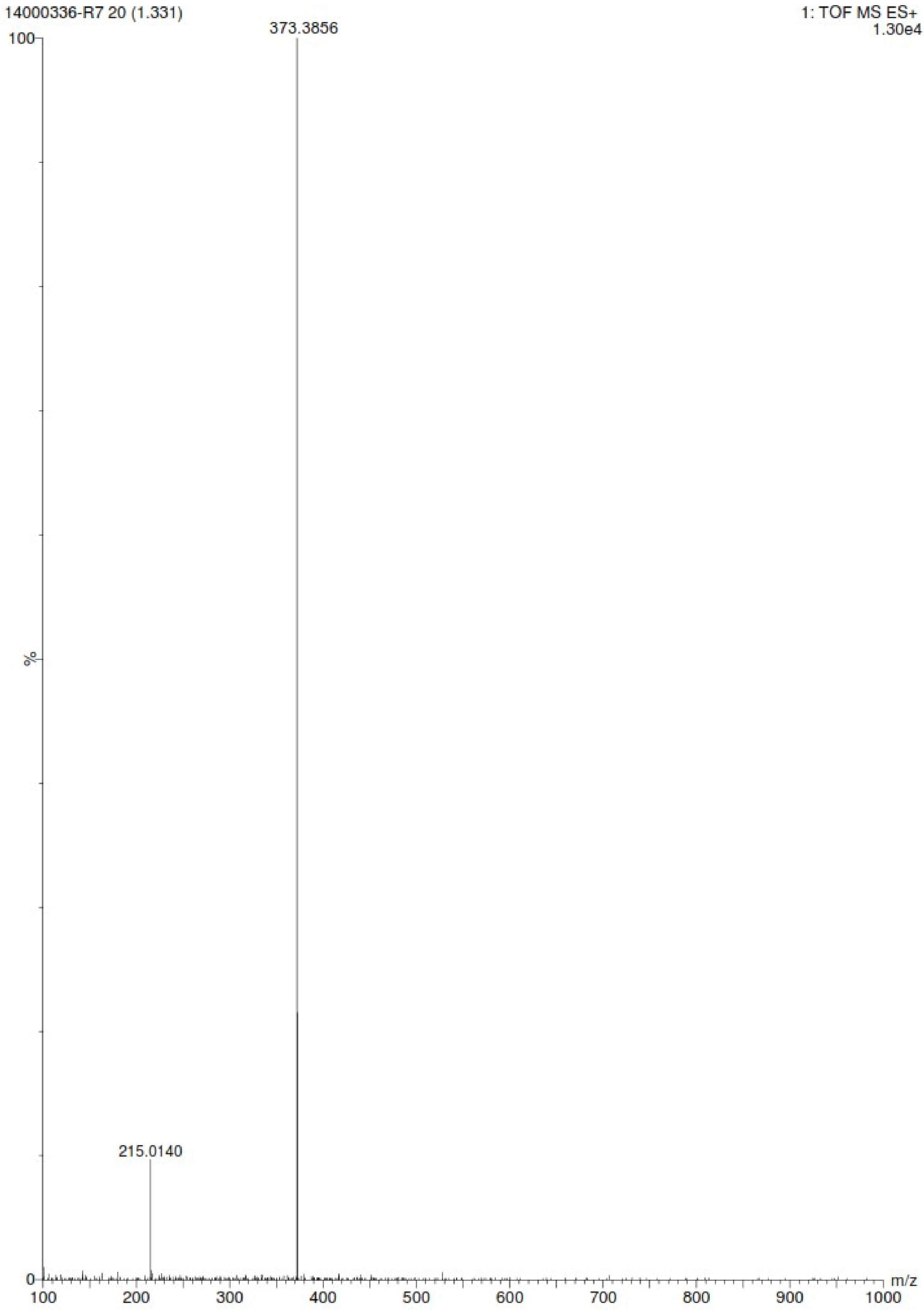

Mass spectra of compound no 30

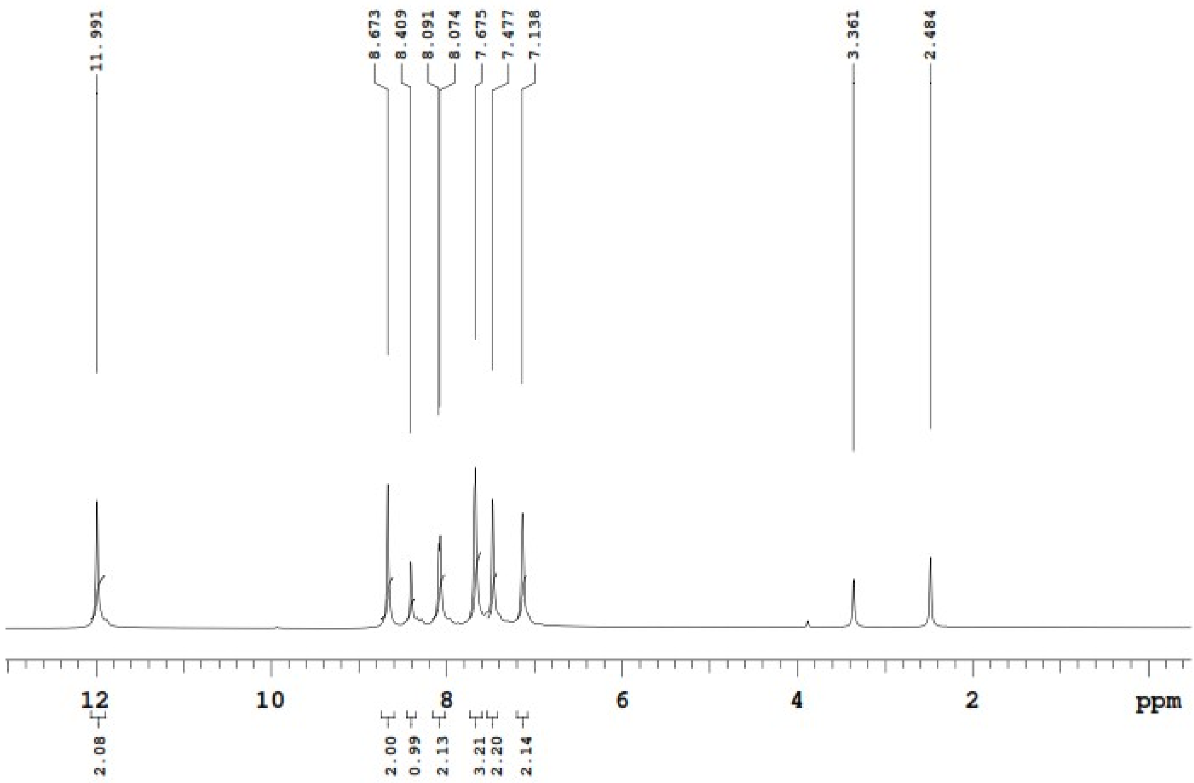

^{1}H NMR spectra of compound no 32

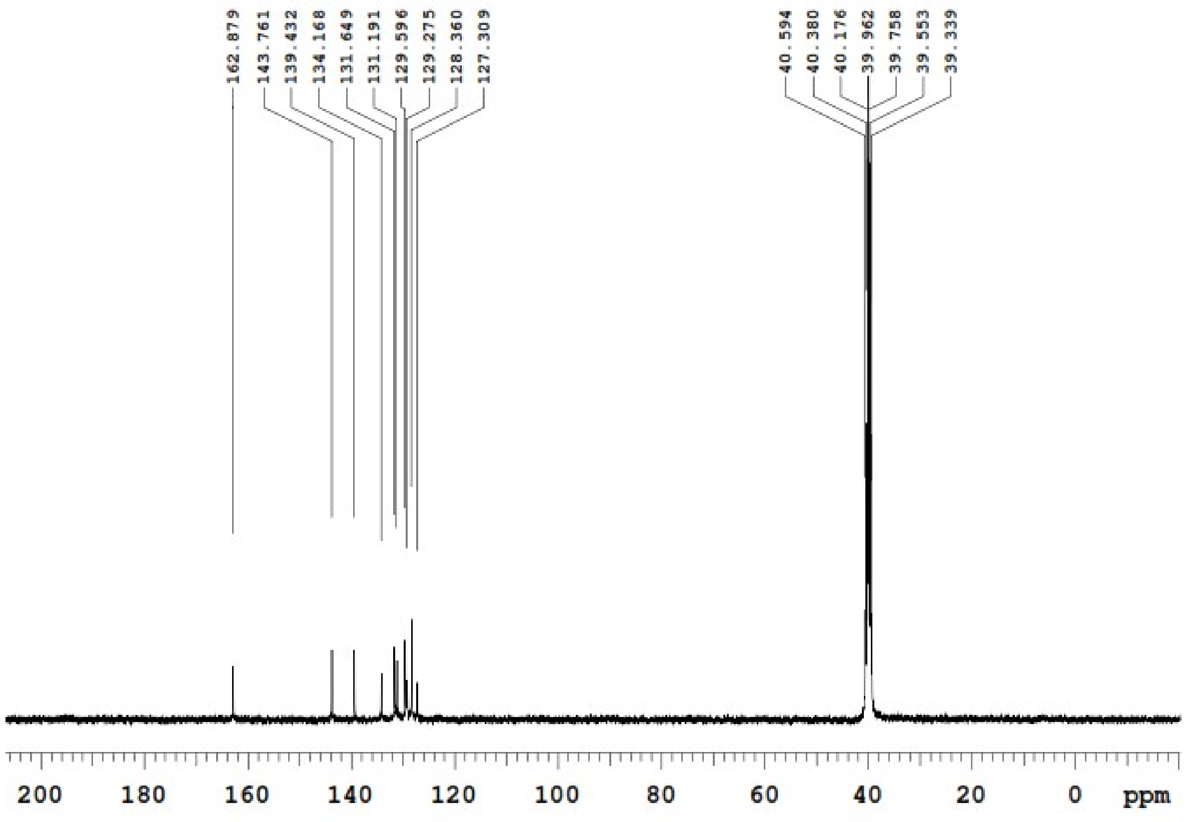

^{13}C NMR spectra of compound no 32

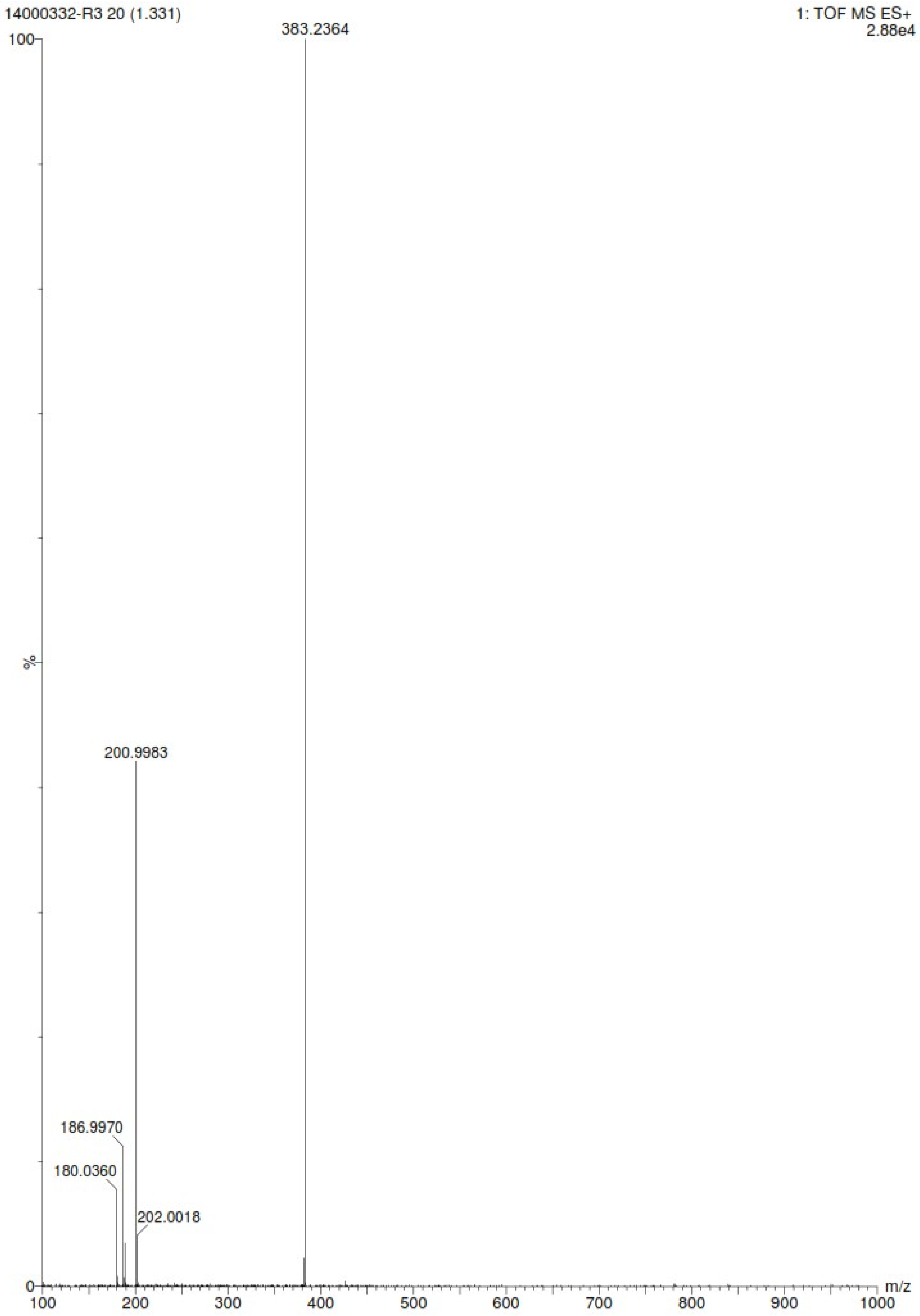

Mass spectra of compound no 32

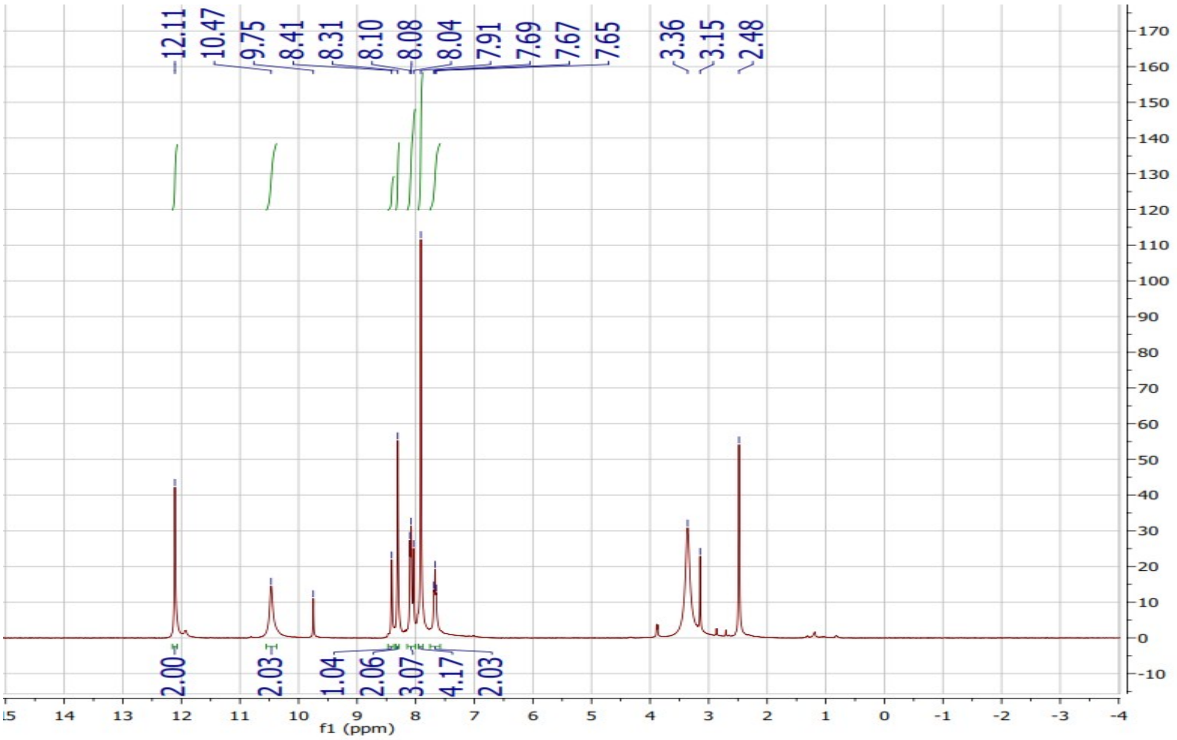

^{1}H NMR spectra of compound no 34

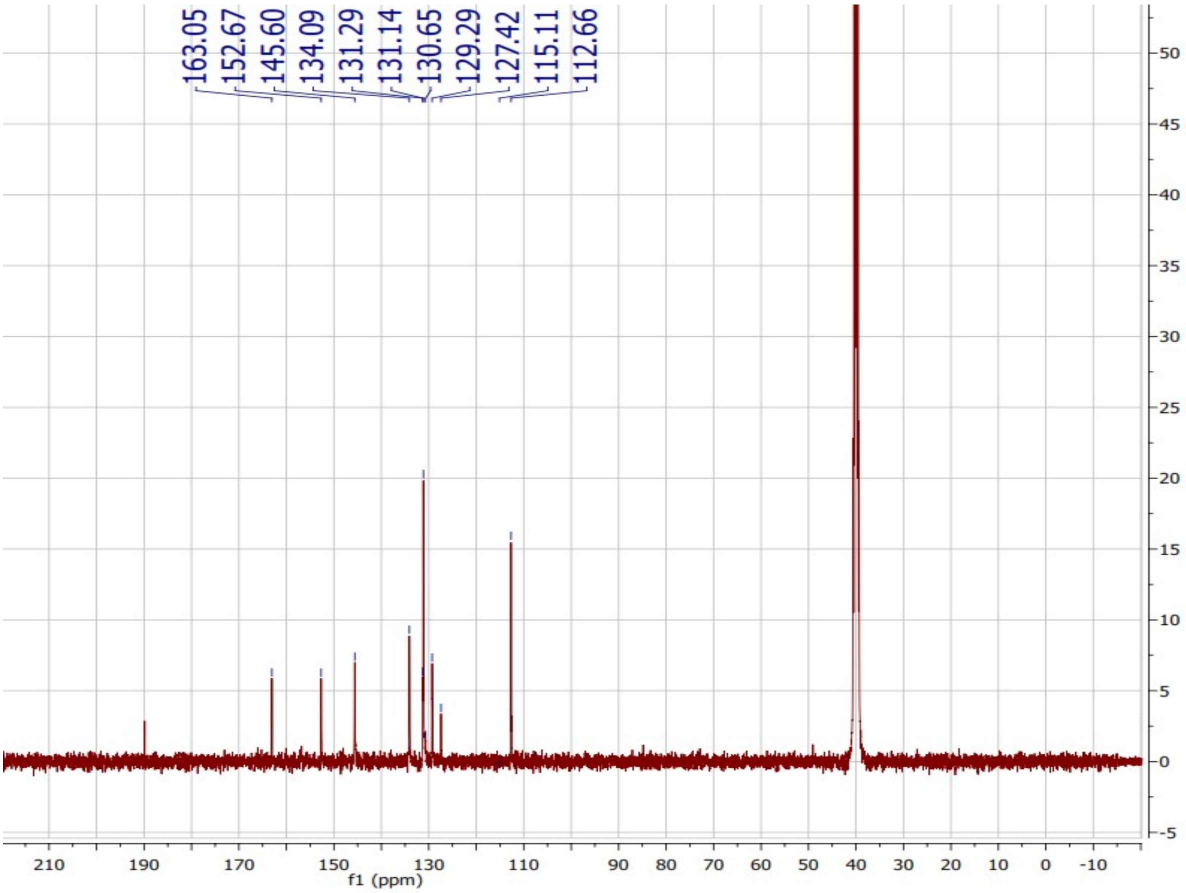

^{13}C NMR spectra of compound no 34

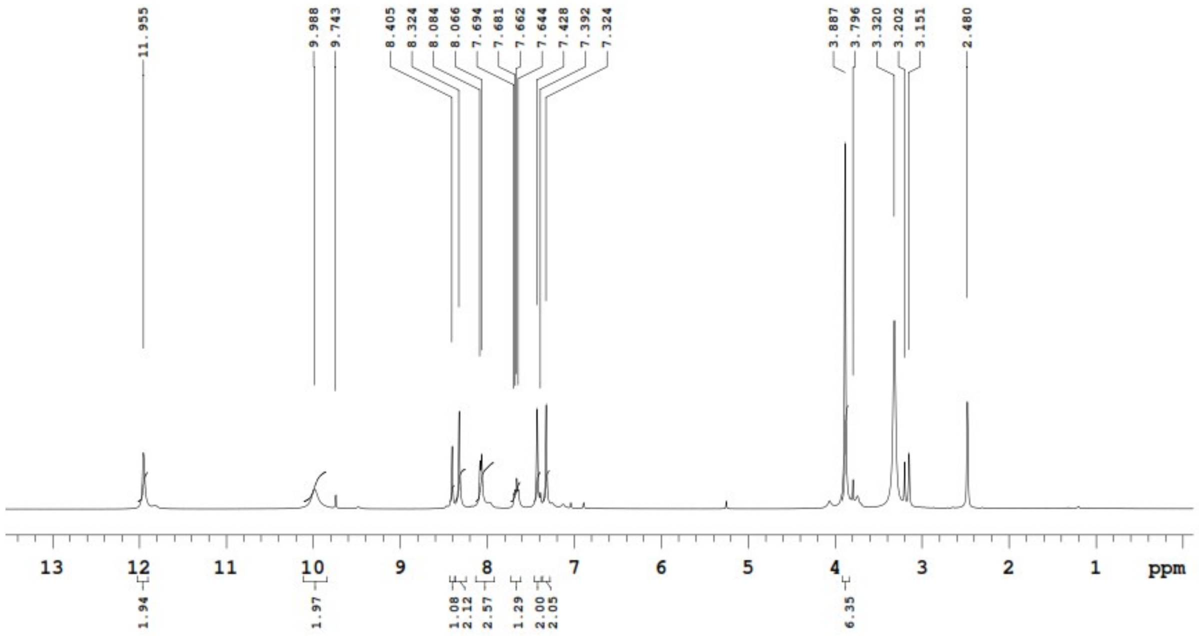

^{1}H NMR spectra of compound no 36

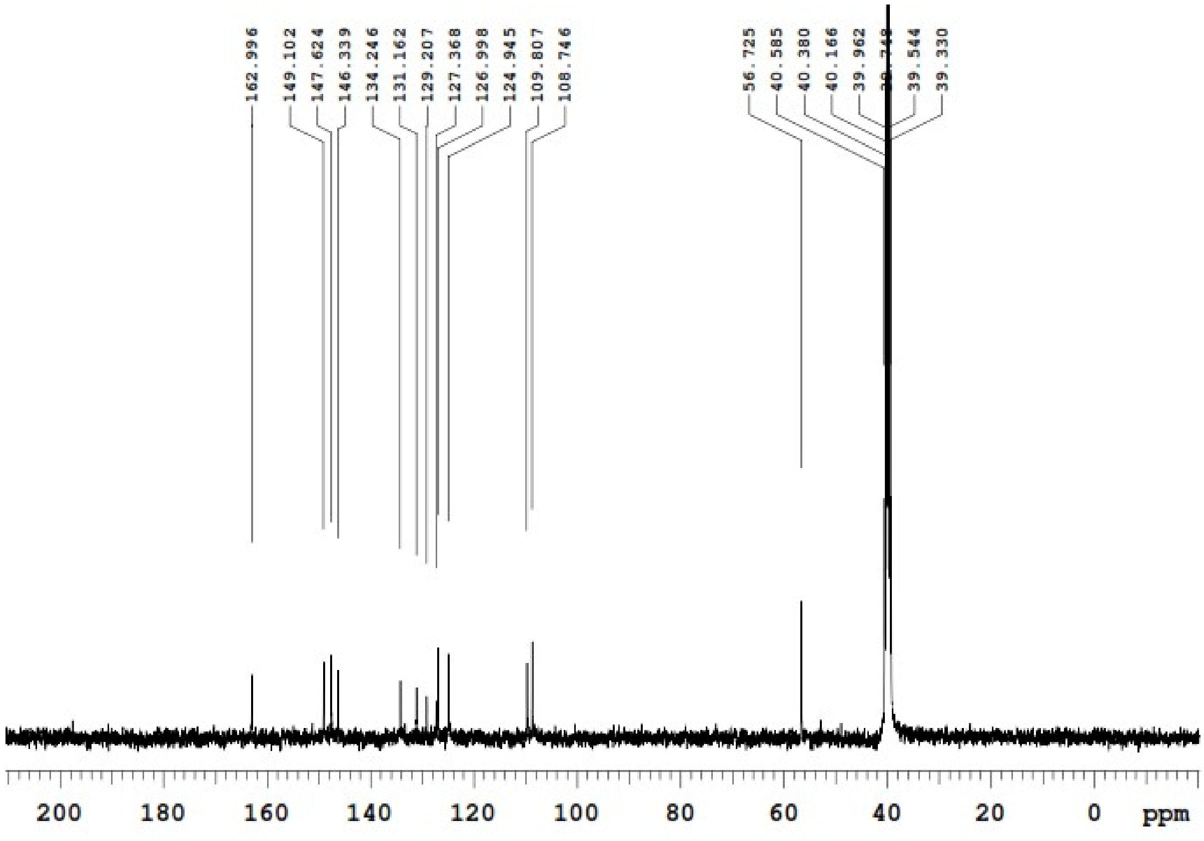

^{13}C NMR spectra of compound no 36
